# Genome Sequencing of the Perciform Fish *Larimichthys crocea* Provides Insights into Molecular and Genetic Mechanisms of Stress Adaptation

**DOI:** 10.1371/journal.pgen.1005118

**Published:** 2015-04-02

**Authors:** Jingqun Ao, Yinnan Mu, Li-Xin Xiang, DingDing Fan, MingJi Feng, Shicui Zhang, Qiong Shi, Lv-Yun Zhu, Ting Li, Yang Ding, Li Nie, Qiuhua Li, Wei-ren Dong, Liang Jiang, Bing Sun, XinHui Zhang, Mingyu Li, Hai-Qi Zhang, ShangBo Xie, YaBing Zhu, XuanTing Jiang, Xianhui Wang, Pengfei Mu, Wei Chen, Zhen Yue, Zhuo Wang, Jun Wang, Jian-Zhong Shao, Xinhua Chen

**Affiliations:** 1 Key Laboratory of Marine Biogenetics and Resources, Third Institute of Oceanography, State Oceanic Administration, Fujian Collaborative Innovation Center for Exploitation and Utilization of Marine Biological Resources, Key Laboratory of Marine Genetic Resources of Fujian Province, Xiamen, P. R. China; 2 College of Life Sciences, ZheJiang University, Key Laboratory for Cell and Gene Engineering of Zhejiang Province, Hangzhou, ZheJiang, P. R. China; 3 BGI-Tech, BGI-Shenzhen, Shenzhen, Guangdong, P. R. China; 4 Ocean University of China, Qingdao, Shandong, P. R. China; 5 College of Life Sciences, Shenzhen University, Shenzhen, Guangdong, P. R. China; University of Wuerzburg, GERMANY

## Abstract

The large yellow croaker *Larimichthys crocea* (*L*. *crocea*) is one of the most economically important marine fish in China and East Asian countries. It also exhibits peculiar behavioral and physiological characteristics, especially sensitive to various environmental stresses, such as hypoxia and air exposure. These traits may render *L*. *crocea* a good model for investigating the response mechanisms to environmental stress. To understand the molecular and genetic mechanisms underlying the adaptation and response of *L*. *crocea* to environmental stress, we sequenced and assembled the genome of *L*. *crocea* using a bacterial artificial chromosome and whole-genome shotgun hierarchical strategy. The final genome assembly was 679 Mb, with a contig N50 of 63.11 kb and a scaffold N50 of 1.03 Mb, containing 25,401 protein-coding genes. Gene families underlying adaptive behaviours, such as vision-related crystallins, olfactory receptors, and auditory sense-related genes, were significantly expanded in the genome of *L*. *crocea* relative to those of other vertebrates. Transcriptome analyses of the hypoxia-exposed *L*. *crocea* brain revealed new aspects of neuro-endocrine-immune/metabolism regulatory networks that may help the fish to avoid cerebral inflammatory injury and maintain energy balance under hypoxia. Proteomics data demonstrate that skin mucus of the air-exposed *L*. *crocea* had a complex composition, with an unexpectedly high number of proteins (3,209), suggesting its multiple protective mechanisms involved in antioxidant functions, oxygen transport, immune defence, and osmotic and ionic regulation. Our results reveal the molecular and genetic basis of fish adaptation and response to hypoxia and air exposure. The data generated by this study will provide valuable resources for the genetic improvement of stress resistance and yield potential in *L*. *crocea*.

## Author Summary

*L*. *crocea* is a temperate-water migratory fish that belongs to the order Perciformes and the family Sciaenidae. In China, the annual yield from *L*. *crocea* aquaculture exceeds that of any other net-cage-farmed marine fish species. *L*. *crocea* also exhibits peculiar behavioral and physiological characteristics and is especially sensitive to various environmental stresses. To understand the molecular and genetic mechanisms underlying the adaptation and response of *L*. *crocea* to environmental stress, we sequenced and assembled the genome of *L*. *crocea*. Further genomic analyses showed the significant expansion of several gene families, such as vision-related crystallins, olfactory receptors, and auditory sense-related genes, and provided a genetic basis for the peculiar physiological characteristics of *L*. *crocea*. Transcriptome analyses of the hypoxia-exposed *L*. *crocea* brain revealed new aspects of neuro-endocrine-immune/metabolism regulatory networks that may help the fish to avoid cerebral inflammatory injury and maintain energy balance under hypoxia. Proteomics data demonstrate that skin mucus of the air-exposed *L*. *crocea* had a complex composition, suggesting its multiple protective mechanisms involved in antioxidant functions, oxygen transport, immune defence, and osmotic and ionic regulation. These findings provide novel insights into the mechanisms of fish stress adaptation.

## Introduction

Teleost fish, nearly half of all living vertebrates, display an amazing level of diversity in body forms, behaviors, physiologies, and environments that they occupy. Strategies for coping with diverse environmental stresses have evolved in different teleost species. Therefore, teleost fish are considered to be good models for investigating the adaptation and response to many natural and anthropogenic environmental stressors [1–3]. Recent genome-sequencing projects in several fish have provided insights into the molecular and genetic mechanisms underlying their responses to some environmental stressors [4–6]. However, to better clarify the conserved and differentiated features of the adaptive response to specific stresses and to trace the evolutionary process of environmental adaptation and response in teleost fish, insight from more teleost species with different evolutionary positions, such as Perciformes, is required. Perciformes are by far the largest and most diverse order of vertebrates, and thus offer a large number of models of adaptation and response to various environmental stresses.

The large yellow croaker, *Larimichthys crocea* (*L*. *crocea*), is a temperate-water migratory fish that belongs to the order Perciformes and the family Sciaenidae. It is mainly distributed in the southern Yellow Sea, the East China Sea, and the northern South China Sea [7]. *L*. *crocea* is one of the most economically important marine fish in China and East Asian countries due to its rich nutrients and trace elements, especially selenium [7, 8]. In China, the annual yield from *L*. *crocea* aquaculture exceeds that of any other net-cage-farmed marine fish species [9,10]. Recently, the basic studies on genetic improvement for growth and disease resistance traits of *L*. *crocea* are increasingly performed for farming purpose [10–13]. *L*. *crocea* also exhibits peculiar behavioral and physiological characteristics, such as loud sound production, high sensitivity to sound, and well-developed photosensitive and olfactory systems [8,14]. Most importantly, *L*. *crocea* is especially sensitive to various environmental stresses, such as hypoxia and air exposure. For example, the response of its brain to hypoxia is quick and robust, and a large amount of mucus is secreted from its skin when it is exposed to air [8,15], although *L*. *crocea* is not exposed to these stress conditions in nature or with standard aquaculture practices. These traits may render *L*. *crocea* a good model for investigating the response mechanisms to environmental stress. Several studies have reported transcriptomic and proteomic responses of *L*. *crocea* to pathogenic infections or immune stimuli [13,16,17]. The effect of hypoxia on the blood physiology of *L*. *crocea* has been evaluated [15]. However, little is known about the molecular response mechanisms of *L*. *crocea* against environmental stress.

To understand the molecular and genetic mechanisms underlying the responses of *L*. *crocea* to environmental stress, we sequenced its whole genome. Furthermore, we sequenced the transcriptome of the hypoxia-exposed *L*. *crocea* brain and profiled the proteome of its skin mucus under exposure to air. Our results revealed the molecular and genetic basis of fish adaptation and response to hypoxia and air exposure.

## Results

### Genome features

We applied a bacterial artificial chromosome (BAC) and whole-genome shotgun (WGS) hierarchical assembly strategy for the *L*. *crocea* genome to overcome the high levels of genome heterozygosity (Table 1 and S1–S2 Fig). The 42,528 BACs were sequenced by the HiSeq 2000 platform and each BAC was assembled by SOAPdenovo [18] (S1 Table). The total length of all combined BACs was 3,006 megabases (Mb), which corresponded to approximately 4.3-fold genome coverage (S2–S3 Tables). All BAC assemblies were then merged into super-contigs and oriented to super-scaffolds with large mate-paired libraries (2–40 kb). Gap filling was made with reads from short insert-sized libraries (170–500 bp) (S3–S4 Tables). In total, we sequenced 563-fold coverage bases of the estimated 691 Mb genome size. The final assembly was 679 Mb, with a contig N50 of 63.11 kb and a scaffold N50 of 1.03 Mb (Table 1). The 672 longest scaffolds (11.2% of all scaffolds) covered more than 90% of the assembly (S5 Table). To assess the completeness of the *L*. *crocea* assembly, 52-fold coverage paired-end high-quality reads were aligned against the assembly (S3 Fig). More than 95.63% of the generated reads could be mapped to the assembly by Burrows-Wheeler Aligner and 98.99% of assembled sequences could be covered by at least 5 reads. Furthermore, the integrity of the assembly was validated by the successful mapping of 95.80% of 18,184 transcripts (greater than 1000 bp) with an identity cutoff of 80% (S6 Table). These results indicate that the genome assembly of *L*. *crocea* has high coverage and is of high quality (S7 Table).

**Table 1:** Summary of the *Larimichthys crocea* genome

Sequencing	Insert Size (bp)	Total Data (Gbp)	Coverage (×)
**BAC**	120 k	324.73	469.94
**WGS**	170–500	36.22	52.42
	2 k–40 k	34.26	49.58
**Assembly**	**N50 (bp)**	**Longest (kbp)**	**Size (Mbp)**
**Contig**	63.11 k	716.89	661.33
**Scaffold**	1.03 M	4,914.79	678.96
**Annotation**	**Number**	**Total Length (Mbp)**	**Percentage Of Genome (%)**
**Repeat**	1,454,906	122.92	18.10
**Gene**	25,401	350.95	51.69
**Exon**	251,617	44.86	6.61

BAC = bacterial artificial chromosome; WGS = whole genome shotgun.

The repetitive elements comprise 18.1% of the *L*.*crocea* genome (S8 Table), which is a relatively low percentage when compared with other fish species, such as *Danio rerio* (52.2%), *Gadus morhua* (25.4%), and *Gasterosteus aculeatus* (25.2%). This suggests that *L*. *crocea* may have a more compact genome (S9–S10 Tables). We identified 25,401 protein-coding genes based on *ab initio* gene prediction and evidence-based searches from the reference proteomes of six other teleost fish and humans (S4 Fig and S11 Table), in which 24,941 genes (98.20% of the whole gene set) were supported by homology or RNAseq evidence (S5 Fig). Over 97.35% of the inferred proteins matched entries in the InterPro, SWISS-PROT, KEGG or TrEMBL database (S12 Table).

### Phylogenetic relationships and genomic comparison

**Fig 1:**
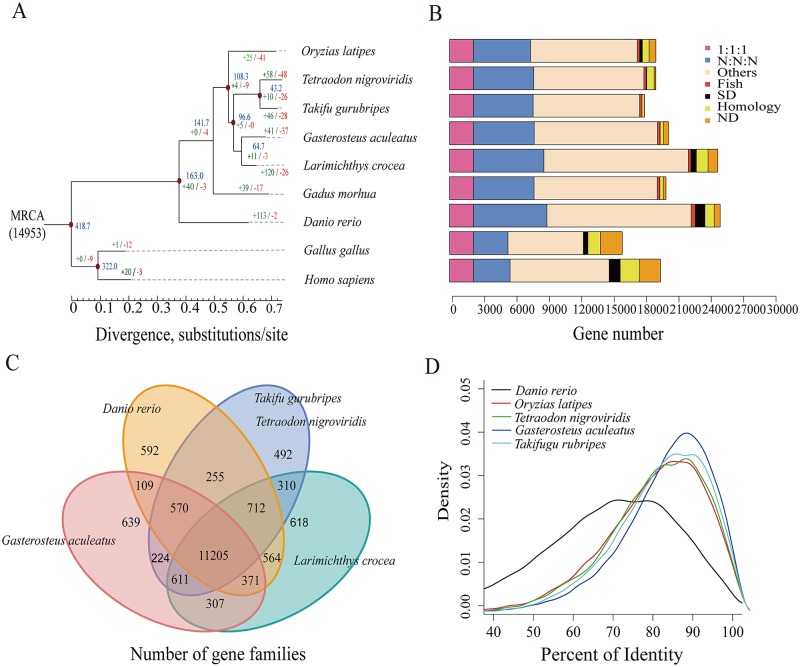
Phylogenetic tree of and orthologous genes in *L*. *crocea* and other vertebrates. (**A**) The phylogenetic tree was constructed from 2,257 single-copy genes with 3.18 M reliable sites by maximum likelihood methods. The red points on six of the internal nodes indicate fossil calibration times in the analysis. Blue numbers indicate the divergence time (Myr, million years ago), and the green and red numbers represent the expanded and extracted gene families, respectively, in *L*. *crocea*. (**B**) The different types of orthologous relationships are shown. “1:1:1” = universal single-copy genes; “N:N:N” = orthologues exist in all genomes; “Fish” = fish-specific genes; “SD” = genes that have undergone species-specific duplication; “Homology” = genes with an e-value less than 1e-5 by BLAST but do not cluster to a gene family; “ND” = species-specific genes; and “Others” = orthologues that do not fit into the other categories. (**C**) The shared and unique gene families in five teleost fish are shown in the Venn diagram. (**D**) Distribution of the identity values of orthologous genes is compared among *L*. *crocea* and other teleosts.

The phylogenetic relationships of *L*. *crocea* to seven other sequenced teleost species were estimated based on 2,257 one-to-one high-quality orthologues, using the maximum likelihood method. According to the phylogeny and the fossil record of teleosts, we dated the divergence of *L*. *crocea* from the other teleost species to approximately 64.7 million years ago (Fig. 1A). We also detected 19,283 orthologous gene families (S3 Table), of which 14,698 families were found in *L*. *crocea*. The gene components of *L*. *crocea* were similar to those of *D*. *rerio* (Fig. 1B). The gene contents in four representative teleost species and *L*. *crocea* genomes were also analysed, and 11,205 (76.23%) gene families were found to be shared by five teleosts (Fig. 1C). We confirmed that the one-to-one orthologous genes of *G*. *aculeatus* and *L*. *crocea* have higher sequence identities from the distribution of the percent identity of proteins (Fig. 1D), which indicates that Sciaenidae has a closer affinity to Gasterosteiformes and coincides with our genome-level phylogeny position.

Furthermore, 121 significantly expanded and 27 contracted gene families (*P* < 0.01) were identified by comparing the family size of *L*. *crocea* with that of the other vertebrates used in the phylogenetic analysis (S14–S15 Tables). Based on the ratios of the number of nonsynonymous substitutions per nonsynonymous site (K_a_) to the number of synonymous substitutions per synonymous site (K_s_; K_a_/K_s_ ratios) in a branch-site model of PAML [19], 92 genes were found to be positively selected in *L*. *crocea* compared with their orthologues in the other six teleost species (*P* < 0.001, S16 Table).

### Genetic features of the *L*. *crocea*

*L*. *crocea* is a migratory fish with good photosensitivity, olfactory detection, and sound perception, and it contains high levels of selenium [8]. Our genomic analyses provide genetic basis for these physiological characteristics. Several crystallin genes (*crygm2b*, *cryba1*, and *crybb3*), which encode proteins that maintain the transparency and refractive index of the lens [4], were significantly expanded in the genome of *L*. *crocea* relative to those of other sequenced teleosts (S17 Table). Phylogenetic analysis showed that the crystallin genes from *L*. *crocea* cluster together, indicating that these genes were specifically duplicated in *L*. *crocea* lineage (S6 Fig). The specific expansion of these crystallin genes may be helpful for improving photosensitivity by increasing lens transparency, thereby enabling the fish to easily find food and avoid predation underwater.

We also identified 112 olfactory receptor (OR)-like genes from the *L*. *crocea* genome (S18 Table and S7 Fig.), and almost all of them (111) have been reported to be expressed in the olfactory epithelial tissues of *L*. *crocea* [14]. The majority of these genes (66) were classified into the “delta” group, which is important for the perception of water-borne odorants [20]. *L*. *crocea* also possessed the highest number of genes that were classified into the “eta” group (30, *P* < 0.001), and these genes may contribute to the olfactory detection abilities, which could be useful for feeding and migration [21].

*L*. *crocea* is named for its ability to generate strong repetitive drumming sounds, especially during reproduction [8]. For good communication, fish have developed high sensitivities to environmental sound. Three important auditory genes, otoferlin (*OTOF*), *claudinj*, and otolin 1 (*OTOL1*), were significantly expanded in the *L*. *crocea* genome (*P* < 0.01, S19 Table). These expansions may contribute to the detection of sound signaling during communication, and thus to reproduction and survival [22].

Selenium is highly enriched in *L*. *crocea* [8], and it is mainly present as selenoproteins. We used the SelGenAmic-based selenoprotein prediction method [23] to analyse the *L*. *crocea* genome and identified 40 selenoprotein genes, which is the highest number among all examined vertebrates (S20 Table). Interestingly, five copies of *MsrB1*, which encodes methionine sulfoxide reductase, were found in *L*. *crocea* (*MsrB1a*, *MsrB1b*, *MsrB1c*, *MsrB1d*, and *MsrB1e*), whereas only two copies (*MsrB1a* and *MsrB1b*) were found in other fish, thus suggesting its broader specificity to reduce all possible substrates [24].

### Characterization of the *L*. *crocea* immune system

**Fig 2:**
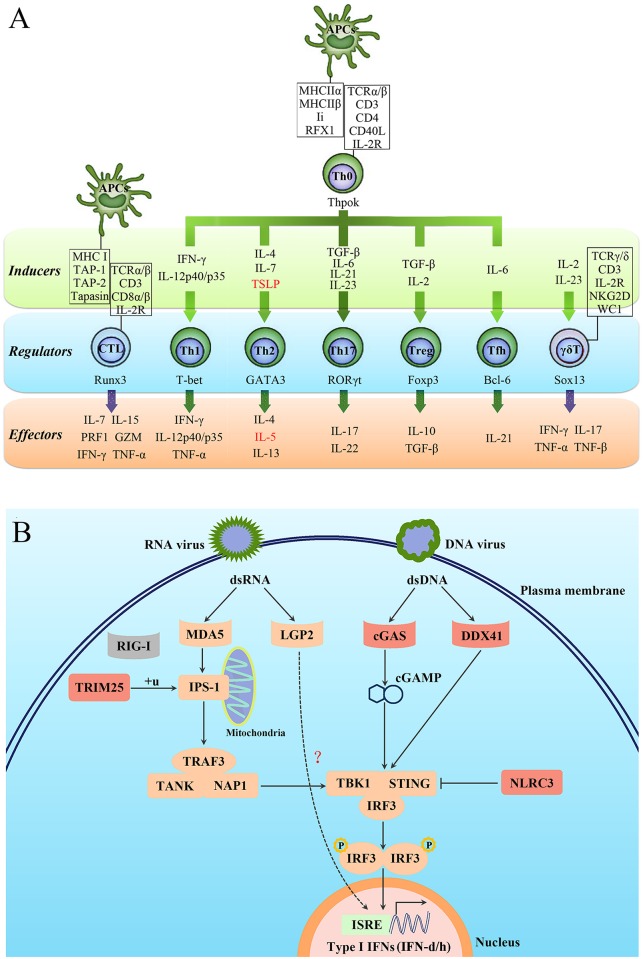
Characterisation of the T-cell lineages in *L*. *crocea* adaptive immunity and the expanded genes in antiviral immunity. (**A**) A schematic diagram summarising genes related to different T-cell lineages in *L*. *crocea* is shown. The inducible factors, the main regulatory transcriptional factors, and the immune effectors of T cells are present in green, blue, and orange backgrounds, respectively. The genes that have been annotated by genome survey are shown in black, and the unannotated genes are shown in red. IL-2R represents all three subunit genes of *L*. *crocea* IL-2 receptor, IL-2RB, IL-2RG and IL-2RA/ IL-15RA genes. The majority of the CD4^+^ T-helper type 1 (Th1), CD4^+^ T-helper type 2 (Th2) and CD8^+^ T cell-related genes have been found in the *L*. *crocea* genome. The genes related to Th17 cell- and γδ-T cell-mediated mucosal immunity are also conserved in *L*. *crocea*. (**B**) Several key genes are expanded in the antiviral immunity pathways in *L*. *crocea*. The genes that have been identified in the *L*. *crocea* genome are shown in orange boxes, and the lost gene (*RIG-I*) is shown in the grey box. LGP2 is able to bind to double-stranded RNA (dsRNA) to trigger interferon production, but the adaptor molecule of LGP2 is still unknown in fish. The red boxes indicate gene families (*TRIM25*, *cGAS*, *DDX41*, and *NLRC3*) that are expanded in *L*. *crocea*. The arrow represents induction, and the interrupted line represents inhibition.

Approximately 2,528 immune-relevant genes were annotated in the *L*. *crocea* genome, including 819 innate immune-relevant genes and 1,709 adaptive immune-relevant genes (S21 Table). Strikingly, *L*. *crocea* was found to have not only a relatively complete innate immune system, but also a well-established adaptive immune system, because the majority of the CD4^+^ T-helper type 1 (Th1), CD4^+^ T-helper type 2 (Th2) and CD8^+^ T cell-related genes were found (Fig. 2A). This suggests that the CD4^+^ Th1, CD4^+^ Th2 and CD8^+^ T cell-mediated adaptive immunity is well conserved in *L*. *crocea*. Moreover, the genes related to Th17 cell- and γδ-T cell-mediated mucosal immune responses were also conserved in *L*. *crocea*, suggesting that a well-developed mucosal immunity exists in this species as well (Fig. 2A). We detected gene expansions in several of these immune-relevant genes, including those encoding lectin receptors (*CLEC17A*), a classical complement component (*C1q*), apoptosis regulator (*BAX*), and immunoglobulins (*IgHV*) (*P* < 0.01, S22 Table). Expansions were also observed in the genes encoding four key proteins for mammalian antiviral immunity: tripartite motif containing 25 (TRIM25), cyclic GMP-AMP synthase (cGAS), DDX41, and NOD-like receptor family CARD domain containing 3 (NLRC3) (Fig. 2B). However, retinoic acid-inducible gene-1 (*RIG-I*), which initiates antiviral signaling pathway by recognizing cytosolic virus-derived RNA in mammals, was not found in the *L*. *crocea* genome and transcriptome [13,16]. The teleost *RIG-I* has been identified only in limited fish species, such as cyprinids and salmonids, and its absence suggests that it may have been lost from particular fish genomes [25]. In mammals, laboratory of genetics and physiology 2 (LGP2) can serve as a suppressor to block RIG-I- and melanoma differentiation-associated protein 5 (MDA5)-elicited signaling [26]. However, LGP2 in fish can bind poly (I:C) to trigger interferon (IFN) production [27], whose functional performance was similar to that done by RIG-I, thereby, LGP2 in fish may act as a substitute for RIG-I (Fig. 2B). The expanded *TRIM25* (54 copies, S8 Fig) may trigger the ubiquitination of IFN-β promoter stimulator-1 (IPS-1), thus allowing for IFN regulatory factor 3 (IRF3) phosphorylation and antiviral signaling initiation [28]. *DDX41* and *cGAS* encode intracellular DNA sensors that activate stimulator of interferon genes (STING) and TANK-binding kinase 1 (TBK1) to induce production of type I IFNs [29,30]. Fish type I IFNs can be divided into two major groups, namely group I and II type I IFNs, with two or four cysteines forming one or two pairs of intramolecular disulphide bonds, respectively [31]. In the *L*. *crocea* genome, two type-I IFN genes, tentatively named IFN-d and IFN-h, were detected, belonging to group I type- I IFNs (Fig. 2B). *L*. *crocea* contained 43 copies of *NLRC3* (S9 Fig), which encodes regulators that prevent type I IFN overproduction [32]. The expansions of these virus-response genes suggest their enhanced roles in innate antiviral immunity, which may explain why *L*. *crocea* is less susceptible to viral infection.

### Stress response under hypoxia

The brain allows rapid and coordinated responses to the environmental stress by driving the secretion of hormones. Therefore, we studied the response of the *L*. *crocea* brain to hypoxia. We sequenced seven transcriptomes of the brains at different times of hypoxia exposure and found that 8,402 genes were differentially expressed at one or more time points (fold change ≥ 2, false discovery rate [FDR] ≤ 0.001; S10 Fig). Hypoxia stress induced a response with the largest number of genes (4,535 genes) at 6 h (S11 Fig), indicating that genes with regulated expression at 6 h may be critical for the response.

**Fig 3:**
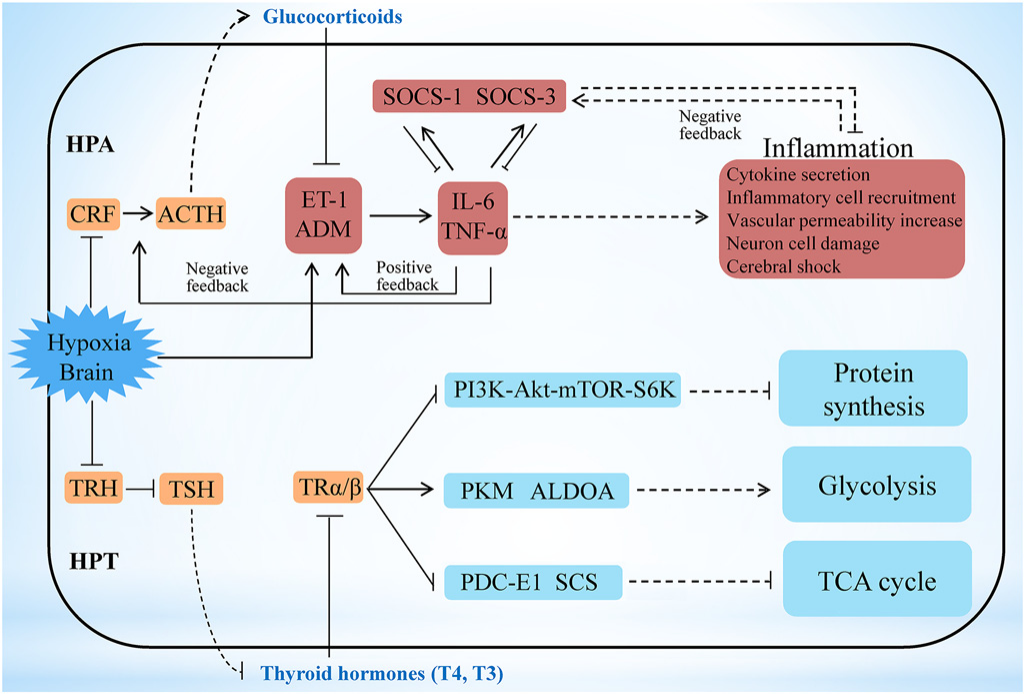
Hypoxia stress exerts responses involving the HPA and HPT axes. Under hypoxia, the potential neuro-endocrine-immune/metabolism networks contribute to the regulation of moderate inflammation and the maintenance of energy balance. Hypoxia may inhibit the hypothalamic-pituitary-adrenal (HPA) axis and induce the expression of ET-1/ADM, and the latter then promote the expression of IL-6/TNF-α and form a positive feedback loop with them. On the other hand, the ET-1/ADM and IL-6/TNF-α may in turn activate the HPA axis, and the latter subsequently induces glucocorticoids and generates a negative feedback to inhibit *ET-1/ADM* and *IL-6/TNF-α* expression to reduce the inflammatory responses in brain. Therefore a HPA axis-ET-1/ADM-IL-6/TNF-α feedback regulatory loop is outlined here. Besides, both SOCS-1 and SOCS-3 may have complementary roles in down-regulating *IL-6* and *TNF-α*, and both IL-6 and TNF-α have reciprocal functions to induce *SOCS-1* and *SOCS-3* expression, thus constituting a SOCS-1/3-dependent feedback regulation to modify cerebral inflammation. The hypothalamic-pituitary-thyroid (HPT) axis was inhibited in *L*. *crocea* brains during the early period of hypoxia, thus leading to a decrease in thyroid hormone (T3 and T4) production. Decrease of HPT axis-thyroid hormones subsequently may inhibit protein synthesis by the PI3K-Akt-mTOR-S6K signaling pathway, which contributes to the reduction in energy consumption during hypoxia stress. Meanwhile, down-regulation of HPT axis-thyroid hormones also represses the tricarboxylic acid (TCA) cycle and accelerates the anaerobic glycolytic pathway, which facilitates O_2_-independent ATP production under hypoxia. Therefore the HPT axis-mediated effects may play roles in response to hypoxia by reorganizing energy consumption and generation. Genes related to the neuro-endocrine system (orange), immunity (red), and metabolic system and protein synthesis (blue) are indicated. The outer border indicates the brain of *L*. *crocea*. The arrow represents promotion, and the interrupted line represents inhibition. Solid lines indicate direct relationships between genes. Dashed lines indicate that more than one step is involved in the process.

Among the numerous incidents happened during the early hypoxia stress, activation of the central neuroimmune system has been shown the most critical event, in which brain neuropeptides, endocrine hormones, and inflammatory cytokines closely participate to generate protective effects [33–35]. However, the precise regulatory networks among these factors have not yet been fully delineated. Our transcriptome analyses show that the key hypothalamic-pituitary-adrenal (HPA) axis-relevant genes (corticotropin-releasing factor [*CRF*], CRF receptor 1 [*CRFR1*], pro-opiomelanocortin [*POMC*], and CRF-binding protein [*CRFBP*]) in the *L*. *crocea* brain displayed a down-up-down-up (W-type) dynamic expression pattern under hypoxia stress (S12 Fig). In contrast, the endothelin-1 (ET-1) and adrenomedullin (ADM) genes showed an up-down-up-down (M-type) dynamic expression pattern, and the time of inflexion point corresponded with that of *CRF*, *CRFR1*, *POMC*, and *CRFBP*. The HPA axis can strictly control the production of glucocorticoids [36,37], and glucocorticoids are suppressors of ET-1 and ADM, both of which are involved in cerebral inflammation in mammals [38,39]. We therefore suggest that a feedback regulatory pathway may exist between the HPA axis and ET-1/ADM under hypoxia stimulation. As a support, the expression of the inflammatory cytokine genes (*IL-6*/*TNF-α*) also showed the M-type pattern and was consistent with that of *ET-1/ADM* (S12 Fig). Expression changes of selected genes, including *CRF*, *CRFR1*, *POMC*, *ET-1*, *ADM* and *IL-6*, during hypoxia stress, were further confirmed by real-time PCR and found to be well corresponding to their expression changes in the transcriptome analyses (S13 Fig). These coordinated and fluctuating expression patterns indicate that hypoxia may inhibit the HPA axis and induce the expression of ET-1/ADM, and the latter then promote the expression of IL-6/TNF-α and form a positive feedback loop with them (Fig. 3). On the other hand, the ET-1/ADM and IL-6/TNF-α may in turn activate the HPA axis, and the latter subsequently induces glucocorticoids and generates a negative feedback to inhibit *ET-1/ADM* and *IL-6/TNF-α* expression to reduce the over-inflammatory responses in brain, the latter of which has been reported in mammals [39–42]. Therefore, our results may outline a novel HPA axis-ET-1/ADM-IL-6/TNF-α feedback regulatory loop in the neuro-endocrine-immune network during hypoxia responses (Fig. 3 and S12–S13 Fig). Besides, we also found that both *SOCS-1* and *SOCS-3* in the *L*. *crocea* brain display opposite expression patterns against *IL-6* and *TNF-α* (S12–S13 Fig). Thus, SOCS-1 and SOCS-3 may have complementary roles in down-regulating *IL-6* and *TNF-α*, and both IL-6 and TNF-α have reciprocal functions to induce *SOCS-1* and *SOCS-3* expression (Fig. 3). These results suggest that a SOCS-1/3-dependent feedback regulation may exist in the process against hypoxia-induced cerebral inflammation in *L*. *crocea*.

Energy maintenance and homeostasis under hypoxia is another critical event in brain. However, the mechanisms underlying this issue remain limited. Here, we found that the major hypothalamic-pituitary-thyroid (HPT) axis-related genes (thyrotropin-releasing hormone [*TRH*], TRH receptor [*TRHR*], thyroid-stimulating hormone β [*TSHβ*], and thyroid hormone receptor α [*TRα*]) were significantly down-regulated in the *L*. *crocea* brain at 1 h to 6 h under hypoxia (S14 Fig). Down-regulation of these gene expressions during hypoxia was also confirmed by real-time PCR (S15 Fig). These findings therefore indicate that the HPT axis may be inhibited during the early period of hypoxia, which agreed with the previous observations that hypoxia could influence the HPT axis [43].

HPT axis can regulate protein synthesis and glucose metabolism by production of thyroid hormones [44,45]. Inhibition of the HPT axis leads to a decrease in the production of thyroid hormones. Thyroid hormones can regulate ribosomal biogenesis and protein translation by the PI3K-Akt-mTOR-S6K signaling pathway [44]. In this study, the mRNA levels of *PI3K*, *S6K*, and most of the components of the protein translation machinery, including the ribosomal proteins and eukaryotic translation initiation factors (*eIF-1*, *-2*, *-3*, *-5* and *-6*), were all down-regulated under hypoxia (S16 Fig). This suggests that the HPT axis may inhibit protein synthesis under hypoxia by decreasing the production of thyroid hormones (Fig. 3), which is beneficial for saving energy during hypoxia stress. Thyroid hormones can also accelerate the oxidative metabolism of glucose and inhibit the glycolytic anaerobic pathway [46]. Interestingly, genes involved in the tricarboxylic acid (TCA) cycle (pyruvate dehydrogenase complex [*PDC-E1*], succinyl-CoA synthetase [*SCS*], and fumarate hydratase [*FH*]) were down-regulated 12 h later under hypoxia, whereas glycolysis-related genes, such as pyruvate kinase (*PKM*), glyceraldehyde 3-phosphate dehydrogenase (*GAPDH*), *GPI*, and aldolase A (*ALDOA*), were greatly increased at 1 h respectively (S14–S15 Fig). The down-regulation of HPT axis-thyroid hormones may inhibit the TCA cycle and accelerate the anaerobic glycolytic pathway in the brain during hypoxia exposure (Fig. 3). The repression of the TCA cycle and the strong induction of the anaerobic glycolytic pathway resulted in a physiological shift from aerobic to anaerobic metabolism, where fish utilise O_2_-independent mechanisms to produce adenosine triphosphate (ATP). However, the mRNA levels of hypoxia-inducible factor (HIF)-1α, which are significantly up-regulated under hypoxia in mammals [47,48], were not significantly changed in the *L*. *crocea* brain (S14 Fig). It is possible that the HIF-1α-mediated mechanism may not be essential for the hypoxia response in the *L*. *crocea* brain during the early period of hypoxia. These results suggest that the HPT axis-mediated effects may play major roles in response to hypoxia by reorganizing energy consumption and energy generation.

### Mucus components and function

The skin mucus is considered as the first defensive barrier between fish and its aquatic environment, and it plays a role in a number of functions, including locomotion, antioxidant responses, respiration, disease resistance, communication, ionic and osmotic regulation [49]. However, the exact mechanisms underlying these functions remain unknown. Mucus is composed mainly of the gel-forming macromolecule mucins and inorganic salts suspended in water [50]. We identified 159 genes that are implicated in mucin biosynthesis and mucus production in the *L*. *crocea* genome (S23 Table), based on previous studies in mammals [51]. This indicates that the mucin synthetic pathway is conserved between fish and mammals. Among these gene families, GALNT, which encodes N-acetylgalactosaminyl transferases [52], was significantly expanded in *L*. *crocea* (27 copies versus 15–20 copies in other fish; S17 Fig). Syntaxin-11 was also expanded. Additionally, genes encoding syntaxin-binding protein 1 and syntaxin-binding protein 5, which are related to mucus secretion, were positively selected in the *L*. *crocea* genome (S16 Table). The expansion and positive selection of these genes may explain why the *L*. *crocea* secretes more mucus than other fish under stress.

**Fig 4:**
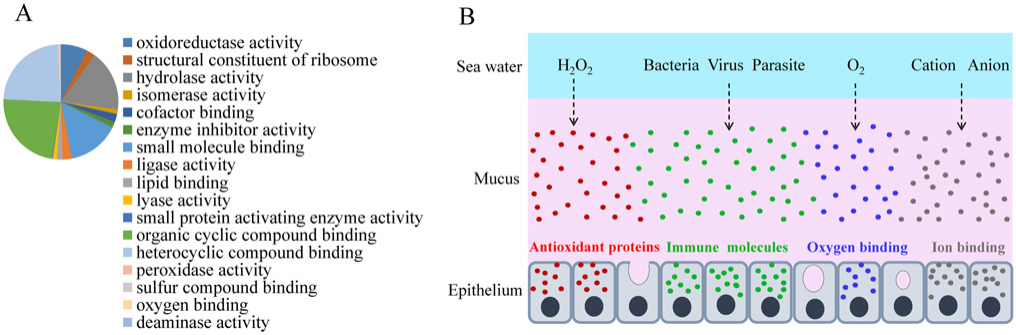
Skin mucus proteins are overexpressed in air-exposed *L*. *crocea*. (**A**) The distribution of mucus proteins in the molecular function class of Gene Ontology is shown. The over-represented functional categories are indicated in the pie chart, of which oxidoreductase activity-, peroxidase activity-, oxygen binding-, and ion binding-related proteins are enriched. (**B**) A representation of the functional mechanisms of the mucus barrier is shown. The continuously replenished thick mucus layer can retain a large number of antioxidant, immune, oxygen-binding, and ion-binding molecules, which are involved in antioxidant functions, immune defence, oxygen transport, and osmotic and ionic regulation, respectively. Antioxidant, immune, oxygen-binding, and ion-binding molecules are indicated in red, green, blue, and gray, respectively.

We identified 22,054 peptides belonging to 3,209 genes in the *L*. *crocea* skin mucus proteome, and this accounted for more than 12% of the protein-coding genes in the genome (S24 Table). The complexity of the *L*. *crocea* mucus presumably relates to the multitude of its biological functions that allow the fish to survive and adapt to environmental changes. The over-represented functional categories were oxidoreductase activity (GO:0016491, *P* = 1.58×10^-35^, 223 proteins), peroxidase activity (GO:0004601, *P* = 0.0075, 9 proteins), oxygen binding (GO:0019825, *P* = 0.0011, 8 proteins), and ion binding (GO:0043167, *P* = 2.21×10^-6^, 347 proteins) (Fig. 4A and S18 Fig.). Two hundred and thirty-two antioxidant proteins that were related to oxidoreductase activity and peroxidase activity were highly enriched in the *L*. *crocea* mucus, and they included peroxiredoxins, glutathione peroxidase, and thioredoxin (S25 Table). These proteins intercept and degrade environmental peroxyl and hydroxyl radicals from aqueous environments [53]. Therefore, the presence of high-abundance antioxidant proteins in the skin mucus may have the potential to protect fish against air exposure-induced oxidative damage (Fig. 4B). Eight proteins related to oxygen transport, including hemoglobin subunits α1, αA, αD, β, and β1, and cytoglobin-1, were identified in the *L*. *crocea* skin mucus (S26 Table). The abundant expression of hemoglobin may contribute to the binding and holding of oxygen for respiration. Various immune molecules that provide immediate protection to fish from potential pathogens, such as lectins, lysozymes, C-reactive proteins, complement components, immunoglobulins, and chemokines, were also found in the *L*. *crocea* skin mucus (S27 Table). To date, the mechanisms of osmotic and ionic regulation of the skin mucus have not been confirmed [49]. In this study, a large number of ion-binding proteins were identified in the *L*. *crocea* mucus (S28 Table). These proteins and the layer of mucus may have a role in limiting the diffusion of ions on the surface of the fish (Fig. 4B). However, a substantial proportion of the proteins, which are highly present in the skin mucus of fish under air exposure, play an unknown role in the mucus response.

## Discussion

We sequenced and assembled the genome of the large yellow croakerr (*L*. *crocea*) using BACs and the WGS hierarchical assembly strategy. This methodology is effective for high-polymorphism genomes and produces a high quality genome assembly, with the 63.11 kb contig N50 and 1.03 Mb scaffold N50 (Table 1). Support from the 563-fold coverage of genome yields high single-base resolution and 95.80% completeness of the coding region (S6 Table). Further genomic analyses showed the significant expansion of several gene families, such as vision-related crystallins, olfactory receptors, and auditory sense-related genes, and provided a genetic basis for the peculiar physiological characteristics of *L*. *crocea*.

During the early stages of hypoxia stress, the induction of ET-1/ADM and IL-6/TNF-α generates the primary protective effect to increase blood pressure, enhance vascular permeability and trigger inflammatory response [54,55]. These mechanisms maintain the brain oxygen supply and resist pathogen infection when the blood brain barrier is disrupted by hypoxia [56]. As the stress response progresses, several natural brakes, including HPA axis-Glucocorticoids and SOCS family members, exhibit secondary protection effects to avoid excessive inflammatory responses in the brain. Our transcriptome results show that a novel HPA axis-ET-1/ADM-IL-6/TNF-α feedback regulatory loop in neuro-endocrine-immune networks contributed to the protective effect and regulated moderate inflammation under hypoxia stress (Fig. 3). On the other hand, the hypoxia-induced down-regulation of the HPT axis may lead to the inhibition of protein synthesis and the activation of anaerobic metabolism (Fig. 3 and S14–S16 Fig). Inhibition of protein synthesis principally contributes to the reduction in cellular energy consumption during hypoxia [3,57]. Activation of anaerobic metabolism facilitates O_2_-independent ATP production under hypoxia, albeit with low ATP yield [57]. Therefore the reduction in ATP consumption through the HPT axis-mediated inhibition of protein synthesis matched the lower ATP yield by the HPT axis-activated anaerobic metabolism, which may aid to maintain cellular energy balance under hypoxia, thus extending fish survival. Hence, our results reveal new aspects of neuro-endocrine-immune/metabolism regulatory networks that may help the fish to avoid cerebral inflammatory injury and maintain energy balance under hypoxia stress. These discoveries will help to improve current understanding of neuro-endocrine-immune/metabolism regulatory networks and protective mechanisms against hypoxia-induced cerebral injury in vertebrates, providing clues for research on the pathogenesis and treatment of hypoxia-induced cerebral diseases.

Amazingly, 3,209 different proteins were identified in the *L*. *crocea* skin mucus under air exposure. Of these, oxidoreductase activity-, oxygen binding-, immunity-, and ion binding-related proteins were enriched (Fig. 4A and S18 Fig). The increase in secretion of the skin mucus of *L*. *crocea* under air exposure may reflect a physiological adjustment of the fish to cope with environmental changes, and the complex components suggest that the skin mucus exerts multiple protective mechanisms, which are involved in antioxidant functions, oxygen transport, immune defence, and osmotic and ionic regulation (Fig. 4B). These results expand our knowledge of skin mucus secretion and function in fish, highlighting its importance in response to stress. In addition, the mucus proteome shares many proteins with the mucus from humans and other animals [58,59]. These characteristics thus make *L*. *crocea* a pertinent model for studying mucus biology.

In summary, our sequencing of the genome of the large yellow croaker provided the genetic basis for its peculiar behavioral and physiological characteristics. Results from transcriptome analyses revealed new aspects of neuro-endocrine-immune/metabolism regulatory networks that may help the fish to avoid cerebral inflammatory injury and maintain energy balance under hypoxia stress. Proteomic profiling suggested that the skin mucus of the fish exhibits multiple protective mechanisms in response to air-exposure stress. Overall, our results revealed the molecular and genetic basis of fish adaptation and response to hypoxia and air exposure. In addition, the data generated by this study will facilitate the genetic dissection of aquaculture traits in this species and provide valuable resources for the genetic improvement of stress resistance and yield potential in *L*. *crocea*.

## Materials and Methods

### Ethics statement

The studies were carried out in strict accordance with the Regulations of the Administration of Affairs Concerning Experimental Animals established by the Fujian Provincial Department of Science and Technology. Animal experiments were approved by the Animal Care and Use Committee of the Third Institute of Oceanography, State Oceanic Administration. All surgery was performed under Tricaine-S anesthesia, and all efforts were made to minimize suffering.

### Genome assembly

The wild *L*. *crocea* individuals were collected from the Sanduao sea area in Ningde, Fujian, China. Genomic DNA was isolated from the blood of a female fish by using standard molecular biology techniques for BAC library construction and sequencing by the HiSeq 2000 Sequencing System in BGI (Beijing Genomics Institute, Shenzhen, China). Subsequently, low-quality and duplicated reads were filtered out, and sequencing errors were removed. The BACs of *L*. *crocea* were assembled by using SOAPdenovo2 [60] (http://soap.genomics.org.cn) with k-mers that ranged from 25 to 63 in size. Then, we selected the assembly with the longest scaffold N50 for gap filling. The BACs were merged together based on the overlap found by BLAT, using the custom script: Rabbit (ftp://ftp.genomics.org.cn/pub/Plutellaxylostella/Rabbit_linux-2.6.18-194.blc.tar.gz). The redundant sequences that were produced by high polymorphisms were removed by sequence depth and shared k-mer percentage. Assembly was performed by scaffolding with mate-paired libraries (2–40 kb) using SSPACE v2 [61], and gap filling was made by Gapcloser (http://sourceforge.net/projects/soapdenovo2/files/GapCloser/) with small-insert libraries (170–500 bp).

The whole-genome sequences of *L*. *crocea* have been deposited in the DNA Data Bank of Japan (DDBJ), the European Molecular Biology Laboratory (EMBL) nucleotide sequencing database and GenBank under the accession JRPU00000000. The version described in this paper is the first version JRPU01000000. All short-read data of WGS and BAC have been deposited in the Short Read Archive (SRA) under accession SRA159210 and SRA159209 respectively.

### Genome annotation

For the annotation of repetitive elements, we used a combination of homology-based and *ab initio* predictions. RepeatMasker [62] and Protein-based RepeatMasking [62] were used to search Repbase, which contains a vast amount of known transcriptional elements at the DNA and protein levels. During the process of *ab initio* prediction, RepeatScout [63] was used to build the *ab initio* repeat library based on k-mer, using the fit-preferred alignment score on the *L*. *crocea* genome. Contamination and multi-copy genes in the library were filtered out before the RepeatScout library was used to find homologs in the genome and to categorise the found repeats by RepeatMasker [62].

Gene models were integrated based on *ab initio* predictions, homologue prediction, and transcription evidence.

### Homology-based prediction

The protein sequences of seven species (*Danio rerio*, *Gasterosteus aculeatus*, *Oreochromis niloticus*, *Oryzias latipes*, *Takifugu rubripes*, *Tetraodon nigroviridis*, and *Homo sapiens*) were aligned to the *L*. *crocea* assembly using BLAST (E-value ≤ 1e-5), and the matches with length coverage >30% of the homologous proteins were considered as gene-model candidates. The corresponding homologous genome sequences were then aligned against the matching proteins by using Genewise [64] to improve gene models.

### *Ab initio* prediction

Augustus [65], SNAP [66], and GENESCAN [67] were used for the *ab initio* predictions of gene structures on the repeat-masked assembly.

### Transcriptome-based prediction

RNAseq reads from the transcriptomes of the mixed tissues of a female and a male (eleven tissues each) were aligned to the genome assembly by Tophat [68], which can identify splice junctions between exons. Cufflinks [69] was used to obtain transcript structures.

Homology-based, *ab initio* derived and transcript gene sets were integrated to form a comprehensive and non-redundant gene set. The overlap length of each gene was verified by different methods, and genes showing 50% overlap by at least one method were selected. To eliminate false positives (genes only supported by *ab initio* methods), novel genes with the reads per kilobase of gene model per million mapped reads (RPKM) ≤ 1 were removed. A method based on gene synteny and FGENESH program (http://www.softberry.com/berry.phtml?topic=fgenesh&group=programs&subgroup=gfind&advanced=on) was used for the prediction of interleukin genes.

### Evolutionary and comparative analyses

To detect variations in the *L*. *crocea* genome, we chose nine species (*Larimichthys crocea*, *Gasterosteus aculeatus*, *Takifugu rubripes*, *Tetraodon nigroviridis*, *Oryzias latipes*, *Gadus morhua*, *Danio rerio*, *Gallus gallus*, and *Homo sapiens*). Proteins that were greater than 50 amino acids in size were aligned by BLAST (-p blastp-e 1e-7), and Treefam [70] was used to construct gene families for comparison.

The 2,257 single-copy genes from the gene family analysis were aligned using MUSCLE [71], and alignments were concatenated as a single data set. To reduce the error topology of phylogeny by alignment inaccuracies, we used Gblock [72] (codon model) to remove unreliably aligned sites and gaps in the alignments. The phylogenetic tree and divergence time were calculated using the PAML 3.0 package [19].

Gene family expansion and contraction analyses were performed by cafe [73]. For optical, olfactory receptor, and auditory system-related genes, we downloaded the genes from Swissprot or Genebank and predicted their candidates using BLAST and Genewise to determinate copy numbers. Pseudogenes produced by frame shift were removed. Phylogenetic analysis of the expanded gene families was based on maximum likelihood methods by PAML 3.0 [19], and the phylogenetic tree was represented by EvolView [74].

Amino acid sequences from six representative teleosts (*Larimichthys crocea*, *Gasterosteus aculeatus*, *Danio rerio*, *Oryzias latipes*, *Takifugu rubripes*, and *Tetraodon nigroviridis*) were aligned by BLAST (-p blastp-e 1e-5-m 8), and reciprocal-best-BLAST-hit methods were used to define orthologous genes in six teleost fish. Because alignment errors are an important concern in molecular data analysis, we made alignments of codon sequences, which are nucleotide sequences that code for proteins, using the PRANK aligner [75]. Positive selection was inferred, based on the branch-site K_a_/K_s_ test by codeml in the PAML 3.0 package [19].

### Transcriptome under hypoxia

*L*. *crocea* (90–100 g) individuals were purchased from a mariculture farm in Ningde, Fujian, China. The fish were maintained at 25°C in aerated water tanks (dissolved oxygen [DO] concentration: 7.8±0.5 mg/L) with a flow-through seawater supply. After 7 days of acclimation, hypoxia-exposure experiments were conducted at 25°C using published methods [3] by bubbling nitrogen gas into an aquarium. The desired concentration of DO was detected by using a DO meter (YSI, Canada). *L*. *crocea* can not maintain the aerobic pathway at DO levels below 2.0 mg/L, and it resorts to anaerobic metabolism [15]. Therefore, at the onset of hypoxia, the oxygen content in the tank was lowered from 7.8±0.5 mg/L to 1.6±0.2 mg/L over a 10-min period. Brains were harvested from six fish at the 0, 1, 3, 6, 12, 24, and 48 h time points and frozen immediately in liquid nitrogen until RNA extraction and transcriptome analyses were performed. To investigate whether the gene expression alterations under hypoxia come from changes in the baseline expression levels, brain tissues were collected from five fish with no hypoxia treatment at several corresponding time points (0, 3, 6, 12, and 24 h) and subjected to real-time PCR analysis of selected genes (S19 Fig).

Total RNA was extracted from the brain tissues harvested at 0, 1, 3, 6, 12, 24, and 48 h after hypoxia exposure using the guanidinium thiocyanate-phenol-chloroform extraction method (Trizol, Invitrogen, USA). The RNA samples (5 μg each) were used to construct RNA-seq libraries using the Illumina mRNA-Seq Prep Kit. The libraries were sequenced by using the Illumina HiSeq 2000 sequencing platform with the paired-end sequencing module [76]. More than 4 gigabase (Gb) of clean data were generated in each library (S29 Table). Clean reads were picked out from the raw reads of each library following removal of reads containing adaptor sequences, reads with an N (unknown bases in read) percentage higher than 5% and low quality reads (>30% of bases with a quantity score Q-value ≤ 10) using an in-house C++ program.

Clean reads were aligned to the *L*. *crocea* genome and its coding sequences (CDS) which were produced from genome-wide prediction of protein-coding genes (S29 Table), with SOAPaligner/SOAP2 [60]. The alignment to the CDS of *L*. *crocea* genome was then utilised to calculate the distribution of reads on reference genes and to perform coverage analysis. If an alignment result passed quality control (alignment ratio > 70%), we proceeded to gene expression calculations and differential expression comparisons. The gene expression levels were calculated based on RPKM values [69], and comparisons of gene expression difference between control (0 h) and each time point after hypoxia exposure (1, 3, 6, 12, 24, and 48 h) were performed using the method described by Audic and Claverie [77]. Genes with fold change ≥ 2 and FDR ≤ 0.001 were defined as differentially expressed genes (DEGs) [78]. Gene expression patterns were analyzed by the open source clustering software Cluster 3.0 and presented using the GraphPad Prism 5 software or Java TreeView. Raw sequencing data for the transcriptome have been deposited in the Gene Expression Omnibus (GEO) under accession GSE57608.

### Real-time PCR analyses of differentially expressed genes under hypoxia

Real-time PCR analysis was performed using the Mastercycler epgradient realplex4 (Eppendorf, Germany) with SYBR Green as the fluorescent dye, according to the manufacturer’s protocol (Takara, China). Primer set was designed based on the coding sequence of each identified gene in *L*. *crocea* genome (S30 Table). Total RNA was extracted from brain tissues of five fish sampled at 0, 1, 3, 6, 12, 24, and 48 h after hypoxia induction. First-strand cDNA was synthesized from 2 μg total RNA and used as a template for real-time PCR. Real-time PCR was performed in a total volume of 20 μL, and cycling conditions were 95°C for 1 min, followed by 40 cycles of 94°C for 5 s, 57°C for 15 s, and 72°C for 20 s. The expression levels of each gene were expressed relative to those of β-actin in each sample using the 2^-ΔΔCT^ method [79]. Each real-time PCR assay was repeated three times. The data of real-time PCR were expressed as the standard error of the mean (SEM). Two-tailed Student’s t test was used for the significance test of the gene expression levels in brain tissues between 0 h and each time point after hypoxia exposure. A *P*-value <0.05 was considered to be statistically significant.

### LC—MS/MS analyses and mucus protein identification

Skin mucus was collected from six healthy *L*. *crocea* individuals under air exposure as previously described [50]. Briefly, the fish were anesthetised with a sub-lethal dose of Tricaine-S (100 mg/L), and transferred gently to a sterile plastic bag for 3 min to slough off the mucus under air exposure. To exclude the cell contamination, mucus was diluted in fresh, cold phosphate-buffered saline and drop-splashed onto slides, which were then air-dried. After staining with 10% Giemsa dye (Sigma, St Louis, MO, USA) for 20 min, the mucus was observed under a Nikon microscope with a 20 × objective. No cell was observed.

Proteins were extracted from a pool of skin mucus of six fish by the trichloroacetic acid-acetone precipitation method and digested by the trypsin gold (Promega, USA). The peptides were then separated by the strong cation exchange chromatography using a Shimadzu LC-20AB HPLC Pump system (Kyoto, Japan). Data acquisition was performed with a Triple TOF 5600 System (AB SCIEX, Concord, ON) fitted with a Nanospray III source (AB SCIEX, Concord, ON). All spectra were mapped by MASCOT server version 2.3.02 against the database of the *L*. *crocea* annotated proteome with the parameters as follows: peptide mass tolerance 0.05 Da; fragment mass tolerance 0.1 Da; fixed modification “Carbamidomethyl (C)”; variable modifications “Gln->pyro-Glu (N-term Q), Oxidation (M), and Deamidation (N, Q)”. The ion score of a peptide matched to a protein must be greater than or equal to the MASCOT identity score, and the peptides must have a length of at least 6 amino acids. For further analysis of the function of the mucus proteome, only the proteins with at least two unique peptides were selected. The mass spectrometry proteomics data have been deposited to the ProteomeXchange Consortium (http://proteomecentral.proteomexchange.org) with the dataset identifier PXD001218.

## Supporting Information

S1 Fig Process of BAC and whole-genome shotgun (WGS) hierarchical strategy. Bacterial artificial chromosome (BAC) and whole-genome shotgun (WGS) hierarchical assembly strategy were applied for the *L*. *crocea* genome to overcome the high levels of genome heterozygosity. BAC sequences were merged to build contig sequences, and whole-genome shotgun sequences (170 bp–40 kbp) were used to build scaffolds and fill gaps.
(PDF)

S2 Fig K-mer analyses. A k-mer refers to an artificial sequence division of K nucleotides iteratively from reads. The k-mer distribution was bimodal, and the k-mer depth of the first peak (22) was half that of the second (44), implying that the genome of *L*. *crocea* was rich in heterogeneous sites. A read with L bp contains (L-K+1) k-mers if the length of each k-mer is K bp. Genome size G is estimated as G = K_num/K_depth. The X-axis is the depth of K-mers derived from the sequenced reads and the Y-axis is the frequency of the K-mer depth.
(PDF)

S3 Fig Depth of single-base distribution based on short-read alignment. To validate the completeness of genome assembly, high-quality reads were aligned against the assembly using Burrows—Wheeler Aligners. A peak was observed at half of the value of the expected peak of 52-fold coverage, suggesting the reluctance of the assemblies. Furthermore, the scaffold sequences with a depth of less than 26× were checked. However, those sequences totaled 3.4 Mb and there were 102 genes (0.04% of total genes) in those scaffolds.
(PDF)

S4 Fig Distribution of intron length, exon number, mRNA length, exon length, and coding region length in the genome of *L*. *crocea* and other related species. (PDF)

S5 Fig Venn diagram representing the *L*. *crocea* gene models supported by the *ab initio* prediction, homology-based methods, and RNAseq-based data. We identified 25,401 protein-coding genes based on *ab initio* gene prediction and evidence-based searches from the reference proteomes of six other teleost fish and humans, in which 24,941 genes (98.20% of the whole gene set) were supported by homology or RNAseq evidence.
(PDF)

S6 Fig Phylogenetic analysis of crystallins in teleosts. Crystallin protein sequences of zebrafish were used to predict crystallin genes in seven other fish species. The phylogenetic tree was constructed by the maximum likelihood method in PAML. Crystallin genes *crygm2b*, *cryba1*, and *crybb3*, which encode proteins that maintain the transparency and refractive index of the lens, were significantly expanded in the genome of *L*. *crocea* relative to those of other sequenced teleosts. The khaki, orange, gold, grey, plum, wheat, and pink backgrounds represent crystallin genes in the genomes of medaka, Atlantic cod, zebrafish, green spotted pufferfish, three spined stickleback, Japanese pufferfish, and large yellow croaker respectively.
(PDF)

S7 Fig Expansion of the olfactory receptor (OR)-like genes of “eta” group in *L*. *crocea* genome. The tree circular cladogram was constructed by the maximum likelihood method in PAML. *L*. *crocea* possessed the highest number of “eta” group olfactory receptor (OR)-like genes (30, *P* < 0.001) relative to those of other sequenced teleosts, which may contribute to the olfactory detection abilities. The blue, khaki, orange, gold, grey, plum, wheat, and pink backgrounds represent the OR-like genes of “eta” group in the genomes of human, medaka, Atlantic cod, zebrafish, green spotted pufferfish, three spined stickleback, Japanese pufferfish, and large yellow croaker respectively.
(PDF)

S8 Fig Expansion of tripartite motif-containing protein 25 (TRIM25) gene family in *L*. *crocea* genome. The tree circular cladogram was constructed by the maximum likelihood method in PAML. The blue, khaki, orange, grey, plum, wheat, and pink backgrounds represent TRIM25 genes in the genomes of human, medaka, Atlantic cod, green spotted pufferfish, three spined stickleback, Japanese pufferfish, and large yellow croaker respectively.
(PDF)

S9 Fig Expansion of NOD-like receptor family CARD domain containing 3 (NLRC3) gene family in *L*. *crocea* genome. The tree circular cladogram was constructed by the maximum likelihood method in PAML. The blue, khaki, orange, grey, plum, wheat, and pink backgrounds represent NLRC3 genes in the genomes of human, medaka, Atlantic cod, green spotted pufferfish, three spined stickleback, Japanese pufferfish, and large yellow croaker respectively.
(PDF)

S10 Fig Differentially expressed genes (DEGs) in the *L*. *crocea* brains under hypoxic and normal conditions. We define the fold change ≥2 and FDR ≤0.001 as significant DEGs. (**A**) The 5564 DEGs were significantly down-regulated at more than one time point after hypoxia exposure and not significantly up-regulated at other time points. (**B**) The 1948 DEGs were significantly up-regulated at more than one time point after hypoxia exposure and not significantly down-regulated at other time points. (**C**) The 890 DEGs were significantly up-regulated at some time points and significantly down-regulated at other time points under hypoxia.
(PDF)

S11 Fig Number of differentially expressed genes (DEGs) at different time points under hypoxia. The comparisons of gene expression difference between control (0 h) and each time point after hypoxia induction (1, 3, 6, 12, 24, and 48 h) were performed using the method described by Audic and Claverie [77]. The significant DEGs are defined as fold change ≥2 and FDR ≤0.001. The Y-axis represents the number of differentially expressed genes under hypoxia; The X-axis represents the time of hypoxia induction. Hypoxia stress induced a response with the largest number of genes (4,535 genes) at 6 h, indicating that genes with regulated expression at 6 h may be critical for the response.
(PDF)

S12 Fig The dynamic expression patterns of the genes involved in potential neuro-endocrine-immunity network in the *L*. *crocea* brain under hypoxia. The gene expression levels were calculated based on RPKM values [69], and comparisons of gene expression difference between control (0 h) and each time point after hypoxia exposure (1, 3, 6, 12, 24, and 48 h) were performed using the method described by Audic and Claverie [77]. Genes with fold change ≥ 2 and FDR ≤ 0.001 were defined as differentially expressed genes (DEGs). Gene expression patterns were analyzed by the open source clustering software Cluster 3.0 and presented using the GraphPad Prism 5 software. The Y-axis is log_2_ ratio of fold changes relative to control. The key hypothalamic-pituitary-adrenal (HPA) axis-relevant genes, including corticotropin-releasing factor (*CRF*), CRF receptor 1 (*CRFR1*), pro-opiomelanocortin (*POMC*), and CRF-binding protein (*CRFBP*) displayed a down-up-down-up (W-type) dynamic expression pattern under hypoxia stress. In contrast, the endothelin-1 (ET-1) and adrenomedullin (ADM) genes showed an up-down-up-down (M-type) dynamic expression pattern, and the time of inflexion point corresponded with that of *CRF*, *CRFR1*, *POMC*, and *CRFBP*. The expression of the inflammatory cytokine genes (*IL-6*/*TNF-α*) also showed the M-type pattern and was consistent with that of *ET-1/ADM*. Besides, both *SOCS-1* and *SOCS-3* in the *L*. *crocea* brain display opposite expression patterns against *IL-6* and *TNF-α*.
(PDF)

S13 Fig Real-time PCR analysis of selected differentially expressed genes identified in the brain transcriptomes of hypoxia-induced *L*. *crocea*. Total RNA was extracted from the brain tissues of *L*. *crocea* collected at 0, 1, 3, 6, 12, 24 and 48 h after hypoxia induction. Expression levels of selected genes involved in neuro-endocrine-immunity network (*CRF*, *CRFR1*, *POMC*, *ET-1*, *ADM*, *IL-6*, *SOCS-1* and *SOCS-3*) in the brain tissues at each time point after hypoxia induction were detected by real-time PCR. The expression levels of each gene were expressed relative to those of β-actin in each sample using the 2^-ΔΔCT^ method [79]. Each real-time PCR assay was repeated three times. The data of real-time PCR were expressed as the standard error of the mean (SEM). Two-tailed Student’s t test was used for the significance test of the gene expression levels in brain tissues between 0 h and each time point after hypoxia exposure. ^*^ *P*< 0.05, ^**^ *P*< 0.01.
(PDF)

S14 Fig Expression profiles of the genes involved in potential neuro-endocrine-metabolism network in the *L*. *crocea* brain under hypoxia. The gene expression levels were calculated based on RPKM values [69], and comparisons of gene expression difference between control (0 h) and each time point after hypoxia exposure (1, 3, 6, 12, 24, and 48 h) were performed using the method described by Audic and Claverie [77]. Genes with fold change ≥ 2 and FDR ≤ 0.001 were defined as differentially expressed genes (DEGs). Gene expression patterns were analyzed by the open source clustering software Cluster 3.0 and presented using the Java TreeView. Genes shown in red are up-regulated, and those shown in green are down-regulated, relative to the control. Genes with no expression are shown in gray. Values in toolbar are log_2_ ratio of fold changes relative to control.
(PDF)

S15 Fig Real-time PCR analysis of selected differentially expressed genes identified in the brain transcriptomes of hypoxia-induced *L*. *crocea*. Total RNA was extracted from the brain tissues of *L*. *crocea* collected at 0, 1, 3, 6, 12, 24, and 48 h after hypoxia induction. Expression levels of selected genes involved in neuro-endocrine-metabolism network (*TRH*, *TRHR*, *TSHβ*, *TRα*, *PDC E1α*, *SCS*, *FH*, and *ALDOA*) in the brain tissues at each time point after hypoxia induction were detected by real-time PCR. The expression levels of each gene were expressed relative to those of β-actin in each sample using the 2^-ΔΔCT^ method [79]. Each real-time PCR assay was repeated three times. The data of real-time PCR were expressed as the standard error of the mean (SEM). Two-tailed Student’s t test was used for the significance test of the gene expression levels in brain tissues between 0 h and each time point after hypoxia exposure. ^*^ *P*< 0.05, ^**^ *P*< 0.01.
(PDF)

S16 Fig Expression profiles of protein synthesis-related genes in the *L*. *crocea* brain under hypoxia. The gene expression levels were calculated based on RPKM values [69], and comparisons of gene expression difference between control (0 h) and each time point after hypoxia exposure (1, 3, 6, 12, 24, and 48 h) were performed using the method described by Audic and Claverie [77]. Genes with fold change ≥ 2 and FDR ≤ 0.001 were defined as differentially expressed genes (DEGs). Gene expression patterns were analyzed by the open source clustering software Cluster 3.0 and presented using the Java TreeView. Genes shown in red are up-regulated, and those shown in green are down-regulated, relative to the control. Genes with no expression are shown in gray. Values in toolbar are log_2_ ratio of fold changes relative to control.
(PDF)

S17 Fig Expansion of the N-acetylgalactosaminyl transferase (GALNT) gene family in *L*. *crocea* genome. Distribution of GALNTs 1–14 in the genomes of human, zebrafish, stickleback, Japanese pufferfish, green spotted pufferfish, and large yellow croaker is shown.
(PDF)

S18 Fig Enrichment of Gene Ontology categories for skin mucus proteins. Here we applied the EnrichPipeline to extract annotation information in Gene Ontology with *P*<0.01. The functions are summarized in three main categories: biological process, cellular component, and molecular function.
(PDF)

S19 Fig Real-time PCR analysis of selected genes in the brain tissues of untreated *L*. *crocea*. Total RNA was extracted from *L*. *crocea* brain tissues collected at 0, 3, 6, 12, and 24 h with no hypoxia treatment. Expression levels of six selected genes (*CRF*, *CRFR1*, *SOCS1*, *TRHR*, *SCS*, and *FH*), which were identified as differentially expressed genes in the brain transcriptomes of hypoxia-induced *L*. *crocea* (S12 and S14 Fig.), in the untreated brain tissues at each time point were detected by real-time PCR. The expression levels of each gene were expressed relative to those of β-actin in each sample using the 2^-ΔΔCT^ method [79]. Each real-time PCR assay was repeated three times. The data of real-time PCR were expressed as the standard error of the mean (SEM). Two-tailed Student’s t test was used for the significance test of the gene expression levels in brain tissues between 0 h and each later time point. The results showed that the expression levels of these six genes were not significantly changed between 0 h and each later time point in brain tissues of untreated fish, suggesting that the gene expression alterations under hypoxia should not come from change in the baseline expression levels.
(PDF)

S1 Table Summary of BACs used in *L*. *crocea* genome project. (PDF)

S2 Table Summary of k-mer analysis. (PDF)

S3 Table Stastics of BAC sequences used for mergence. (PDF)

S4 Table Information of whole-genome shotgun reads. (PDF)

S5 Table Statistics of final assembly. (PDF)

S6 Table Genome assembly validation by transcripts mapping. (PDF)

S7 Table Summary of genome assembly of seven sequenced teleost species. (PDF)

S8 Table Summary of repetitive elements in *L*. *crocea* genome. (PDF)

S9 Table Top ten transposable elements (TE) in seven teleost species. (PDF)

S10 Table Comparison of repeat content from nine sequenced vertebrate species. (PDF)

S11 Table Statistics of predicted protein-coding genes. (PDF)

S12 Table Functional classification of *L*. *crocea* genes. (PDF)

S13 Table Summary of gene families in the genomes of nine sequenced vertebrate species by Treefam. (PDF)

S14 Table Gene Ontology of expanded gene families in *L*. *crocea* genome. (PDF)

S15 Table Gene Ontology of contracted gene families in *L*. *crocea* genome. (PDF)

S16 Table Positive selection genes in *L*. *crocea* genome. (PDF)

S17 Table Copy number of vision-related genes in seven sequenced teleost species. (PDF)

S18 Table Olfactory receptor-like gene repertoire in seven sequenced teleost species. (PDF)

S19 Table Copy number of auditory sense-related genes in seven sequenced teleost species. (PDF)

S20 Table Comparison of the genes encoding for selenoproteins between *L*. *crocea* and other sequenced vertebrate species. (PDF)

S21 Table Characterization of the *L*. *crocea* immune system. (PDF)

S22 Table Number of genes related to immunity in *L*. *crocea* and other six fish genomes. (PDF)

S23 Table Genes involved in mucin biosynthesis and mucus production. (PDF)

S24 Table Summary of MS/MS spectra and proteins identified in the *L*. *crocea* skin mucus under air exposure. (PDF)

S25 Table Antioxidant proteins identified in the *L*. *crocea* mucus proteome. (PDF)

S26 Table Oxygen binding-related proteins identified in the *L*. *crocea* mucus proteome. (PDF)

S27 Table Immunity-related proteins identified in the *L*. *crocea* mucus proteome. (PDF)

S28 Table Number of ion binding-related proteins identified in the *L*. *crocea* mucus proteome. (PDF)

S29 Table Summary of the data for transcriptomes under hypoxia and read alignment ratios to *L*. *crocea* genome and its CDS. (PDF)

S30 Table Primer sequences for real-time PCR. (PDF)

S1 Text Background materials. (PDF)
