# Supplementary figures and images for: Genome Sequencing of the Perciform Fish Larimichthys crocea Provides Insights into Molecular and Genetic Mechanisms of Stress Adaptation
Source: PLoS Genet. 2015 Apr 2;11(4):e1005118. doi: 10.1371/journal.pgen.1005118 (PMC4383535; doi:10.1371/journal.pgen.1005118)

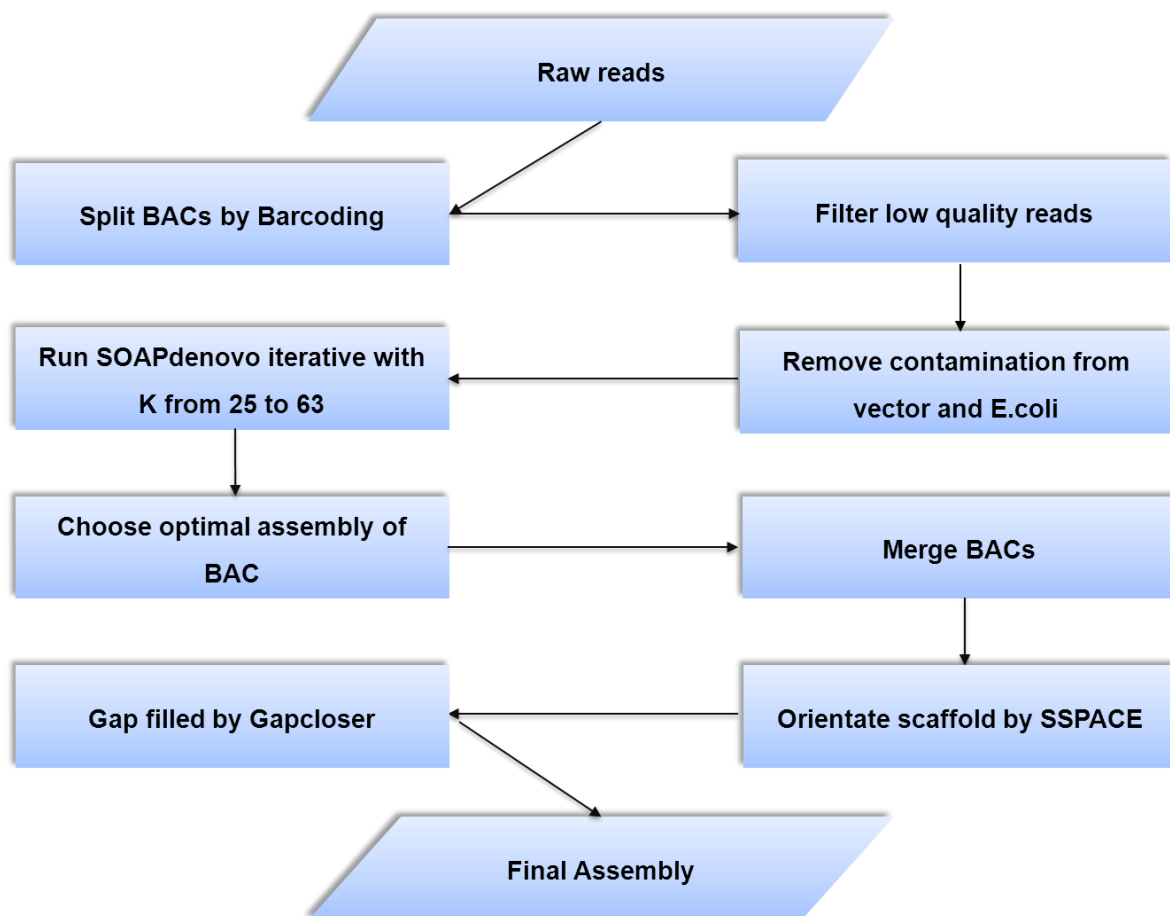

Supplement: S1 Fig — Bacterial artificial chromosome (BAC) and whole-genome shotgun (WGS) hierarchical assembly strategy were applied for the L. crocea genome to overcome the high levels of genome heterozygosity. BAC sequences were merged to build contig sequences, and whole-genome shotgun sequences (170 bp–40 kbp) were used to build scaffolds and fill gaps. (PDF) [file pgen.1005118.s001.pdf]

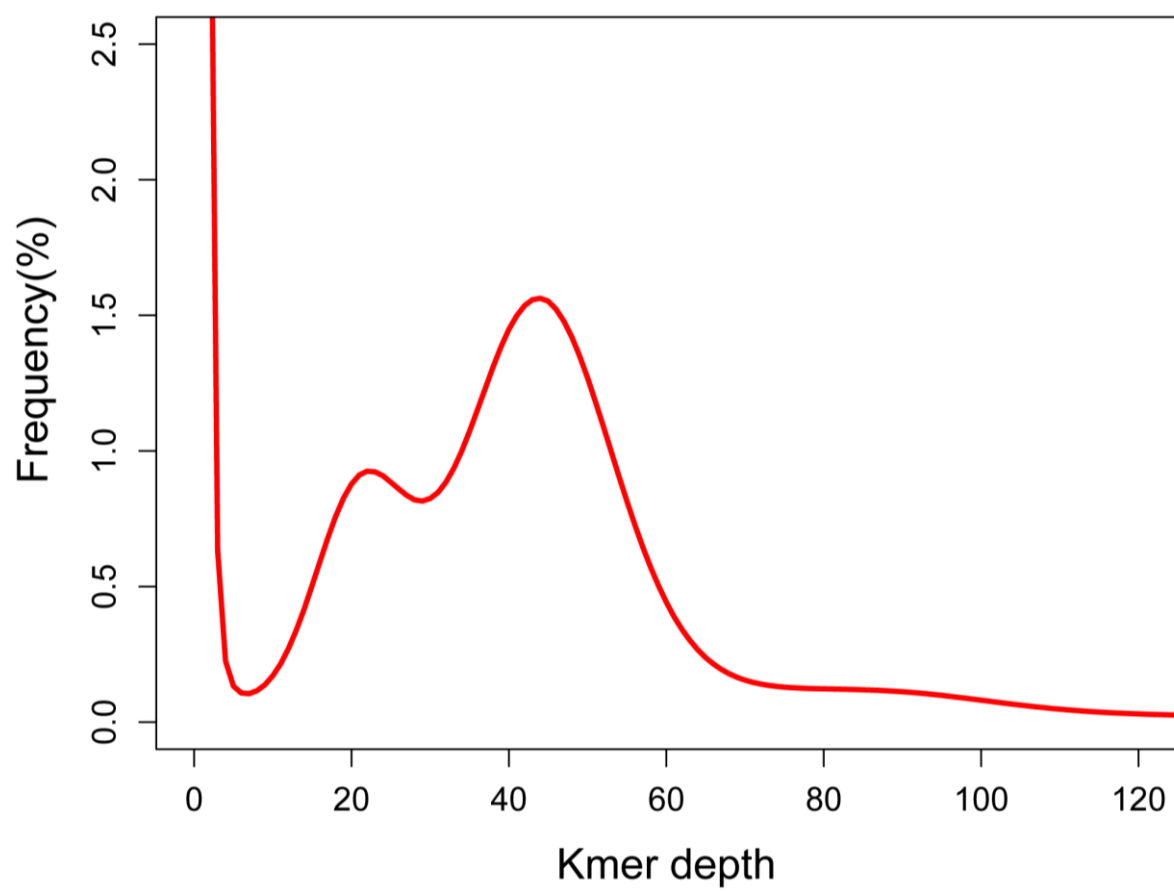

Supplement: S2 Fig — A k-mer refers to an artificial sequence division of K nucleotides iteratively from reads. The k-mer distribution was bimodal, and the k-mer depth of the first peak (22) was half that of the second (44), implying that the genome of L. crocea was rich in heterogeneous sites. A read with L bp contains (L-K+1) k-mers if the length of each k-mer is K bp. Genome size G is estimated as G = K_num/K_depth. The X-axis is the depth of K-mers derived from the sequenced reads and the Y-axis is the frequency of the K-mer depth. (PDF) [file pgen.1005118.s002.pdf]

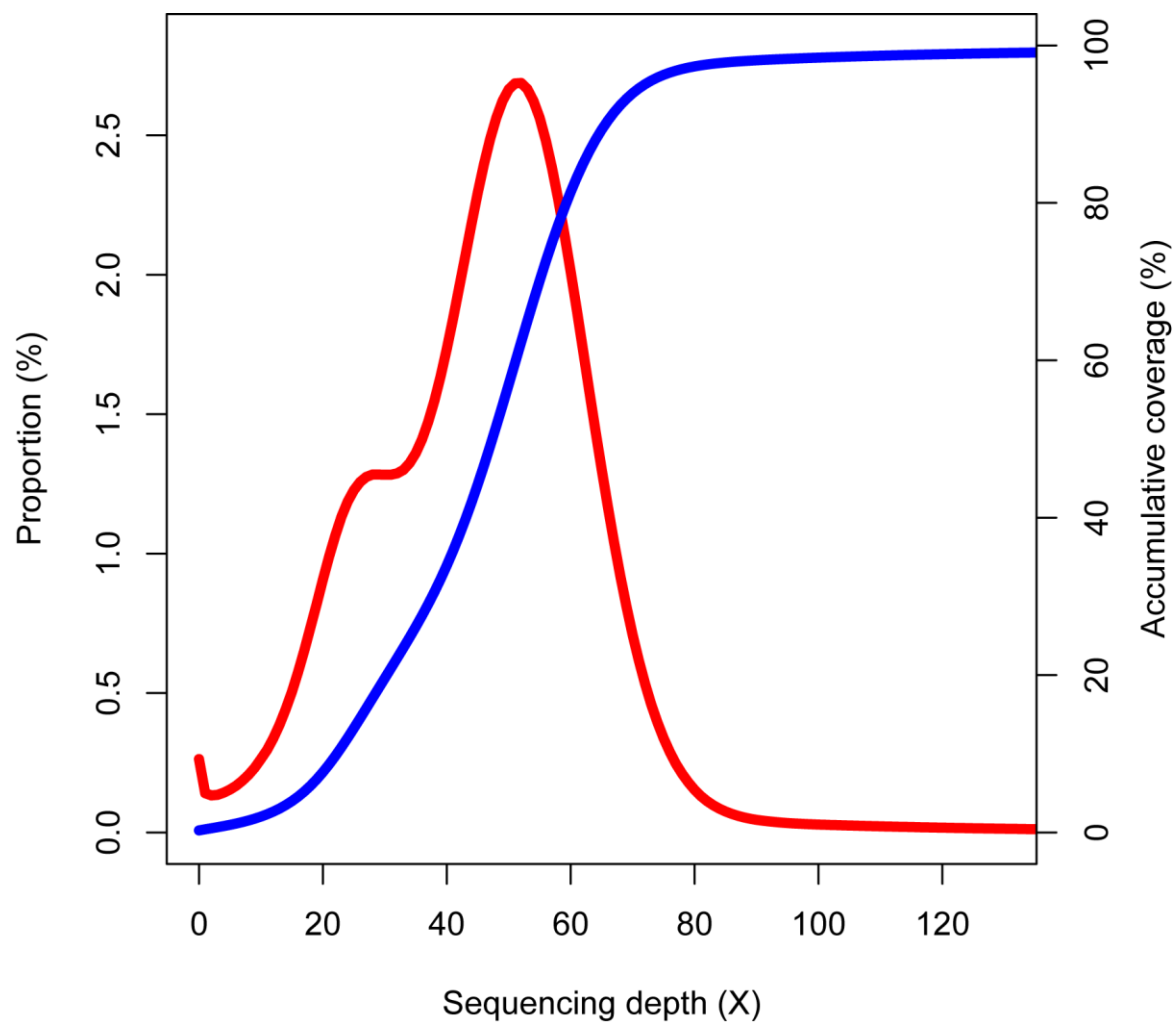

Supplement: S3 Fig — To validate the completeness of genome assembly, high-quality reads were aligned against the assembly using Burrows—Wheeler Aligners. A peak was observed at half of the value of the expected peak of 52-fold coverage, suggesting the reluctance of the assemblies. Furthermore, the scaffold sequences with a depth of less than 26× were checked. However, those sequences totaled 3.4 Mb and there were 102 genes (0.04% of total genes) in those scaffolds. (PDF) [file pgen.1005118.s003.pdf]

*Ab initio*

**Homolog**

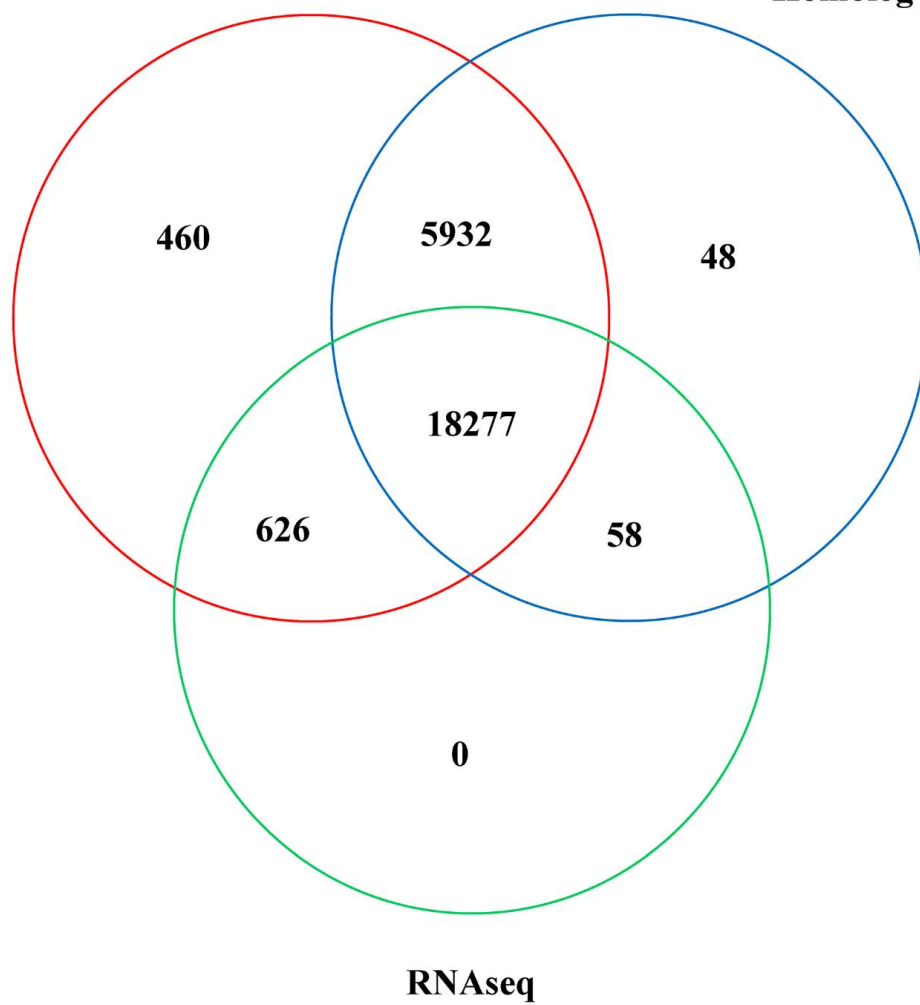

Supplement: S5 Fig — We identified 25,401 protein-coding genes based on ab initio gene prediction and evidence-based searches from the reference proteomes of six other teleost fish and humans, in which 24,941 genes (98.20% of the whole gene set) were supported by homology or RNAseq evidence. (PDF) [file pgen.1005118.s005.pdf]

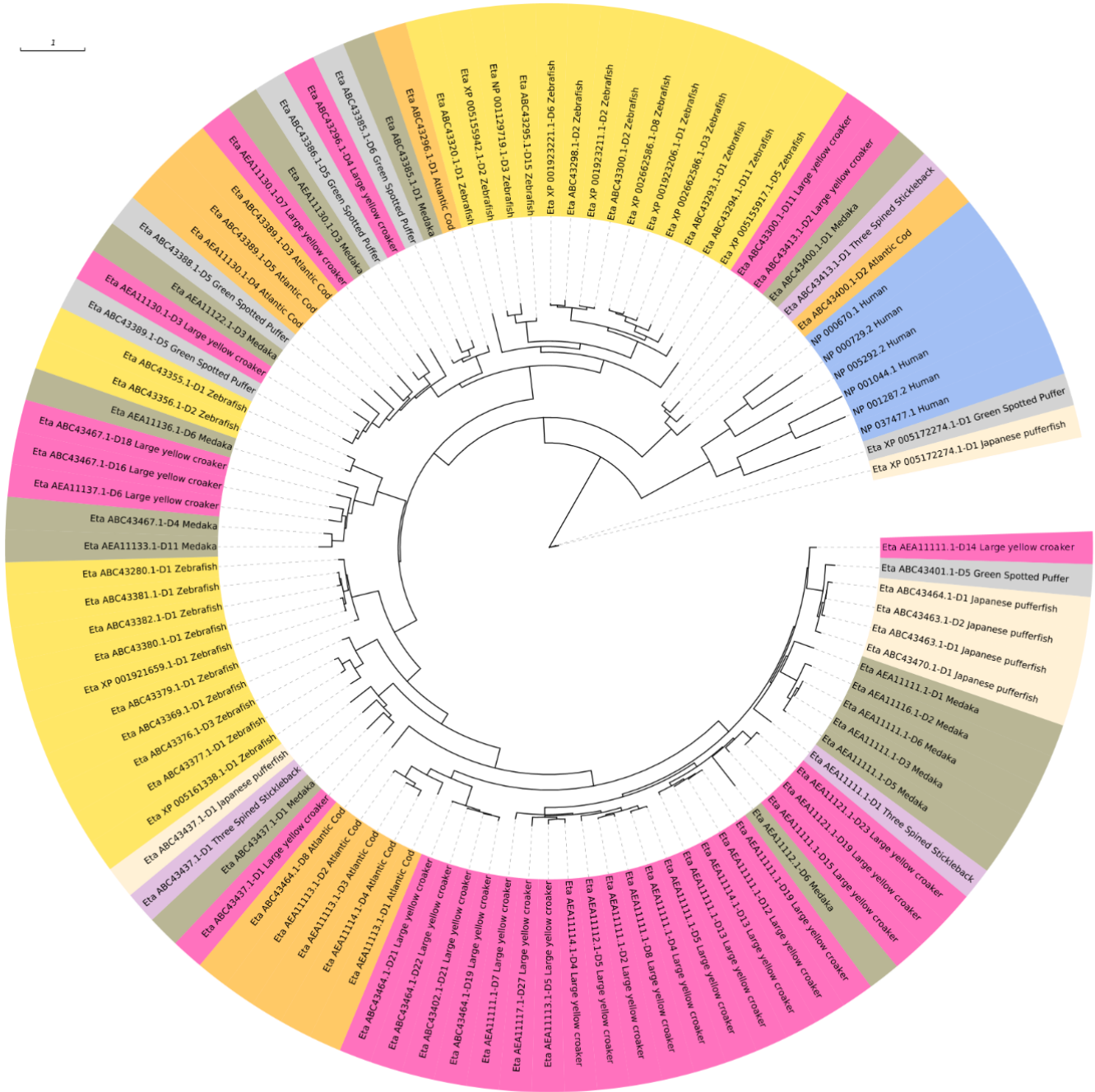

Supplement: S7 Fig — The tree circular cladogram was constructed by the maximum likelihood method in PAML. L. crocea possessed the highest number of “eta” group olfactory receptor (OR)-like genes (30, P < 0.001) relative to those of other sequenced teleosts, which may contribute to the olfactory detection abilities. The blue, khaki, orange, gold, grey, plum, wheat, and pink backgrounds represent the OR-like genes of “eta” group in the genomes of human, medaka, Atlantic cod, zebrafish, green spotted pufferfish, three spined stickleback, Japanese pufferfish, and large yellow croaker respectively. (PDF) [file pgen.1005118.s007.pdf]

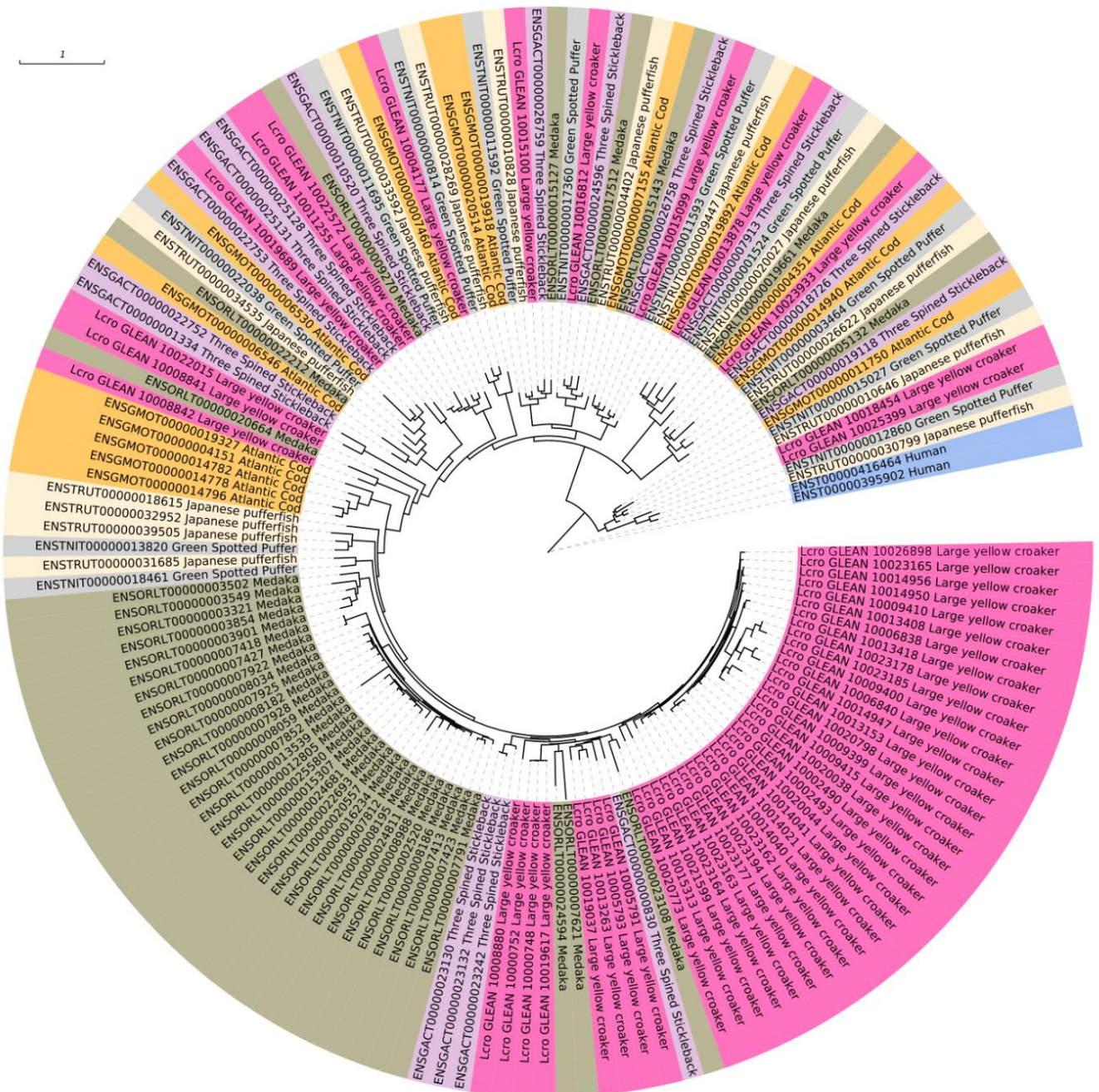

Supplement: S8 Fig — The tree circular cladogram was constructed by the maximum likelihood method in PAML. The blue, khaki, orange, grey, plum, wheat, and pink backgrounds represent TRIM25 genes in the genomes of human, medaka, Atlantic cod, green spotted pufferfish, three spined stickleback, Japanese pufferfish, and large yellow croaker respectively. (PDF) [file pgen.1005118.s008.pdf]

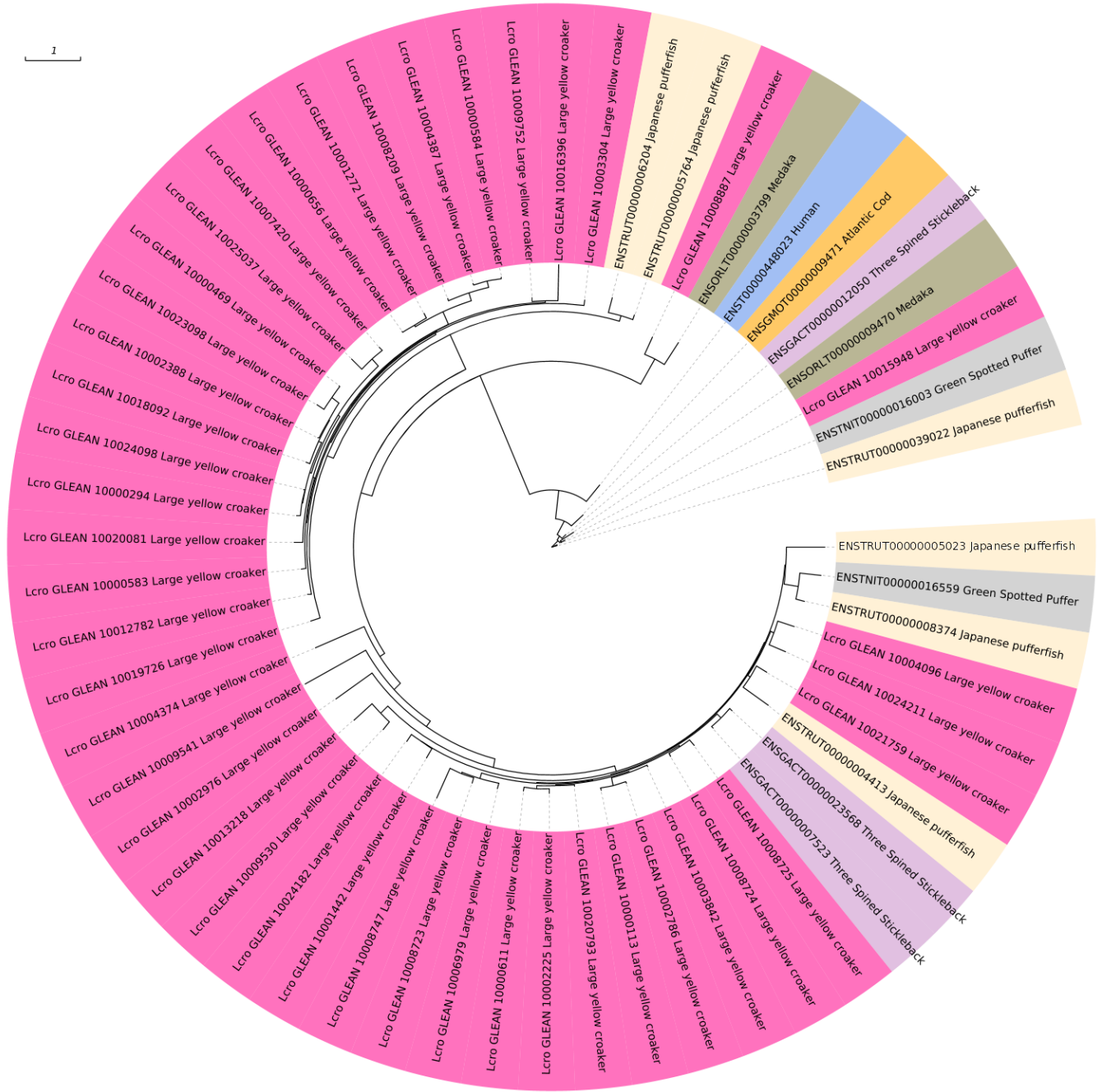

Supplement: S9 Fig — The tree circular cladogram was constructed by the maximum likelihood method in PAML. The blue, khaki, orange, grey, plum, wheat, and pink backgrounds represent NLRC3 genes in the genomes of human, medaka, Atlantic cod, green spotted pufferfish, three spined stickleback, Japanese pufferfish, and large yellow croaker respectively. (PDF) [file pgen.1005118.s009.pdf]

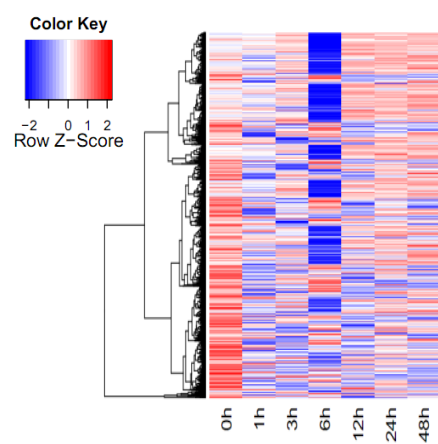

A

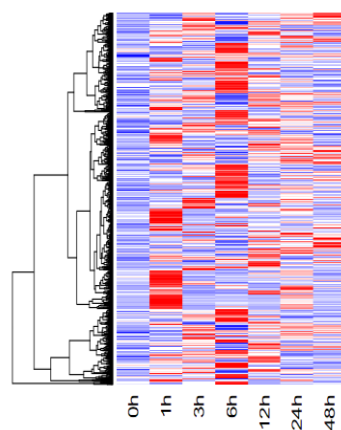

B

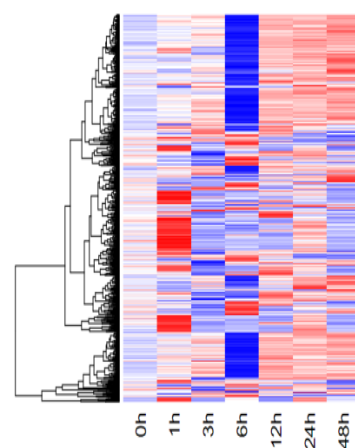

C

Supplement: S10 Fig — We define the fold change ≥2 and FDR ≤0.001 as significant DEGs. (A) The 5564 DEGs were significantly down-regulated at more than one time point after hypoxia exposure and not significantly up-regulated at other time points. (B) The 1948 DEGs were significantly up-regulated at more than one time point after hypoxia exposure and not significantly down-regulated at other time points. (C) The 890 DEGs were significantly up-regulated at some time points and significantly down-regulated at other time points under hypoxia. (PDF) [file pgen.1005118.s010.pdf]

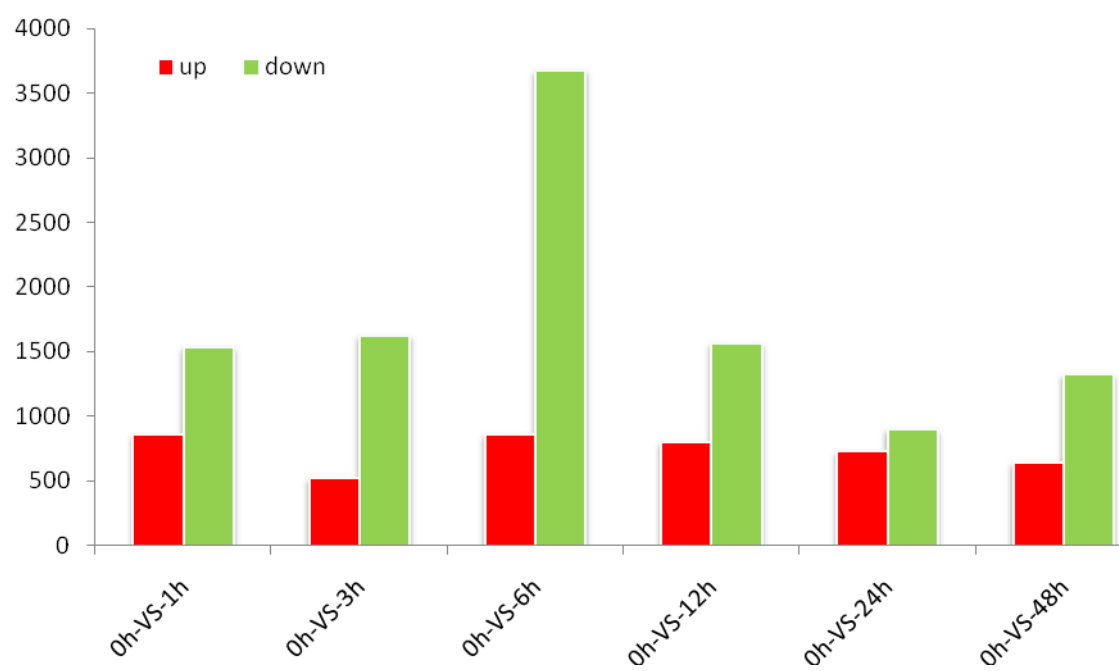

Supplement: S11 Fig — The comparisons of gene expression difference between control (0 h) and each time point after hypoxia induction (1, 3, 6, 12, 24, and 48 h) were performed using the method described by Audic and Claverie [77]. The significant DEGs are defined as fold change ≥2 and FDR ≤0.001. The Y-axis represents the number of differentially expressed genes under hypoxia; The X-axis represents the time of hypoxia induction. Hypoxia stress induced a response with the largest number of genes (4,535 genes) at 6 h, indicating that genes with regulated expression at 6 h may be critical for the response. (PDF) [file pgen.1005118.s011.pdf]

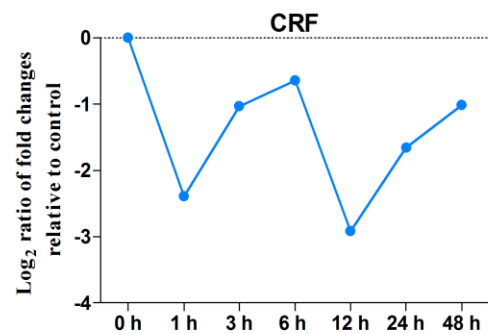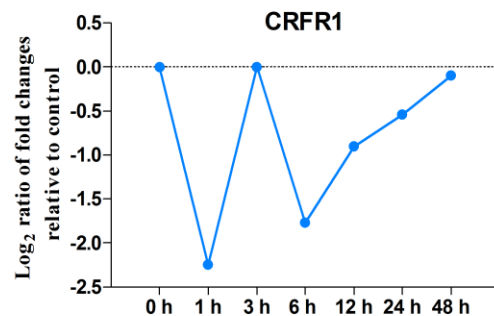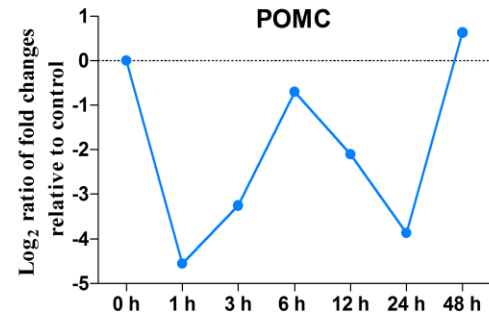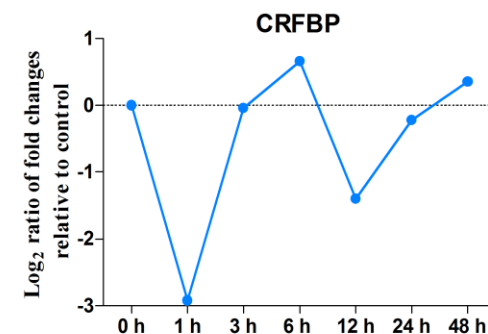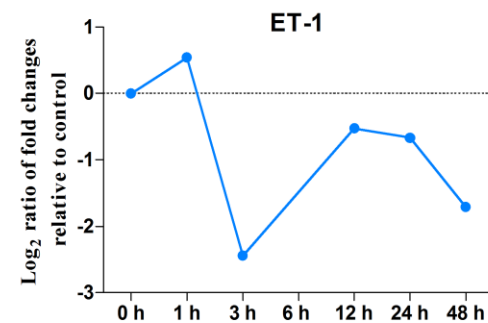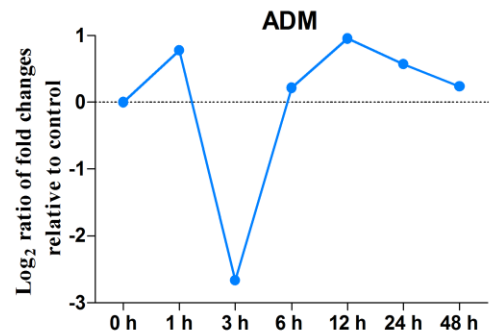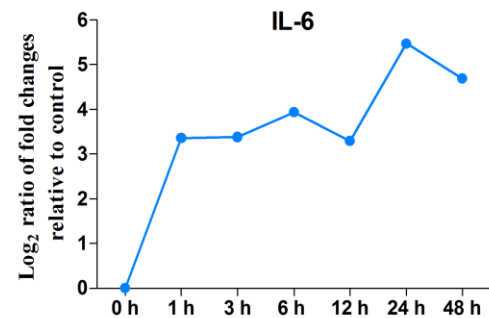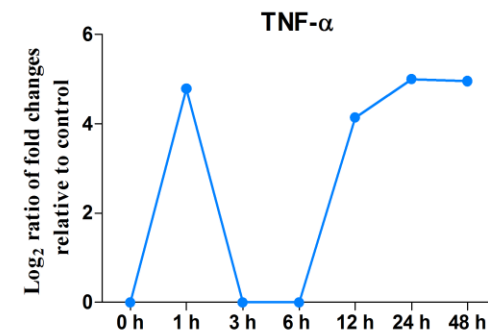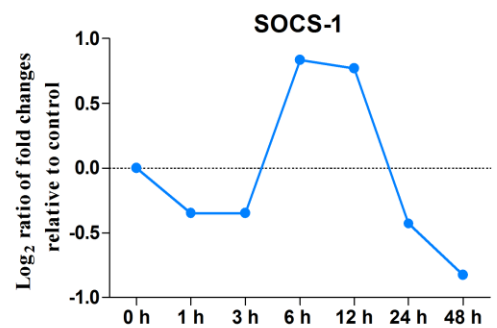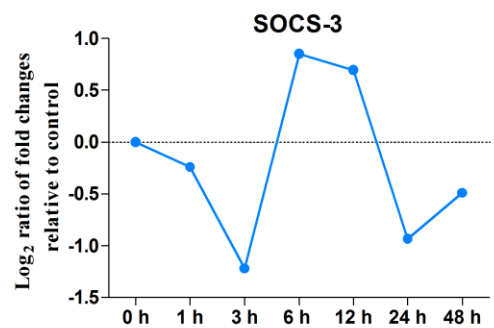

Supplement: S12 Fig — The gene expression levels were calculated based on RPKM values [69], and comparisons of gene expression difference between control (0 h) and each time point after hypoxia exposure (1, 3, 6, 12, 24, and 48 h) were performed using the method described by Audic and Claverie [77]. Genes with fold change ≥ 2 and FDR ≤ 0.001 were defined as differentially expressed genes (DEGs). Gene expression patterns were analyzed by the open source clustering software Cluster 3.0 and presented using the GraphPad Prism 5 software. The Y-axis is log2 ratio of fold changes relative to control. The key hypothalamic-pituitary-adrenal (HPA) axis-relevant genes, including corticotropin-releasing factor (CRF), CRF receptor 1 (CRFR1), pro-opiomelanocortin (POMC), and CRF-binding protein (CRFBP) displayed a down-up-down-up (W-type) dynamic expression pattern under hypoxia stress. In contrast, the endothelin-1 (ET-1) and adrenomedullin (ADM) genes showed an up-down-up-down (M-type) dynamic expression pattern, and the time of inflexion point corresponded with that of CRF, CRFR1, POMC, and CRFBP. The expression of the inflammatory cytokine genes (IL-6/TNF-α) also showed the M-type pattern and was consistent with that of ET-1/ADM. Besides, both SOCS-1 and SOCS-3 in the L. crocea brain display opposite expression patterns against IL-6 and TNF-α. (PDF) [file pgen.1005118.s012.pdf]

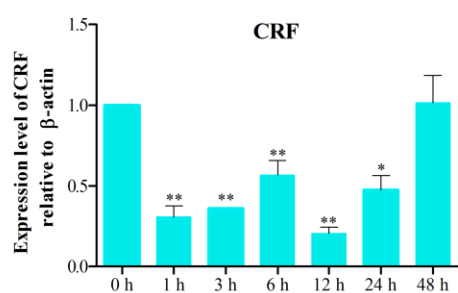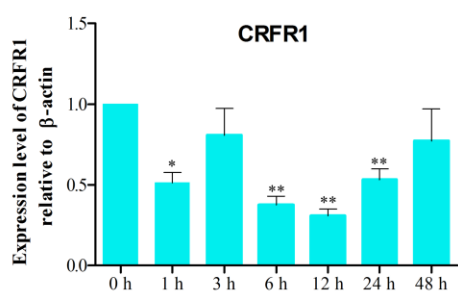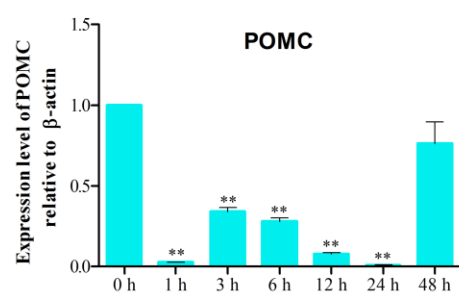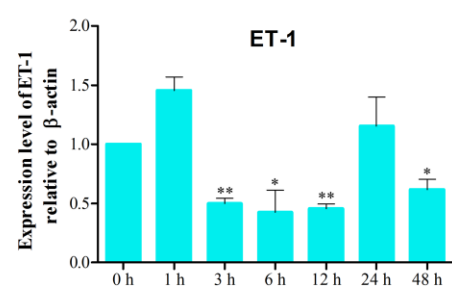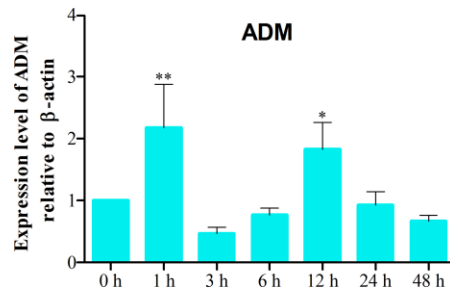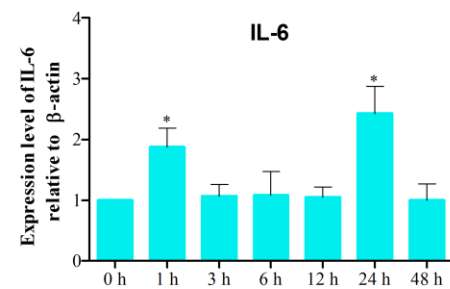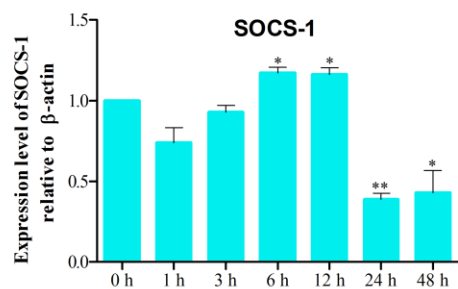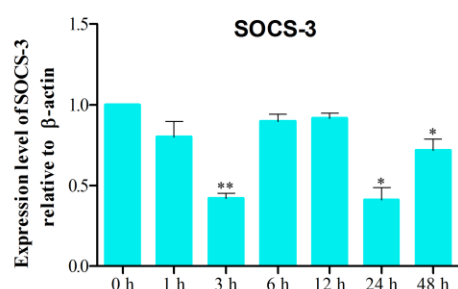

Supplement: S13 Fig — Total RNA was extracted from the brain tissues of L. crocea collected at 0, 1, 3, 6, 12, 24 and 48 h after hypoxia induction. Expression levels of selected genes involved in neuro-endocrine-immunity network (CRF, CRFR1, POMC, ET-1, ADM, IL-6, SOCS-1 and SOCS-3) in the brain tissues at each time point after hypoxia induction were detected by real-time PCR. The expression levels of each gene were expressed relative to those of β-actin in each sample using the 2-ΔΔCT method [79]. Each real-time PCR assay was repeated three times. The data of real-time PCR were expressed as the standard error of the mean (SEM). Two-tailed Student’s t test was used for the significance test of the gene expression levels in brain tissues between 0 h and each time point after hypoxia exposure. * P< 0.05, ** P< 0.01. (PDF) [file pgen.1005118.s013.pdf]

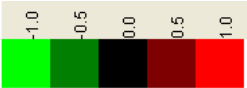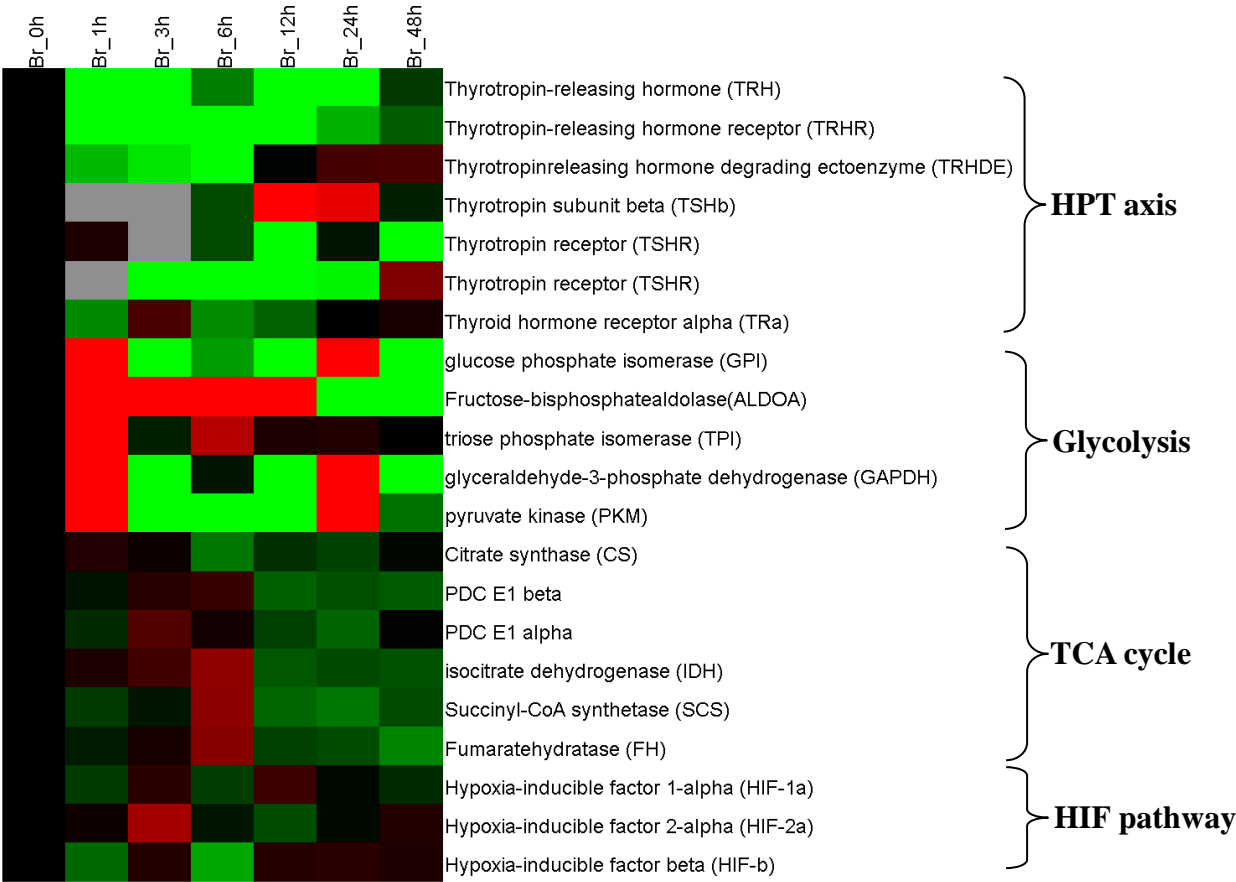

Supplement: S14 Fig — The gene expression levels were calculated based on RPKM values [69], and comparisons of gene expression difference between control (0 h) and each time point after hypoxia exposure (1, 3, 6, 12, 24, and 48 h) were performed using the method described by Audic and Claverie [77]. Genes with fold change ≥ 2 and FDR ≤ 0.001 were defined as differentially expressed genes (DEGs). Gene expression patterns were analyzed by the open source clustering software Cluster 3.0 and presented using the Java TreeView. Genes shown in red are up-regulated, and those shown in green are down-regulated, relative to the control. Genes with no expression are shown in gray. Values in toolbar are log2 ratio of fold changes relative to control. (PDF) [file pgen.1005118.s014.pdf]

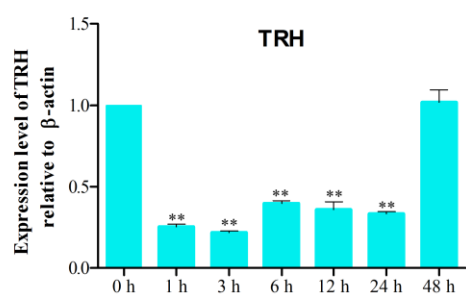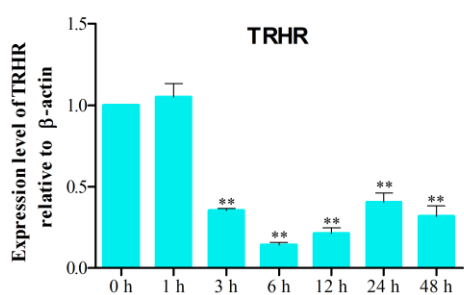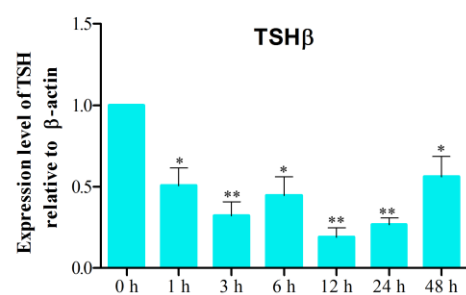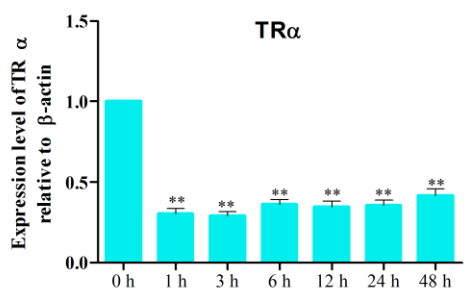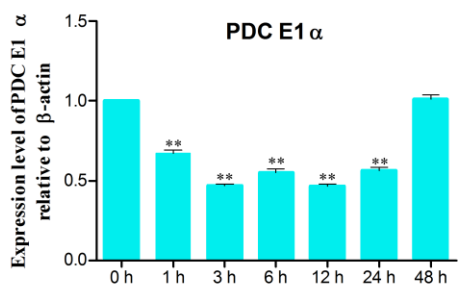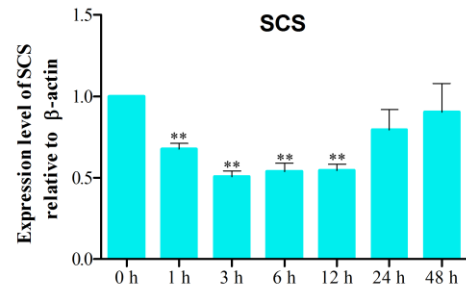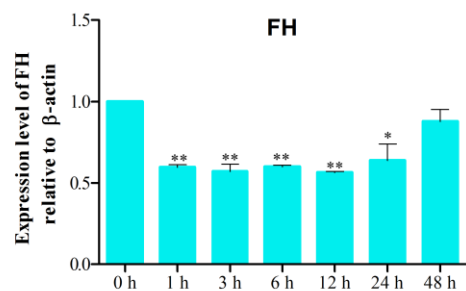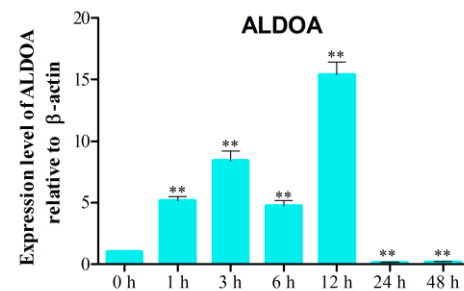

Supplement: S15 Fig — Total RNA was extracted from the brain tissues of L. crocea collected at 0, 1, 3, 6, 12, 24, and 48 h after hypoxia induction. Expression levels of selected genes involved in neuro-endocrine-metabolism network (TRH, TRHR, TSHβ, TRα, PDC E1α, SCS, FH, and ALDOA) in the brain tissues at each time point after hypoxia induction were detected by real-time PCR. The expression levels of each gene were expressed relative to those of β-actin in each sample using the 2-ΔΔCT method [79]. Each real-time PCR assay was repeated three times. The data of real-time PCR were expressed as the standard error of the mean (SEM). Two-tailed Student’s t test was used for the significance test of the gene expression levels in brain tissues between 0 h and each time point after hypoxia exposure. * P< 0.05, ** P< 0.01. (PDF) [file pgen.1005118.s015.pdf]

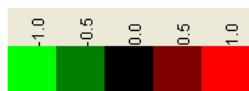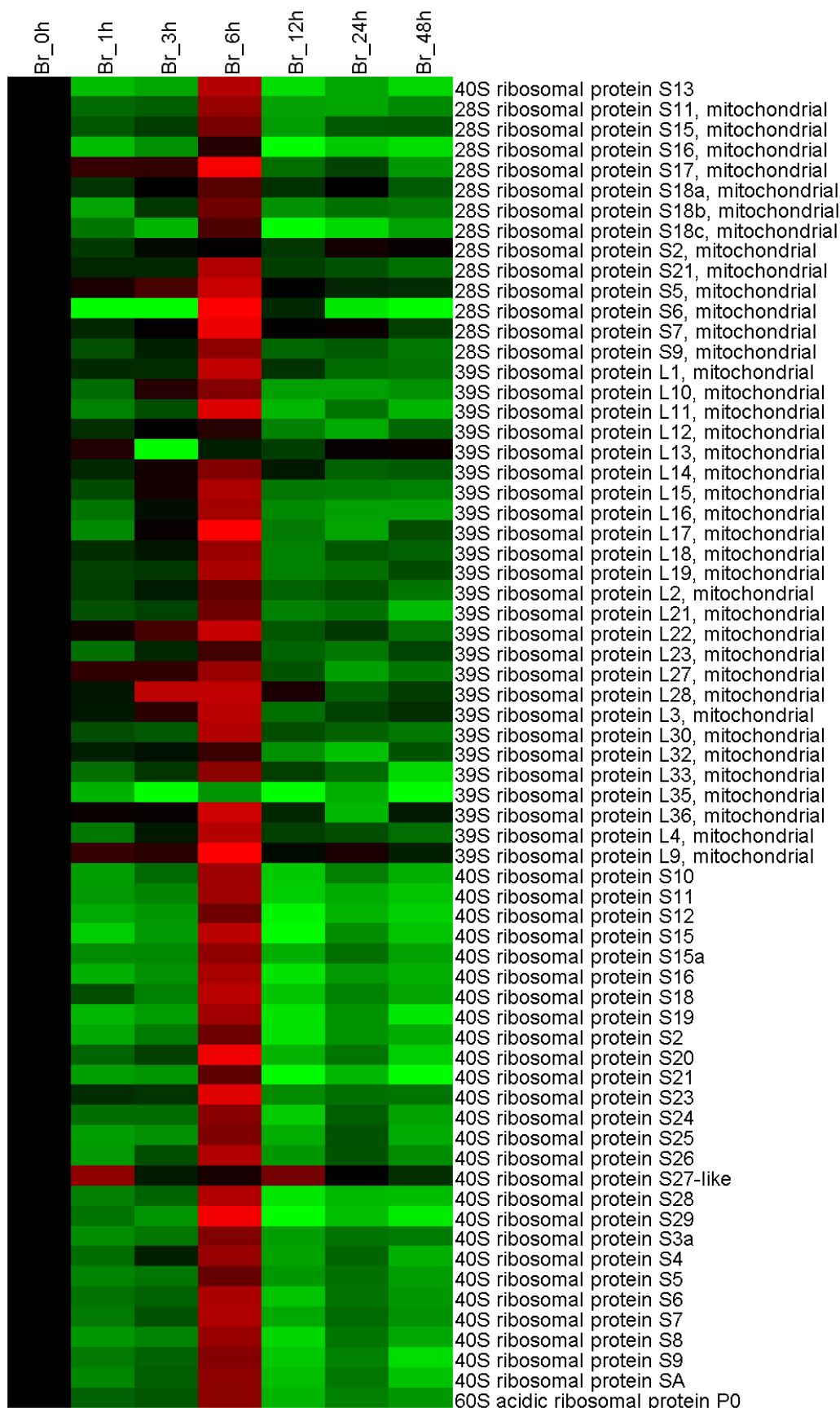

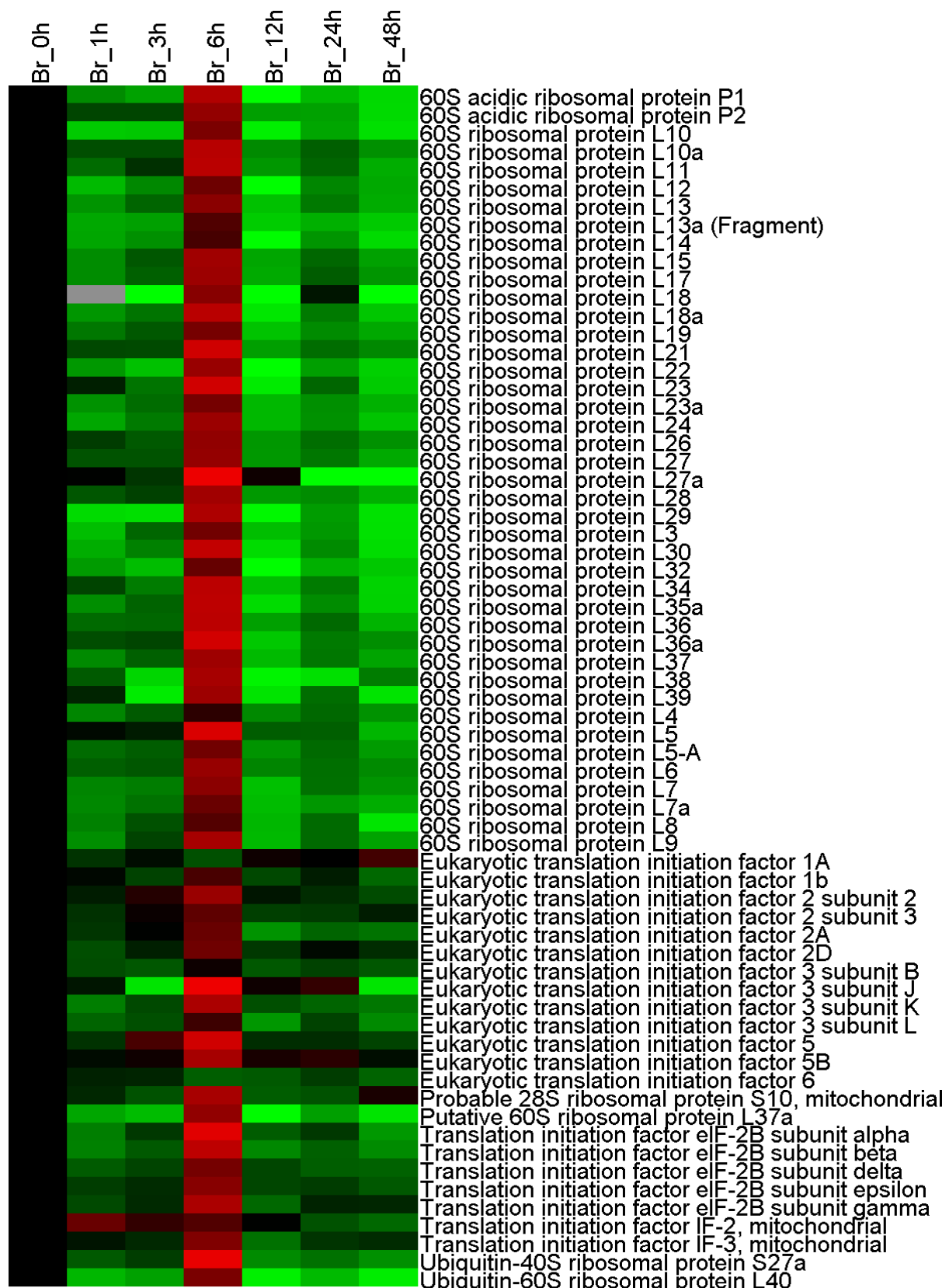

Supplement: S16 Fig — The gene expression levels were calculated based on RPKM values [69], and comparisons of gene expression difference between control (0 h) and each time point after hypoxia exposure (1, 3, 6, 12, 24, and 48 h) were performed using the method described by Audic and Claverie [77]. Genes with fold change ≥ 2 and FDR ≤ 0.001 were defined as differentially expressed genes (DEGs). Gene expression patterns were analyzed by the open source clustering software Cluster 3.0 and presented using the Java TreeView. Genes shown in red are up-regulated, and those shown in green are down-regulated, relative to the control. Genes with no expression are shown in gray. Values in toolbar are log2 ratio of fold changes relative to control. (PDF) [file pgen.1005118.s016.pdf]

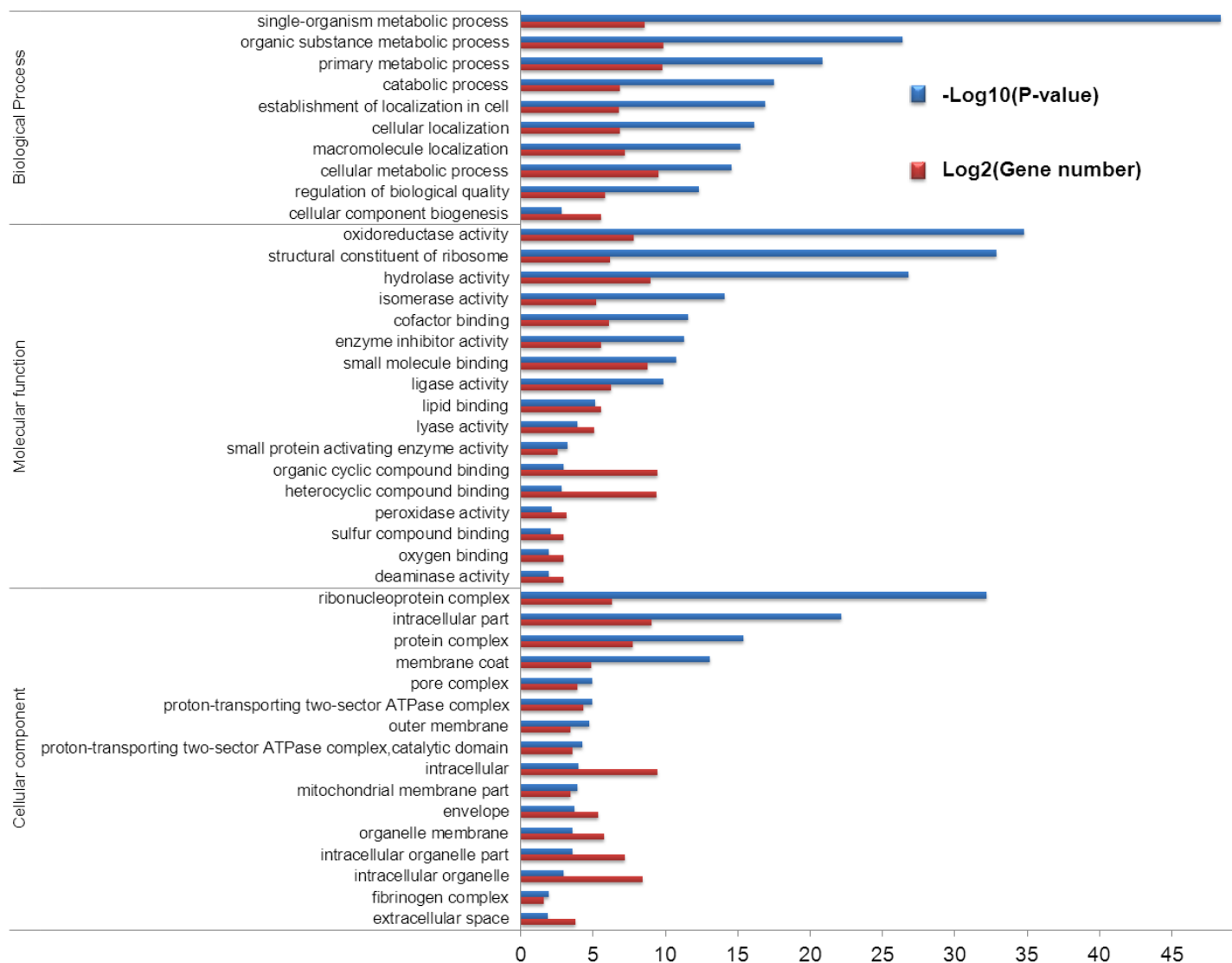

Supplement: S18 Fig — Here we applied the EnrichPipeline to extract annotation information in Gene Ontology with P<0.01. The functions are summarized in three main categories: biological process, cellular component, and molecular function. (PDF) [file pgen.1005118.s018.pdf]

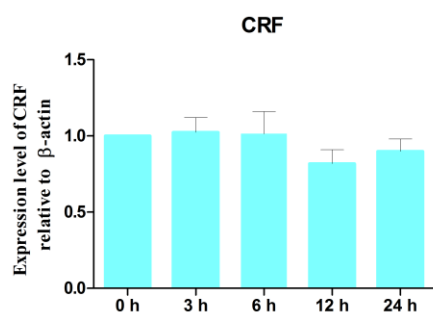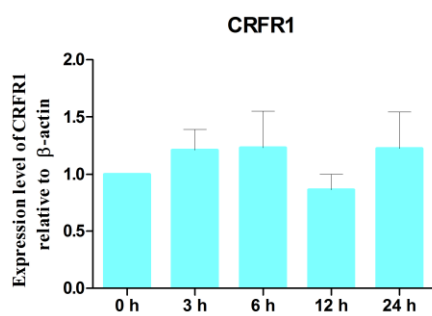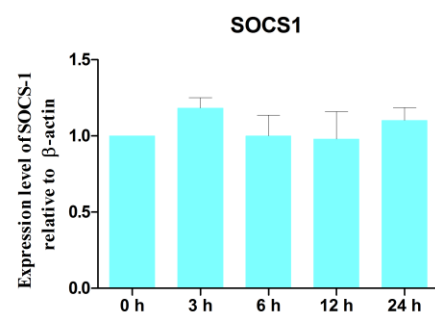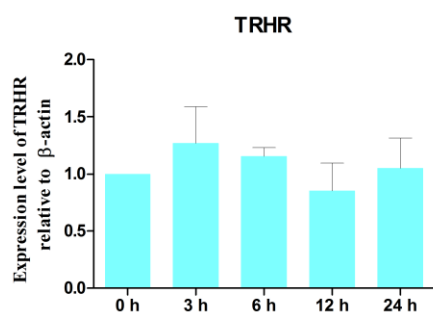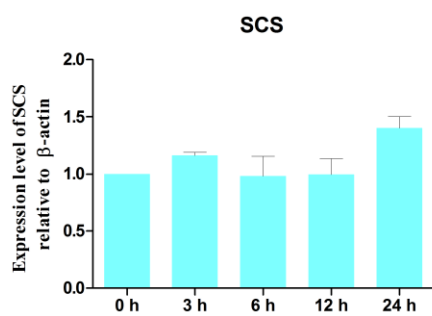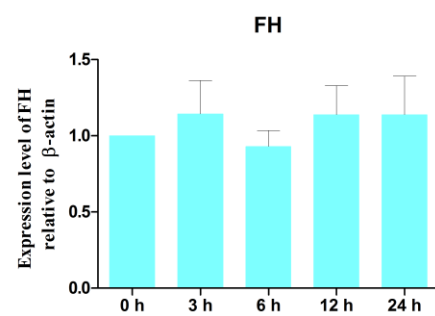

Supplement: S19 Fig — Total RNA was extracted from L. crocea brain tissues collected at 0, 3, 6, 12, and 24 h with no hypoxia treatment. Expression levels of six selected genes (CRF, CRFR1, SOCS1, TRHR, SCS, and FH), which were identified as differentially expressed genes in the brain transcriptomes of hypoxia-induced L. crocea (S12 and S14 Fig.), in the untreated brain tissues at each time point were detected by real-time PCR. The expression levels of each gene were expressed relative to those of β-actin in each sample using the 2-ΔΔCT method [79]. Each real-time PCR assay was repeated three times. The data of real-time PCR were expressed as the standard error of the mean (SEM). Two-tailed Student’s t test was used for the significance test of the gene expression levels in brain tissues between 0 h and each later time point. The results showed that the expression levels of these six genes were not significantly changed between 0 h and each later time point in brain tissues of untreated fish, suggesting that the gene expression alterations under hypoxia should not come from change in the baseline expression levels. (PDF) [file pgen.1005118.s019.pdf]
