# Supplementary material for: Genome Sequencing of the Perciform Fish Larimichthys crocea Provides Insights into Molecular and Genetic Mechanisms of Stress Adaptation
Source: PLoS Genet. 2015 Apr 2;11(4):e1005118. doi: 10.1371/journal.pgen.1005118 (PMC4383535; doi:10.1371/journal.pgen.1005118)
Supplement: S4 Fig — (PDF) [file pgen.1005118.s004.pdf]

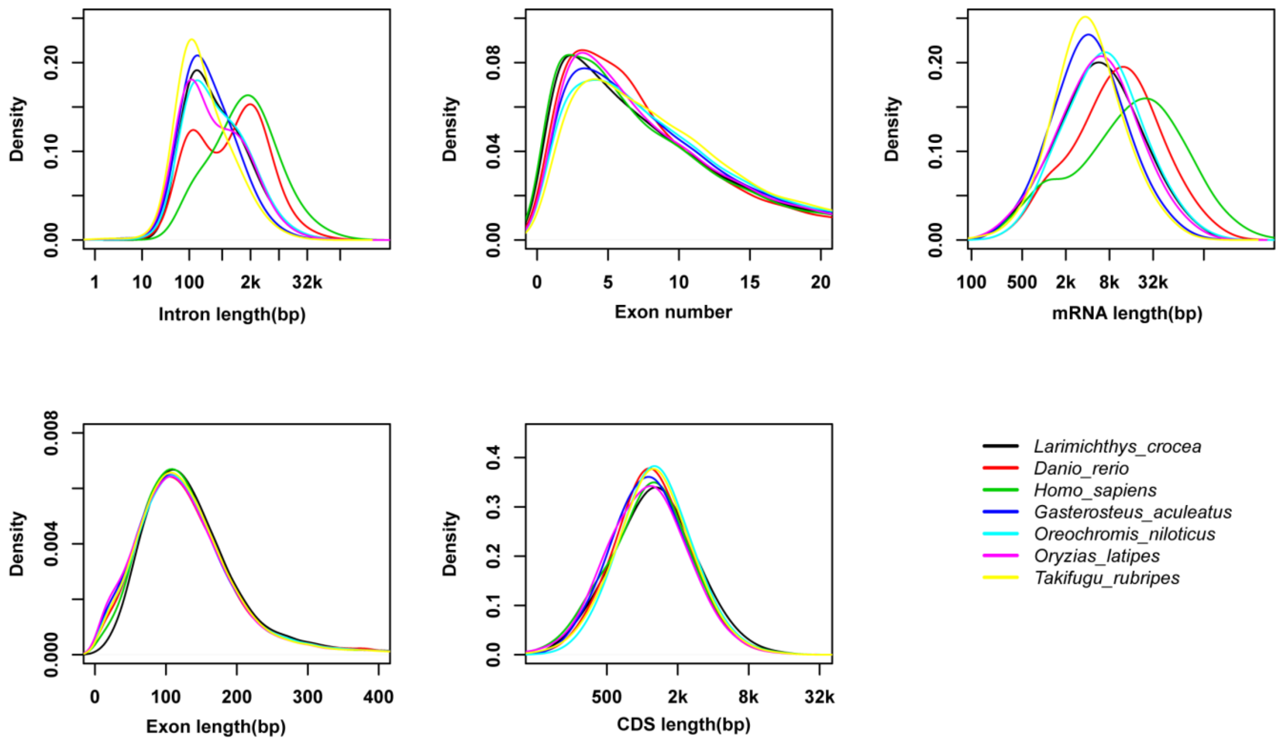
Density
0.00
0.10
0.20
1
10
100
2k
32k
Intron length(bp)
Density
0.00
0.04
0.08
0
5
10
15
20
Exon number
Density
0.00
0.10
0.20
100
500
2k
8k
32k
mRNA length(bp)
Density
0.000
0.004
0.008
0
100
200
300
400
Exon length(bp)
Density
0.0
0.1
0.2
0.3
0.4
500
2k
8k
32k
CDS length(bp)
Larimichthys_crocea
Danio_rerio
Homo_sapiens
Gasterosteus_aculeatus
Oreochromis_niloticus
Oryzias_latipes
Takifugu_rubripes
