# Supplementary material for: Genome Sequencing of the Perciform Fish Larimichthys crocea Provides Insights into Molecular and Genetic Mechanisms of Stress Adaptation
Source: PLoS Genet. 2015 Apr 2;11(4):e1005118. doi: 10.1371/journal.pgen.1005118 (PMC4383535; doi:10.1371/journal.pgen.1005118)
Supplement: S17 Fig — Distribution of GALNTs 1–14 in the genomes of human, zebrafish, stickleback, Japanese pufferfish, green spotted pufferfish, and large yellow croaker is shown. (PDF) [file pgen.1005118.s017.pdf]

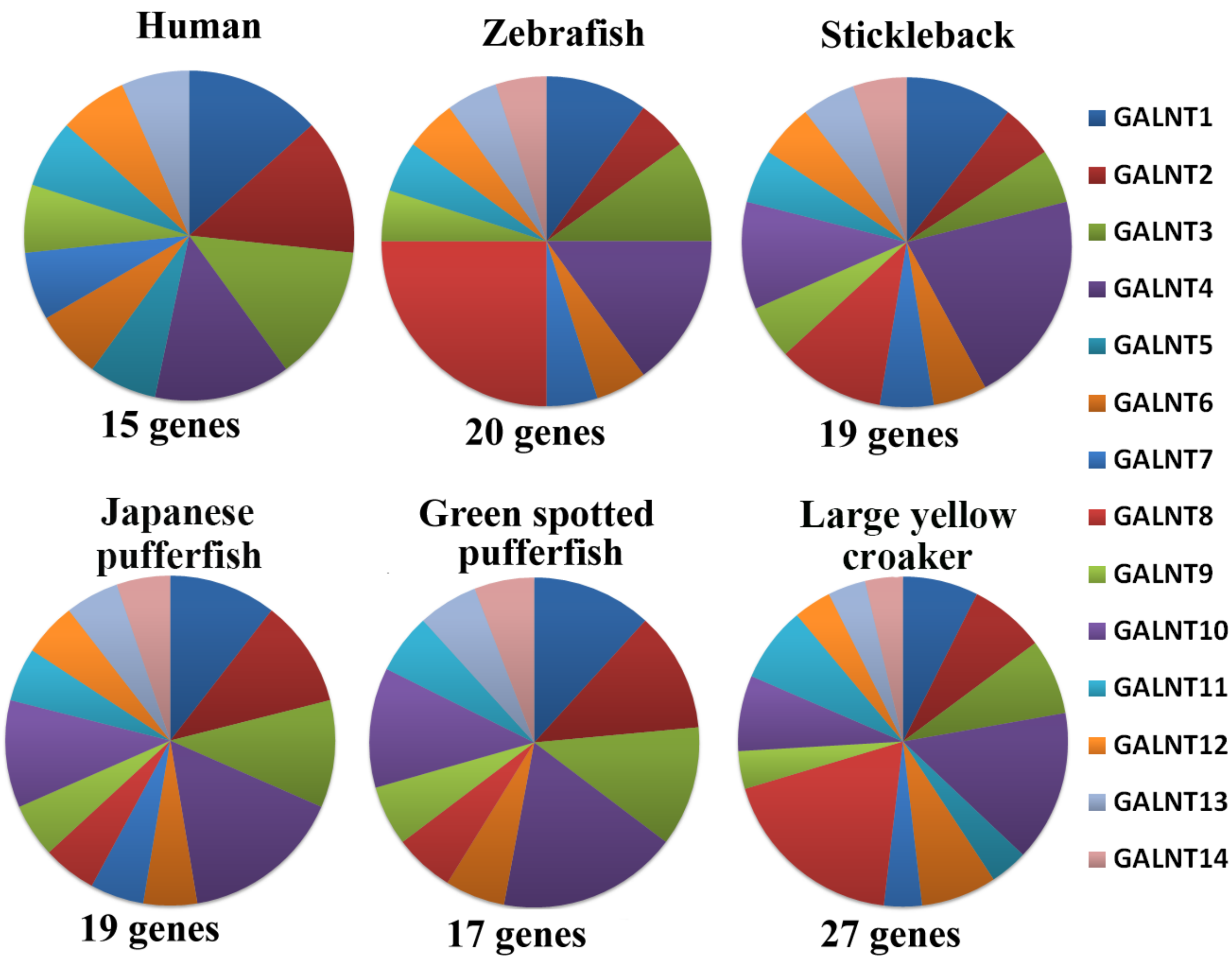
Human
15 genes
Zebrafish
20 genes
Stickleback
19 genes
Japanese pufferfish
19 genes
Green spotted pufferfish
17 genes
Large yellow croaker
27 genes
GALNT1
GALNT2
GALNT3
GALNT4
GALNT5
GALNT6
GALNT7
GALNT8
GALNT9
GALNT10
GALNT11
GALNT12
GALNT13
GALNT14
