# Supplementary material for: Genome Sequencing of the Perciform Fish Larimichthys crocea Provides Insights into Molecular and Genetic Mechanisms of Stress Adaptation
Source: PLoS Genet. 2015 Apr 2;11(4):e1005118. doi: 10.1371/journal.pgen.1005118 (PMC4383535; doi:10.1371/journal.pgen.1005118)
Supplement: S1 Table — (PDF) [file pgen.1005118.s020.pdf]

Table S1: Summary of BACs used in *L. crocea* genome project

Average Length of BAC (Kbp)	BAC Number	96-well Plates	Sequence Bases (Gbp)	Average per BAC (×)	Genome Depth (×)
120	42,528	443	324.73	63.63	464
