# Supplementary material for: Genome Sequencing of the Perciform Fish Larimichthys crocea Provides Insights into Molecular and Genetic Mechanisms of Stress Adaptation
Source: PLoS Genet. 2015 Apr 2;11(4):e1005118. doi: 10.1371/journal.pgen.1005118 (PMC4383535; doi:10.1371/journal.pgen.1005118)
Supplement: S2 Table — (PDF) [file pgen.1005118.s021.pdf]

Table S2: Summary of k-mer analysis

k-mer size	k-mer number	Peak depth	Genome size	Used base	Used read	Coverage of Genome (×)
17	30,423,075,312	44	691,433,530	36,217,946,800	362,179,468	52

Based on the k-mer analysis, the genome size of *L. crocea* is calculated to be 691 Mb.
