# Supplementary material for: Genome Sequencing of the Perciform Fish Larimichthys crocea Provides Insights into Molecular and Genetic Mechanisms of Stress Adaptation
Source: PLoS Genet. 2015 Apr 2;11(4):e1005118. doi: 10.1371/journal.pgen.1005118 (PMC4383535; doi:10.1371/journal.pgen.1005118)
Supplement: S3 Table — (PDF) [file pgen.1005118.s022.pdf]

Table S3: Stastics of BAC sequences used for mergence

	Contig		Scaffold	
	**Length (bp)**	**Number**	**Length (bp)**	**Number**
N90	1,096	563,506	3,360	150,285
N80	2,057	363,547	7,552	89,358
N70	3,299	248,223	13,256	58,373
N60	4,781	172,565	20,606	39,642
N50	6,560	118,845	29,220	26,987
N40	8,741	79,019	40,174	17,904
N30	11,514	48,980	52,841	11,232
N20	15,505	26,348	73,581	6,228
N10	22,331	9,952	100,567	2,634
Total length (bp):	3,006,049,398		3,098,436,274	
Max length (bp):	98,562		149,895	
Number (>500 bp)	893,977		388,748	
Number (>2000 bp)	371,497		197,679	

BAC sequences, which were longer than 500 bp, were merged.
