# Supplementary material for: Genome Sequencing of the Perciform Fish Larimichthys crocea Provides Insights into Molecular and Genetic Mechanisms of Stress Adaptation
Source: PLoS Genet. 2015 Apr 2;11(4):e1005118. doi: 10.1371/journal.pgen.1005118 (PMC4383535; doi:10.1371/journal.pgen.1005118)
Supplement: S4 Table — (PDF) [file pgen.1005118.s023.pdf]

Table S4: **Information of whole-genome shotgun reads**

Insert Size	Average Read Length (bp)	Total Data (Gb)	Sequence Depth (×)	Physical Depth (×)
170 bp	100	13.95	19.62	49.06
500 bp	100	22.27	31.32	26.62
2 kbp	49	18.87	26.54	541.68
5 kbp	49	4.84	6.80	346.97
10 kbp	49	6.28	8.83	901.02
20 kbp	49	3.30	4.64	946.35
40 kbp	49	0.97	1.36	556.85
Total	----	70.48	99.11	3368.55
