# Supplementary material for: Genome Sequencing of the Perciform Fish Larimichthys crocea Provides Insights into Molecular and Genetic Mechanisms of Stress Adaptation
Source: PLoS Genet. 2015 Apr 2;11(4):e1005118. doi: 10.1371/journal.pgen.1005118 (PMC4383535; doi:10.1371/journal.pgen.1005118)
Supplement: S5 Table — (PDF) [file pgen.1005118.s024.pdf]

Table S5: Statistics of final assembly

	Contig		Scaffold	
	**Length (bp)**	**Number**	**Length (bp)**	**Number**
N90	14,160	11,390	285,329	672
N80	25,118	7,939	463,062	487
N70	36,275	5,754	662,826	362
N60	48,542	4,180	856,499	272
N50	63,110	2,980	1,034,540	200
N40	79,292	2,045	1,257,841	140
N30	100,009	1,298	1,596,367	91
N20	130,560	711	1,997,749	53
N10	180,093	274	2,413,492	22
Total length (bp)	661,327,267		678,964,076	
Max length (bp)	716,891		4,914,789	
Number (>500 bp)	20,385		6,019	
Number (>1000 bp)	27,015		3,146	
Number (>2000 bp)	26,882		2,251	
