# Supplementary material for: Genome Sequencing of the Perciform Fish Larimichthys crocea Provides Insights into Molecular and Genetic Mechanisms of Stress Adaptation
Source: PLoS Genet. 2015 Apr 2;11(4):e1005118. doi: 10.1371/journal.pgen.1005118 (PMC4383535; doi:10.1371/journal.pgen.1005118)
Supplement: S6 Table — (PDF) [file pgen.1005118.s025.pdf]

Table S6: Genome assembly validation by transcripts mapping

		>0%Aligned		>%50 aligned		>%80 aligned	
**Transcripts length**	**Total transcripts**	**Number of transcripts**	**Percentage (%)**	**Number of transcripts**	**Percentage (%)**	**Number of transcripts**	**Percentage (%)**
>500 bp	39,106	38,520	98.50	38,174	97.62	37,460	95.79
>1000 bp	18,184	17,966	98.80	17,786	97.81	17,421	95.80

The male and female transcriptomes were from eleven mixed tissues respectively. Then the sequencing short reads were assemblied to transcripts. Transcripts were mapped to *L. crocea* genome by BLAT to validate completeness of genome assembly.
