# Supplementary material for: Genome Sequencing of the Perciform Fish Larimichthys crocea Provides Insights into Molecular and Genetic Mechanisms of Stress Adaptation
Source: PLoS Genet. 2015 Apr 2;11(4):e1005118. doi: 10.1371/journal.pgen.1005118 (PMC4383535; doi:10.1371/journal.pgen.1005118)
Supplement: S7 Table — (PDF) [file pgen.1005118.s026.pdf]

Table S7: **Summary of genome assembly of seven sequenced teleost species**

Species	Total length of scaffolds (bp)	Total length of contigs (bp)	Percentage of gaps	N50 of scaffolds (bp)	N50 of contigs (bp)	Number of scaffolds	Number of contigs
Larimichthys crocea	678,964,076	661,327,267	2.60%	1,034,540	63,110	6,019	27,015
Gadus morhua	832,114,588	607,869,276	26.95%	136,353	2,310	398,859	555,245
Takifugu rubripes	393,312,790	351,017,437	10.75%	858,115	49,304	7,214	33,204
Oryzias latipes	869,000,216	700,384,697	19.40%	29,908,082	9,628	7,189	134,399
Tetraodon nigroviridis	358,618,246	302,293,082	15.71%	13,390,619	30,260	27	33,235
Gasterosteus aculeatus	461,533,321	446,627,734	3.23%	18,115,788	83,204	1,842	16,966
Danio rerio	1,412,464,843	1,409,741,015	0.19%	54,093,808	1,073,451	1,133	28,972

Assemblies of six other teleost species are downloaded from Ensemble74. Since *Oryzias latipes*, *Tetraodon nigroviridis*, *Gasterosteus aculeatus*, and *Danio rerio* were anchored to chromosomes (Shadowed background), their scaffold N50 is larger than 10 Mb.
