# Supplementary material for: Genome Sequencing of the Perciform Fish Larimichthys crocea Provides Insights into Molecular and Genetic Mechanisms of Stress Adaptation
Source: PLoS Genet. 2015 Apr 2;11(4):e1005118. doi: 10.1371/journal.pgen.1005118 (PMC4383535; doi:10.1371/journal.pgen.1005118)
Supplement: S8 Table — (PDF) [file pgen.1005118.s027.pdf]

Table S8: Summary of repetitive elements in *L. crocea* genome

TE subtype	RepeatMasker		ProteinMasker		Repeatscout		Combination	
	#base	%genome	#base	%genome	#base	%genome	#base	%genome
**DNA**	19,575,778	2.88	4,206,662	0.62	25,051,324	3.69	35,335,543	5.20
**LINE**	11,874,487	1.75	10,982,979	1.62	13,995,684	2.06	19,862,941	2.93
**LTR**	9,754,813	1.44	5,727,464	0.84	8,078,576	1.19	14,945,772	2.20
**Other**	7,999	0.00	0	0.00	0	0.00	7,999	0.00
**SINE**	2,268,995	0.33	0	0.00	2,501,774	0.37	3,687,458	0.54
**Satellite**	0	0.00	0	0.00	2,755,142	0.41	2,755,142	0.41
**Simple repeat**	0	0.00	0	0.00	3,945,489	0.58	3,945,489	0.58
**Unknown**	541,527	0.08	0	0.00	41,857,750	6.16	42,374,776	6.24
**Total**	44,023,599	6.48	20,917,105	3.08	98,185,739	14.46	122,915,120	18.10
