# Supplementary material for: Genome Sequencing of the Perciform Fish Larimichthys crocea Provides Insights into Molecular and Genetic Mechanisms of Stress Adaptation
Source: PLoS Genet. 2015 Apr 2;11(4):e1005118. doi: 10.1371/journal.pgen.1005118 (PMC4383535; doi:10.1371/journal.pgen.1005118)
Supplement: S9 Table — (PDF) [file pgen.1005118.s028.pdf]

Table S9: Top ten transposable elements (TE) in seven teleost species

Fam ID	*Larimichthys crocea*	*Danio rerio*	*Gadus morhua*	*Gasterosteus aculeatus*	*Oryzias latipes*	*Takifugu rubripes*	*Tetraodon nigroviridis*
**SINE/V**	5,616	152,886	168	4,851	14,797	1,298	1,401
**SINE/MIR**	3,833	30	3,797	3,044	6,296	49	27
**DNA/DNA**	3,831	322,831	1,366	638	2,517	174	41
**DNA/TcMar**	3,630	188,595	1,131	2,564	20,945	3,708	1,395
**SINE/Mermaid**	3,115	39	71	249	7,474	3,321	374
**LTR/Gypsy**	2,580	32,588	2,119	6,135	3,027	3,698	432
**LTR/ERVK**	1,776	2,126	8,475	1,102	157	955	1,205
**DNA/hAT**	1,634	310,603	1,375	1,143	5,919	2,701	1,615
**LINE/L2**	1,266	9,323	1,068	1,148	3,237	1,281	46
**LINE/RTE**	1,001	1,458	490	549	4,743	1,188	541

TE was identified by alignment and rebased by RepeatMasker. To confirm the completeness of TE, we chose TE with length >30 bp, aligning ratio>0.2, and divergence>50% for rebasing the reference TE, respectively.
