# Supplementary material for: Genome Sequencing of the Perciform Fish Larimichthys crocea Provides Insights into Molecular and Genetic Mechanisms of Stress Adaptation
Source: PLoS Genet. 2015 Apr 2;11(4):e1005118. doi: 10.1371/journal.pgen.1005118 (PMC4383535; doi:10.1371/journal.pgen.1005118)
Supplement: S10 Table — (PDF) [file pgen.1005118.s029.pdf]

Table S10: **Comparison of repeat content from nine sequenced vertebrate species**

	Genome length (Mbp)	Repeat content from RepeatMasker (%)	Repeat content from publication (%)
Larimichthys crocea	679	6.02	18.1
Danio rerio	1,412	49.48	52.2
Gadus morhua	832	10.66	25.4
Gasterosteus aculeatus	462	7.08	25.2
Oryzias latipes	869	8.56	17.5
Takifugu rubripes	393	7.74	--
Tetraodon nigroviridis	359	4.60	--
Mus musculus	2,717	37.69	38
Homo sapiens	3,096	40.08	45

Our results showed that *L. crocea* had a relative compact genome structure.
