# Supplementary material for: Genome Sequencing of the Perciform Fish Larimichthys crocea Provides Insights into Molecular and Genetic Mechanisms of Stress Adaptation
Source: PLoS Genet. 2015 Apr 2;11(4):e1005118. doi: 10.1371/journal.pgen.1005118 (PMC4383535; doi:10.1371/journal.pgen.1005118)
Supplement: S11 Table — (PDF) [file pgen.1005118.s030.pdf]

Table S11: Statistics of predicted protein-coding genes

		Gene number	Complete ORF	%	Single Exongene	%	Average transcript length (bp)	Average CDS length (bp)	Average exons per gene	Average exon length (bp)	Average intron length (bp)
Denovo	august	30,182	30,014	99.44	3,873	12.83	10,123	1,428	7.99	179	1,243
	genescan	38,196	38,196	100.00	3,061	8.01	12,441	1,529	8.30	184	1,496
	snap	65,053	61,944	95.22	4,061	6.24	16,083	1,104	7.80	142	2,203
RNAseq	RNAseq	39,528	22	0.06	0	0.00	11,227	2,151	8.06	267	1,285
Homo	*Danio rerio*	31,003	4,245	13.69	6,750	21.77	7,870	1,369	7.18	191	1,052
	Gasterosteus aculeatus	31,883	4,727	14.83	8,478	26.59	7,417	1,224	7.03	174	1,026
	Homo sapiens	22,525	2,020	8.97	4,224	18.75	9,213	1,390	8.20	169	1,086
	Oreochromis niloticus	34,331	6,936	20.20	8,387	24.43	7,523	1,299	7.04	184	1,030
	Oryziaslatipes	34,788	4,169	11.98	9,248	26.58	6,608	1,176	6.43	183	1,001
	Takifugu rubripes	27,029	3,802	14.07	5,311	19.65	8,802	1,396	7.94	176	1,067
	Tetraodon nigroviridis	25,342	3,911	15.43	3,756	14.82	8,831	1,401	8.24	170	1,026
Glean	Glean	26,922	26,039	96.72	2,867	10.65	13,508	1,716	9.49	181	1,390
Final	Final (filt denovo genes with rpkm<1)	25,401	24,523	96.54	2,321	9.14	13,816	1,766	9.91	178	1,353
Closely related species	*Danio rerio*	25,663	19,100	74.43	1,636	6.37	24,727	1,583	9.28	170	2,794
	Gasterosteus aculeatus	20,756	8,146	39.25	1,085	5.23	8,577	1,539	10.41	148	748
	Homo sapiens	20,087	19,111	95.14	2,188	10.89	51,959	1,608	9.46	170	5,952
	Oreochromis niloticus	21,437	12,917	60.26	1,067	4.98	14,906	1,714	10.90	157	1,332
	Oryzias latipes	19,658	6,978	35.50	1,009	5.13	12,428	1,505	10.21	147	1,186
	Takifugu rubripes	18,508	5,487	29.65	643	3.47	7,719	1,651	11.05	149	604
	Tetraodon nigroviridis	19,570	6,837	34.94	777	3.97	6,191	1,512	10.52	144	492

Light green background corresponds to *Larimichthys crocea.*
