# Supplementary material for: Genome Sequencing of the Perciform Fish Larimichthys crocea Provides Insights into Molecular and Genetic Mechanisms of Stress Adaptation
Source: PLoS Genet. 2015 Apr 2;11(4):e1005118. doi: 10.1371/journal.pgen.1005118 (PMC4383535; doi:10.1371/journal.pgen.1005118)
Supplement: S12 Table — (PDF) [file pgen.1005118.s031.pdf]

Table S12: **Functional classification of *L. crocea* genes**

	Gene number	Percent
InterPro	20943	82.45%
KEGG	9951	39.18%
Swissprot	23178	91.25%
TrEMBL	24671	97.13%
Annotated	24729	97.35%
Unannotated	672	2.65%
