# Supplementary material for: Genome Sequencing of the Perciform Fish Larimichthys crocea Provides Insights into Molecular and Genetic Mechanisms of Stress Adaptation
Source: PLoS Genet. 2015 Apr 2;11(4):e1005118. doi: 10.1371/journal.pgen.1005118 (PMC4383535; doi:10.1371/journal.pgen.1005118)
Supplement: S13 Table — (PDF) [file pgen.1005118.s032.pdf]

Table S13: Summary of gene families in the genomes of nine sequenced vertebrate species by Treefam

Species	#Total genes	#Unclustered genes	#Families	#Unique families	Ave. genes per family
Larimichthys crocea	25,387	1,687	14,698	215	1.61
Danio rerio	25,618	1,218	14,378	112	1.7
Gadus morhua	20,490	567	14,024	12	1.42
Gallus gallus	16,344	2,987	11,840	99	1.13
Gasterosteus aculeats	20,738	786	14,036	16	1.42
Homo sapiens	19,959	3,253	13,215	421	1.26
Oryzias latipes	19,529	1,040	13,105	80	1.41
Takifugu rubripes	18,453	232	12,888	7	1.41
Tetraodon nigroviridis	19,520	828	13,206	49	1.42
