# Supplementary material for: Genome Sequencing of the Perciform Fish Larimichthys crocea Provides Insights into Molecular and Genetic Mechanisms of Stress Adaptation
Source: PLoS Genet. 2015 Apr 2;11(4):e1005118. doi: 10.1371/journal.pgen.1005118 (PMC4383535; doi:10.1371/journal.pgen.1005118)
Supplement: S14 Table — (PDF) [file pgen.1005118.s033.pdf]

Table S14: **Gene Ontology of expanded gene families in *L. crocea* genome**

GO_ID	GO_Term	*P*−value	Adjusted *P*−value
GO:0005911	cell-cell junction	5.10E-72	2.50E-69
GO:0044430	cytoskeletal part	2.18E-35	2.67E-33
GO:0005923	tight junction	1.45E-30	8.85E-29
GO:0046872	metal ion binding	2.15E-30	1.17E-28
GO:0003774	motor activity	1.06E-27	4.33E-26
GO:0016459	myosin complex	1.47E-23	5.53E-22
GO:0007156	homophilic cell adhesion	1.27E-22	4.45E-21
GO:0004222	metalloendopeptidase activity	4.21E-19	1.21E-17
GO:0046914	transition metal ion binding	8.55E-19	2.33E-17
GO:0005856	cytoskeleton	9.35E-18	2.41E-16
GO:0008270	zinc ion binding	6.54E-17	1.53E-15
GO:0005198	structural molecule activity	3.50E-14	6.94E-13
GO:0008237	metallopeptidase activity	3.54E-14	6.94E-13
GO:0044459	plasma membrane part	1.75E-13	2.96E-12
GO:0004499	N,N-dimethylanilinemonooxygenase activity	1.34E-11	1.99E-10
GO:0016165	lipoxygenase activity	2.96E-11	4.15E-10
GO:0004930	G-protein coupled receptor activity	1.95E-10	2.46E-09
GO:0005833	hemoglobin complex	8.04E-10	9.39E-09
GO:0030246	carbohydrate binding	8.53E-10	9.59E-09
GO:0016702	oxidoreductase activity, acting on single donors with incorporation of molecular oxygen, incorporation of two atoms of oxygen	1.98E-09	2.07E-08
GO:0006691	leukotriene metabolic process	5.05E-09	4.67E-08
GO:0015671	oxygen transport	5.75E-09	5.12E-08
GO:0019825	oxygen binding	1.01E-08	8.55E-08
GO:0005529	sugar binding	1.37E-07	1.07E-06
GO:0003956	NAD(P)+-protein-arginine ADP-ribosyltransferase activity	1.83E-07	1.40E-06
GO:0043234	protein complex	3.36E-07	2.42E-06
GO:0005488	binding	4.42E-07	3.14E-06
GO:0006915	apoptotic process	5.81E-07	3.95E-06
GO:0005509	calcium ion binding	7.51E-07	5.04E-06
GO:0004668	protein-arginine deiminase activity	9.35E-07	5.87E-06
GO:0050661	NADP binding	1.05E-06	6.54E-06
GO:0042981	regulation of apoptotic process	2.48E-06	1.43E-05
GO:0004888	transmembrane signaling receptor activity	2.82E-06	1.52E-05
GO:0018101	peptidyl-citrulline biosynthetic process from peptidyl-arginine	3.70E-06	1.89E-05
GO:0016503	pheromone receptor activity	3.70E-06	1.89E-05
GO:0043232	intracellular non-membrane-bounded organelle	1.37E-05	6.03E-05

GO:0005874	microtubule	1.88E-05	8.16E-05
GO:0005922	connexon complex	2.99E-05	1.26E-04
GO:0051258	protein polymerization	7.04E-05	2.83E-04
GO:0004869	cysteine-type endopeptidase inhibitor activity	8.32E-05	3.31E-04
GO:0007155	cell adhesion	8.65E-05	3.37E-04
GO:0016042	lipid catabolic process	8.79E-05	3.39E-04
GO:0000228	nuclear chromosome	9.89E-05	3.76E-04
GO:0044464	cell part	1.22E-04	4.51E-04
GO:0044446	intracellular organelle part	1.65E-04	5.90E-04
GO:0004872	receptor activity	1.70E-04	5.99E-04
GO:0017111	nucleoside-triphosphatase activity	2.50E-04	8.65E-04
GO:0006471	protein ADP-ribosylation	2.88E-04	9.76E-04
GO:0005525	GTP binding	5.33E-04	1.71E-03
GO:0009395	phospholipid catabolic process	6.28E-04	1.97E-03
GO:0071822	protein complex subunit organization	7.31E-04	2.24E-03
GO:0050660	flavin adenine dinucleotide binding	7.93E-04	2.38E-03
GO:0007018	microtubule-based movement	1.37E-03	4.01E-03
GO:0005506	iron ion binding	1.53E-03	4.44E-03
GO:0004623	phospholipase A2 activity	1.92E-03	5.45E-03
GO:0008378	galactosyltransferase activity	2.03E-03	5.70E-03
GO:0032991	macromolecular complex	2.96E-03	8.16E-03
GO:0007017	microtubule-based process	3.11E-03	8.52E-03
GO:0004952	dopamine receptor activity	3.42E-03	9.25E-03
GO:0008146	sulfotransferase activity	3.44E-03	9.25E-03
GO:0004620	phospholipase activity	3.67E-03	9.72E-03
GO:0004175	endopeptidase activity	5.46E-03	1.40E-02
GO:0007050	cell cycle arrest	5.57E-03	1.41E-02
GO:0004683	calmodulin-dependent protein kinase activity	8.04E-03	2.00E-02
GO:0005272	sodium channel activity	8.45E-03	2.09E-02
GO:0007049	cell cycle	9.74E-03	2.39E-02
GO:0007186	G-protein coupled receptor signaling pathway	9.86E-03	2.39E-02
GO:0044255	cellular lipid metabolic process	1.01E-02	2.43E-02
GO:0044425	membrane part	1.02E-02	2.44E-02
GO:0045028	G-protein coupled purinergic nucleotide receptor activity	1.07E-02	2.53E-02
GO:0008158	hedgehog receptor activity	1.38E-02	3.22E-02
GO:0005515	protein binding	1.70E-02	3.94E-02
