# Supplementary material for: Genome Sequencing of the Perciform Fish Larimichthys crocea Provides Insights into Molecular and Genetic Mechanisms of Stress Adaptation
Source: PLoS Genet. 2015 Apr 2;11(4):e1005118. doi: 10.1371/journal.pgen.1005118 (PMC4383535; doi:10.1371/journal.pgen.1005118)
Supplement: S15 Table — (PDF) [file pgen.1005118.s034.pdf]

Table S15: **Gene Ontology of contracted gene families in *L. crocea* genome**

GO ID	GO Term	*P*−value	Adjusted *P*−value
GO:0000786	nucleosome	2.78E-15	1.29E-13
GO:0006334	nucleosome assembly	5.48E-15	1.29E-13
GO:0034622	cellular macromolecular complex assembly	1.03E-14	2.16E-13
GO:0007186	G-protein coupled receptor signaling pathway	8.69E-10	6.32E-09
GO:0004984	olfactory receptor activity	1.85E-09	1.25E-08
GO:0043232	intracellular non-membrane-bounded organelle	7.19E-08	4.12E-07
GO:0044446	intracellular organelle part	4.06E-07	2.19E-06
GO:0008417	fucosyltransferase activity	5.19E-07	2.72E-06
GO:0032991	macromolecular complex	3.21E-06	1.64E-05
GO:0009987	cellular process	6.83E-06	3.40E-05
GO:0016021	integral to membrane	1.39E-05	6.55E-05
GO:0043231	intracellular membrane-bounded organelle	4.22E-05	1.85E-04
GO:0003677	DNA binding	5.52E-05	2.32E-04
GO:0005634	nucleus	6.43E-05	2.64E-04
GO:0044424	intracellular part	1.47E-04	5.44E-04
GO:0043229	intracellular organelle	1.53E-04	5.46E-04
GO:0090304	nucleic acid metabolic process	2.05E-04	7.06E-04
GO:0044260	cellular macromolecule metabolic process	3.21E-04	1.08E-03
GO:0006486	protein glycosylation	8.44E-04	2.70E-03
GO:0043170	macromolecule metabolic process	9.73E-04	2.97E-03
GO:0016020	membrane	1.09E-03	3.27E-03
GO:0050794	regulation of cellular process	3.01E-03	8.35E-03
GO:0005783	endoplasmic reticulum	1.17E-02	2.88E-02
GO:0035014	phosphatidylinositol 3-kinase regulator activity	1.50E-02	3.60E-02
GO:0044238	primary metabolic process	1.69E-02	4.00E-02
