# Supplementary material for: Genome Sequencing of the Perciform Fish Larimichthys crocea Provides Insights into Molecular and Genetic Mechanisms of Stress Adaptation
Source: PLoS Genet. 2015 Apr 2;11(4):e1005118. doi: 10.1371/journal.pgen.1005118 (PMC4383535; doi:10.1371/journal.pgen.1005118)
Supplement: S16 Table — (PDF) [file pgen.1005118.s035.pdf]

Table S16: **Positive selection genes in *L. crocea* genome**

Gene_ID	Function
Lcro_GLEAN_10004649	40S ribosomal protein S18
Lcro_GLEAN_10020306	55 kDa erythrocyte membrane protein
Lcro_GLEAN_10021617	6-phosphofructokinase type C
Lcro_GLEAN_10020843	A disintegrin and metalloproteinase with thrombospondin motifs 6
Lcro_GLEAN_10017210	Actin-related protein 2/3 complex subunit 5
Lcro_GLEAN_10008076	Actin-related protein 6
Lcro_GLEAN_10011849	Adenosylhomocysteinase B
Lcro_GLEAN_10018601	Adenylosuccinatesynthetaseisozyme 2
Lcro_GLEAN_10010489	Alcohol dehydrogenase class-3
Lcro_GLEAN_10007833	Ankyrin repeat and SOCS box protein 7
Lcro_GLEAN_10014551	AP-1 complex subunit mu-1
Lcro_GLEAN_10013443	Arginine-glutamic acid dipeptide repeats protein
Lcro_GLEAN_10002004	ATP-dependent RNA helicase DDX19B
Lcro_GLEAN_10013668	Beta-chimaerin
Lcro_GLEAN_10015172	Calcium-dependent secretion activator 1
Lcro_GLEAN_10011303	cAMP and cAMP-inhibited cGMP 3',5'-cyclic phosphodiesterase 10A
Lcro_GLEAN_10022363	Casein kinase I isoform gamma-1
Lcro_GLEAN_10013367	CDK5 and ABL1 enzyme substrate 1
Lcro_GLEAN_10017766	Coiled-coil domain-containing protein 58
Lcro_GLEAN_10018252	Collagen alpha-3(IX) chain
Lcro_GLEAN_10002872	Deoxyhypusine synthase
Lcro_GLEAN_10026530	Dual specificity protein kinase CLK2
Lcro_GLEAN_10019946	E3 ubiquitin-protein ligase SMURF1
Lcro_GLEAN_10014912	Endoplasmic reticulum-Golgi intermediate compartment protein 3
Lcro_GLEAN_10015138	Ephrin type-A receptor 3
Lcro_GLEAN_10005594	Exocyst complex component 4
Lcro_GLEAN_10022003	F-actin-capping protein subunit beta isoforms 1 and 2
Lcro_GLEAN_10001581	F-box only protein 32
Lcro_GLEAN_10005953	G1/S-specific cyclin-D2
Lcro_GLEAN_10009848	Gamma-aminobutyric acid receptor subunit rho-3
Lcro_GLEAN_10019755	Gamma-tubulin complex component 5
Lcro_GLEAN_10008964	Gap junction beta-1 protein
Lcro_GLEAN_10010879	Glycine amidinotransferase, mitochondrial
Lcro_GLEAN_10022207	Glycylpeptide N-tetradecanoyltransferase 2
Lcro_GLEAN_10025336	Insulin-like growth factor 2 mRNA-binding protein 3
Lcro_GLEAN_10002040	Large neutral amino acids transporter small subunit 2
Lcro_GLEAN_10020640	Liprin-alpha-2
Lcro_GLEAN_10018775	Mediator of RNA polymerase II transcription subunit 14
Lcro_GLEAN_10020068	Membrane-bound transcription factor site-1 protease
Lcro_GLEAN_10003950	Metallophosphoesterase MPPED2
Lcro_GLEAN_10003121	Mitochondrial fission 1 protein

Lcro_GLEAN_10020274	Mothers against decapentaplegic homolog 1
Lcro_GLEAN_10025732	Multidrug resistance-associated protein 1
Lcro_GLEAN_10017264	Myosin light chain 1, skeletal muscle isoform
Lcro_GLEAN_10002126	Myotubularin-related protein 9
Lcro_GLEAN_10017786	N-alpha-acetyltransferase 50
Lcro_GLEAN_10011312	Netrin receptor UNC5B
Lcro_GLEAN_10021316	Neuronal membrane glycoprotein M6-b
Lcro_GLEAN_10018924	Nuclear pore complex protein Nup93
Lcro_GLEAN_10015268	Ornithine decarboxylase antizyme 1
Lcro_GLEAN_10020960	PAB-dependent poly(A)-specific ribonuclease subunit 2
Lcro_GLEAN_10026833	Paired box protein Pax-3
Lcro_GLEAN_10010560	PHD finger protein 10
Lcro_GLEAN_10017832	Phosphatidylinositol 3,4,5-trisphosphate-dependent Rac exchanger 2 protein
Lcro_GLEAN_10004465	Pre-mRNA-splicing factor CWC22 homolog
Lcro_GLEAN_10025250	Protein FAM49A
Lcro_GLEAN_10012664	Protein Wnt-2
Lcro_GLEAN_10018964	Protein Wnt-3a
Lcro_GLEAN_10002360	Ras-related protein Rab-4B
Lcro_GLEAN_10015992	RNA polymerase II subunit A C-terminal domain phosphatase SSU72
Lcro_GLEAN_10002022	rRNA 2'-O-methyltransferase fibrillarin
Lcro_GLEAN_10021731	Septin-2
Lcro_GLEAN_10024609	Serine/threonine-protein kinase A-Raf
Lcro_GLEAN_10026438	Serine/threonine-protein kinase B-raf
Lcro_GLEAN_10002810	Serine/threonine-protein phosphatase 2A 56 kDa regulatory subunit epsilon isoform
Lcro_GLEAN_10019423	Small G protein signaling modulator 3
Lcro_GLEAN_10015044	SNW domain-containing protein 1
Lcro_GLEAN_10009105	Sodium/potassium/calcium exchanger 4
Lcro_GLEAN_10025238	Sodium/potassium-transporting ATPase subunit beta-1-interacting protein 4
Lcro_GLEAN_10001011	Sphingosine 1-phosphate receptor 1
Lcro_GLEAN_10013245	Spindlin-1
Lcro_GLEAN_10014414	Stathmin-4
Lcro_GLEAN_10019921	Sterol-4-alpha-carboxylate 3-dehydrogenase, decarboxylating
Lcro_GLEAN_10020234	Syntaxin-binding protein 1
Lcro_GLEAN_10016560	Syntaxin-binding protein 5-like
Lcro_GLEAN_10009242	T-complex protein 1 subunit delta
Lcro_GLEAN_10018109	Tetraspanin-18
Lcro_GLEAN_10010087	Tetratricopeptide repeat protein 13
Lcro_GLEAN_10015907	Thrombospondin-2
Lcro_GLEAN_10024405	Transcription elongation factor 1 homolog
Lcro_GLEAN_10016129	Ubiquitin-conjugating enzyme E2 W
Lcro_GLEAN_10017384	Ubiquitin-like modifier-activating enzyme 1
Lcro_GLEAN_10021238	Ubiquitin-protein ligase E3C
Lcro_GLEAN_10015446	Unconventional myosin-VI

Lcro_GLEAN_10009254	UTP--glucose-1-phosphate uridylyltransferase
Lcro_GLEAN_10025133	Vam6/Vps39-like protein
Lcro_GLEAN_10006179	Vesicular glutamate transporter 1
Lcro_GLEAN_10025438	WD repeat and FYVE domain-containing protein 3
Lcro_GLEAN_10021755	WD repeat-containing protein mio
Lcro_GLEAN_10020092	Zinc finger protein 319
Lcro_GLEAN_10005441	Zinc finger protein 536
Lcro_GLEAN_10001014	Zinc transporter 7
