# Supplementary material for: Genome Sequencing of the Perciform Fish Larimichthys crocea Provides Insights into Molecular and Genetic Mechanisms of Stress Adaptation
Source: PLoS Genet. 2015 Apr 2;11(4):e1005118. doi: 10.1371/journal.pgen.1005118 (PMC4383535; doi:10.1371/journal.pgen.1005118)
Supplement: S17 Table — (PDF) [file pgen.1005118.s036.pdf]

Table S17: Copy number of vision-related genes in seven sequenced teleost species

Gene name	*Larimichthys crocea*	*Danio rerio*	*Gadus morhua*	*Gasterosteus aculeatus*	*Oryzias latipes*	*Takifugu rubripes*	*Tetraodon nigroviridis*
*crygm2b**	12	3	6	7	8	4	4
*cryba1**	5	4	4	4	3	3	2
*crybb3**	3	1	2	2	2	2	2
rdh12	20	18	11	14	12	11	11
arl6	27	17	15	21	19	22	17
slc17a6b	9	7	5	5	7	7	4
unc119b	7	4	3	3	4	3	3

Genes are abbreviated as *crygm2b*: crystallin gamma M2b; *cryba1*: crystallin beta A1; *crybb3*: crystallin beta B3; *rdh12*: Retinol dehydrogenase 12; *arl6*: ADP-ribosylation factor-like 6; *slc17a6b*: solute carrier family 17, member 6b; *unc119b*: unc-119 homolog B.

*Several crystallin genes (*crygm2b*, *cryba1*, and *crybb3*), which encode proteins that maintain the transparency and refractive index of the lens, were markedly expanded in the genome of *L. crocea* relative to those of other examined teleosts. The specific expansion of these crystallin genes may be helpful for improving photosensitivity by increasing lens transparency, thereby enabling the fish to easily find food and avoid predation underwater.
