# Supplementary material for: Genome Sequencing of the Perciform Fish Larimichthys crocea Provides Insights into Molecular and Genetic Mechanisms of Stress Adaptation
Source: PLoS Genet. 2015 Apr 2;11(4):e1005118. doi: 10.1371/journal.pgen.1005118 (PMC4383535; doi:10.1371/journal.pgen.1005118)
Supplement: S18 Table — (PDF) [file pgen.1005118.s037.pdf]

Table S18: Olfactory receptor-like gene repertoire in seven sequenced teleost species

	Air		Water				Air/Water	Total
Species name	alpha	gamma	delta	epsilon	zeta	eta	beta	
Larimichthys crocea	0	0	66	4	10	30	2	112
Gadus morhua	0	1	65	3	17	10	1	97
Danio rerio	0	1	69	13	36	26	7	152
Gasterosteus aculeatus	0	3	80	4	18	3	1	109
Oryzias latip	0	0	39	4	10	14	3	70
Takifugu rubripes	0	0	41	2	4	6	1	54
Tetraodon nigroviridis	0	0	33	2	2	6	1	44

A potential functional gene is a sequence that does not contain nonsense or frame shift mutation, which was re-checked by BLAST searches against the Swissprot database. Only those proteins that gave an 'Olfactory receptor' hit and with greater than 270 amino acids in length were retained and defined as functional olfactory receptor-like genes.

L. crocea possessed the highest number of genes that were classified into the "eta" group (30, $P < 0.001$), and these genes may contribute to the olfactory detection abilities, which could be useful for feeding and migration.
