# Supplementary material for: Genome Sequencing of the Perciform Fish Larimichthys crocea Provides Insights into Molecular and Genetic Mechanisms of Stress Adaptation
Source: PLoS Genet. 2015 Apr 2;11(4):e1005118. doi: 10.1371/journal.pgen.1005118 (PMC4383535; doi:10.1371/journal.pgen.1005118)
Supplement: S19 Table — (PDF) [file pgen.1005118.s038.pdf]

Table S19: Copy number of auditory sense-related genes in seven sequenced teleost species

Gene name	*Larimichthys crocea*	*Danio rerio*	*Gadus morhua*	*Gasterosteus aculeatus*	*Oryzias latip*	*Takifugu rubripes*	*Tetraodon nigroviridis*
OTOF	5	3	1	1	1	2	0
claudinj	24	24	15	13	18	15	15
OTOL1	11	7	5	6	8	8	5

For good communication, fish have developed high sensitivities to environmental sound. Three important auditory genes, *OTOF*, *claudinj*, and *OTOL1*, were significantly expanded in the *L. crocea* genome ($P < 0.01$). These expansions may contribute to the detection of sound signaling during communication, and thus to reproduction and survival. Genes are abbreviated as *OTOF*: otoferlin; *OTOL1*: otolin-1.

One more round of genome duplication occurred in *Danio rerio* relative to other fish species.
