# Supplementary material for: Genome Sequencing of the Perciform Fish Larimichthys crocea Provides Insights into Molecular and Genetic Mechanisms of Stress Adaptation
Source: PLoS Genet. 2015 Apr 2;11(4):e1005118. doi: 10.1371/journal.pgen.1005118 (PMC4383535; doi:10.1371/journal.pgen.1005118)
Supplement: S20 Table — (PDF) [file pgen.1005118.s039.pdf]

Table S20: **Comparsion of the genes encoding for selenoproteins between *L. crocea* and other sequenced vertebrate species**

Species	*Larimichthys crocea*	*Oryzias latip*	*Takifugu rubripes*	*Danio rerio*	*Gasterosteus aculeatus*	*Tetraodon nigroviridis*	*Homo sapiens*	*Mus musculus*
**Gene name**	*DI1*	*DI1*	*DI1*	*DI1*	*DI1*	*DI1*	*DI1*	*DI1*
	DI2	*DI2*	*DI2*	*DI2*	*DI2*	*DI2*	*DI2*	*DI2*
	DI3	*DI3*	*DI3*	*DI3*	*DI3*	*DI3*	*DI3*	*DI3*
	DI3b	*DI3b*	*DI3b*	*DI3b*	*DI3b*	*DI3b*		
	GPx1a	*GPx1*	*GPx1*	*GPx1*	*GPx1*	*GPx1*	*GPx1*	*GPx1*
	GPx1b		*GPx1b*	*GPx1b*	*GPx1b*	*GPx1b*		
	GPx1b2							
		GPx2	*GPx2*	*GPx2*	*GPx2*	*GPx2*	*GPx2*	*GPx2*
	GPx3	*GPx3*	*GPx3*	*GPx3*	*GPx3*	*GPx3*	*GPx3*	*GPx3*
		GPx3b	*GPx3b*	*GPx3b*	*GPx3b*	*GPx3b*		
	GPx4a	*GPx4*	*GPx4*	*GPx4*	*GPx4*	*GPx4*	*GPx4*	*GPx4*
	GPx4b	*GPx4b*	*GPx4b*	*GPx4b*	*GPx4b*	*GPx4b*		
	GPx4b2							
	GPx6						*GPx6*	
		Fep15	*Fep15*	*Fep15*	*Fep15*	*Fep15*		
	Sel15	*Sel15*	*Sel15*	*Sel15*	*Sel15*	*Sel15*	*Sel15*	*Sel15*
	SelL	*SelL*	*SelL*	*SelL*	*SelL*	*SelL*		
	SelH	*SelH*	*SelH*	*SelH*	*SelH*	*SelH*	*SelH*	*SelH*
	SelJ1	*SelJ*	*SelJ*	*SelJ*	*SelJ*	*SelJ*		
	SelJ2	*SelJ2*			*SelJ2*			
	SelI	*SelI*	*SelI*	*SelI*	*SelI*	*SelI*	*SelI*	*SelI*
	SelK	*SelK*	*SelK*	*SelK*	*SelK*	*SelK*	*SelK*	*SelK*
	SelM	*SelM*	*SelM*	*SelM*	*SelM*	*SelM*	*SelM*	*SelM*
	SelN	*SelN*	*SelN*	*SelN*	*SelN*	*SelN*	*SelN*	*SelN*
	SelO1	*SelO*	*SelO*	*SelO*	*SelO*	*SelO*	*SelO*	*SelO*
	SelO2			*SelO2*				
	SelP	*SelP*	*SelP*	*SelP*	*SelP*	*SelP*	*SelP*	*SelP*
	SelPb	*SelPb*	*SelPb*	*SelPb*	*SelPb*	*SelPb*		
	MsrB1a	*MsrB1*	*MsrB1*	*MsrB1*	*MsrB1*	*MsrB1*	*MsrB1*	*MsrB1*
	MsrB1b	*MsrB1b*	*MsrB1b*	*MsrB1b*	*MsrB1b*	*MsrB1b*		
	MsrB1c							
	MsrB1d							
	MsrB1e							
	SelS	*SelS*	*SelS*	*SelS*	*SelS*	*SelS*	*SelS*	*SelS*
	SelT1a	*SelT1*	*SelT1*	*SelT1*	*SelT1*	*SelT1*	*SelT1*	*SelT1*
				SelT1b				
	SelT2	*SelT2*	*SelT2*	*SelT2*	*SelT2*	*SelT2*		
							SelV	*SelV*

	SelUa	*SelU1*	*SelU1*	*SelU1*	*SelU1*	*SelU1*	*SelU1*	*SelU1*
	SelUb		*SelU1b*			*SelU1b*		
	SelUb2	*SelU1c*	*SelU1c*	*SelU1c*	*SelU1c*	*SelU1c*		
				SelW1			*SelW1*	*SelW1*
	SelW2	*SelW2*	*SelW2*	*SelW2*	*SelW2*	*SelW2*		
				SelW2b				
		SelW2c	*SelW2c*		*SelW2c*			
	SPS2	*SPS2a*	*SPS2a*	*SPS2a*	*SPS2a*	*SPS2a*		
							SPS2b	*SPS2b*
	TR2	*TR1*	*TR1*	*TR1*	*TR1*	*TR1*	*TR1*	*TR1*
	TR3	*TR3*	*TR3*	*TR3*	*TR3*	*TR3*	*TR3*	*TR3*
**Total**	40	35	36	38	36	35	25	24

Genes are abbreviated as *DI*: Iodothyroninedeiodinase; *GPx*: Glutathione peroxidase; *Fep15*: fish 15 kDa selenoprotein-like protein; *Sel*: selenoprotein; *Msr*: Methionine sulfoxide reductase; *SPS*: Selenophosphatesynthetase; *TR*: Thioredoxin reductase.
