# Supplementary material for: Genome Sequencing of the Perciform Fish Larimichthys crocea Provides Insights into Molecular and Genetic Mechanisms of Stress Adaptation
Source: PLoS Genet. 2015 Apr 2;11(4):e1005118. doi: 10.1371/journal.pgen.1005118 (PMC4383535; doi:10.1371/journal.pgen.1005118)
Supplement: S21 Table — (PDF) [file pgen.1005118.s040.pdf]

Table S21: Characterization of the *L. crocea* immune system

	Categories	Gene number
**Innate immunity**	Pattern recognition receptors	179
	Antimicrobial peptides	17
	Complement system	119
	Lectins	265
	Interferon system	70
	Interleukin -1 family	29
	Tumor necrosis factor family	13
	Scavenger receptor family	17
	Immune negative regulators	18
	Immune signalling factors	30
	Chemokines	62
**Total**		819
**Adaptive immunity**	Antigen-presentation system	176
	T-cell lineage markers	168
	B-cell lineage markers	65
	Plasma cell markers	9
	Memory T/B cell markers	62
	T/B cell development related genes	56
	Gene rearrangement-related factors	168
	Immunoglobulins and Ig family members	1005
**Total**		1709
**Total of immune-relevant genes**		2528
