# Supplementary material for: Genome Sequencing of the Perciform Fish Larimichthys crocea Provides Insights into Molecular and Genetic Mechanisms of Stress Adaptation
Source: PLoS Genet. 2015 Apr 2;11(4):e1005118. doi: 10.1371/journal.pgen.1005118 (PMC4383535; doi:10.1371/journal.pgen.1005118)
Supplement: S22 Table — (PDF) [file pgen.1005118.s041.pdf]

Table S22: Number of genes related to immunity in *L. crocea* and other six fish genomes

Gene	*Larimichthys crocea*	*Danio rerio*	*Gadus morhua*	*Gasterosteus aculeatus*	*Oryzias latip*	*Takifugu rubripes*	*Tetraodon nigroviridis*
NLRC3	43	58	1	3	2	6	2
TRIM25	54	80	15	16	42	14	12
IgHV-CAM	38	2	1	12	12	37	19
Mep1b	24	10	9	13	11	14	11
CLEC17A	21	37	9	10	9	8	10
C1ql4	19	14	12	8	9	6	9
Gimap8	18	33	1	3	7	2	0
IFI44	13	17	8	8	3	10	6
EEF1A1	10	3	3	4	3	3	4
LRRC70	8	4	4	4	3	3	4
Mrc1	7	2	3	4	4	4	2
Tnfsf14	5	10	8	1	1	0	0
VTCN1	4	1	0	1	5	0	1
Bax	4	5	2	2	1	2	1
cGAS	3	1	0	0	0	0	0
DDX41	3	1	1	1	1	1	1
IGSF9B	2	1	0	1	0	0	2

Note: Genes are abbreviated as *NLRC3*: NOD-like receptor family CARD domain containing 3; *TRIM25*: tripartite motif-containing protein 25; *IgHV-CAM*: Ig heavy chain V-III region CAM; *Mep1b*: Meprin A subunit beta; *CLEC17A*: C-type lectin domain family 17, member A; *C1ql4*: Complement C1q-like protein 4; *Gimap8*: GTPase IMAP family member 8; *IFI44*: Interferon-induced protein 44; *EF1A1*: Elongation factor 1-alpha 1; *LRRC70*: Leucine-rich repeat-containing protein 70; *Mrc1*: Macrophage mannose receptor 1; *Tnfsf14*: Tumor necrosis factor receptor superfamily member 14; *VTCN1*: V-set domain-containing T-cell activation inhibitor 1; *Bax*: Apoptosis regulator BAX; *cGAS*: Cyclic GMP-AMP synthase; *DDX41*: Probable ATP-dependent RNA helicase DDX41; *IGSF9B*: Protein turtle homolog B.
