# Supplementary material for: Genome Sequencing of the Perciform Fish Larimichthys crocea Provides Insights into Molecular and Genetic Mechanisms of Stress Adaptation
Source: PLoS Genet. 2015 Apr 2;11(4):e1005118. doi: 10.1371/journal.pgen.1005118 (PMC4383535; doi:10.1371/journal.pgen.1005118)
Supplement: S23 Table — (PDF) [file pgen.1005118.s042.pdf]

Table S23: Genes involved in mucin biosynthesis and mucus production

Functional Group		Gene Symbol
**Mucins**		*Muc1, Muc2, Muc4, Muc5B, Muc5AC, Muc15, Muc19*
**PDI**		*PDIA1, PDIA3, PDIA4, PDIA6*
**Glycosyl transferases**	Initiating	*GALNT1, GALNT2, GALNT3, GALNT4, GALNT5, GALNT6, GALNT7, GALNT8, GALNT9, GALNT10, GALNT11, GALNT12, GALNT13, GALNT14*
	Core	*C1GALT1, C1GALT2, GCNT1, GCNT3, GCNT4, GCNT7*
	Elongating	*B3GNT1, B3GNT2, B3GNT3, B3GNT5, B3GNT7, B3GALT1, B3GALT2, B3GALT4, B3GALT6, B4GALT1, B4GALT3, B4GALT4, B4GALT5, B4GALT6, B4GALT7*
	Peripheral	*SIAT1, SIAT2, SIAT4C, SIAT6, SIAT7A, SIAT7B, SIAT7D, SIAT7E, SIAT7F, SIAT8A, SIAT8E, CHST1, CHST2, CHST3, CHST6, CHST7, CHST8, CHST10, CHST11, CHST12, CHST13, CHST15, GAL3ST1, FucT-1*
**Receptors**		*VDRA, VDRB, EGFR*
**RAB**		*RAB1A, RAB2A, RAB3, RAB3A, RAB3B, RAB3C, RAB3D, RAB4A, RAB4B, RAB5, RAB5A, RAB5B, RAB5C, RAB6A, RAB6B, RAB6C, RAB7A, RAB8A, RAB8B, RAB9A, RAB9B, RAB10, RAB11B, RAB12, RAB13, RAB14, RAB15, RAB17, RAB18, RAB19, RAB 20, RAB21, RAB22A, RAB23, RAB24, RAB25, RAB26, RAB27A, RAB27B, RAB28, RAB30, RAB31, RAB32, RAB33B, RAB34, RAB35, RAB36, RAB37, RAB38, RAB39A, RAB39B, RAB40C, RAB44*
**SNARE**		*STX1A, STX1B, STX4, STX5, STX6, STX7, STX8, STX10, STX11, STX12, STX16, STX17, STX18, STX19, STXBP1, STXBP5, VAMP2, VAMP4, VAMP5, VAMP7, VAMP8*
**Ion channels, ion pumps and transporters**		*ATP1A1, ATP1A3, ATP1B3, SLC12A2, SLC12A7, SLC4A2, SLC4A4, SLC4A5, CFTR, ATP2A1, ATP2A2, ATP2A3*
**Regulation factors**		*MARCKS, PKCα, PKCβ, PKCι, PKCθ, PKCζ, PKCδ, PKCη, PKCε*

Genes are abbreviated as *Muc*: mucin; *C1GALT*: Glycoprotein-N-acetylgalactosamine 3-beta-galactosyltransferase; *GALNT*: Polypeptide N-acetylgalactosaminyltransferase; *PDIA*: protein disulfide isomerase family A; *GCNT*: Beta-1,3-galactosyl-O-glycosyl-glycoprotein beta-1,6-N-acetylglucosaminyltransferase; *B3GNT*: UDP-GlcNAc:betaGal beta-1,3-N-acetylglucosaminyltransferase; *B3GALT*: beta-1,3-galactosyltransferase; *SIAT*: CMP-N-acetylneuraminate-beta-1,4-galactoside alpha-2,3-sialyltransferase; *GAL3ST1*: Galactosylceramidesulfotransferase; *FucT-1*: GDP-fucose transporter 1; *VDR*: Vitamin D3 receptor; *EGFR*: Epidermal growth factor receptor; *RAB*: Ras-related protein; *STX*: Syntaxin;

STXBP1: Syntaxin-binding protein 1; *VAMP*: Vesicle-associated membrane protein; *ATP1A*: Sodium/potassium-transporting ATPase subunit alpha; *ATP1B*: Sodium/potassium-transporting ATPase subunit beta; *SLC*: Solute carrier family; *CFTR*: Cystic fibrosis transmembrane conductance regulator; ATP2A: Sarcoplasmic/endoplasmic reticulum calcium ATPase 1. *MARCKS*: Myristoylated alanine-rich C-kinase substrate; *PKC*: Protein kinase C.
