# Supplementary material for: Genome Sequencing of the Perciform Fish Larimichthys crocea Provides Insights into Molecular and Genetic Mechanisms of Stress Adaptation
Source: PLoS Genet. 2015 Apr 2;11(4):e1005118. doi: 10.1371/journal.pgen.1005118 (PMC4383535; doi:10.1371/journal.pgen.1005118)
Supplement: S24 Table — (PDF) [file pgen.1005118.s043.pdf]

Table S24: Summary of MS/MS spectra and proteins identified in the *L. crocea* skin mucus under air exposure

Classification	Number	Percentage (%)
Total Spectra	636,059	100.00
Identified Spectra	171,349	26.94
Identified Peptides	25,026	3.93
Identified Proteins	4,489	17.67
Identified Ptoteins (Unique peptides > 2)	3,209	12.63
