# Supplementary material for: Genome Sequencing of the Perciform Fish Larimichthys crocea Provides Insights into Molecular and Genetic Mechanisms of Stress Adaptation
Source: PLoS Genet. 2015 Apr 2;11(4):e1005118. doi: 10.1371/journal.pgen.1005118 (PMC4383535; doi:10.1371/journal.pgen.1005118)
Supplement: S25 Table — (PDF) [file pgen.1005118.s044.pdf]

Table S25: Antioxidant proteins identified in the *L. crocea* mucus proteome

Protein name	
Cytochrome c oxidase subunit 4 isoform 1, mitochondrial	Epidermis-type lipoxygenase 3
Cytochrome c oxidase subunit 5A, mitochondrial	Epidermis-type lipoxygenase 3
Cytochrome c oxidase subunit 5A, mitochondrial	Epidermis-type lipoxygenase 3
Cytochrome c oxidase subunit 6B1	Prolyl 3-hydroxylase 1
Cytochrome c oxidase subunit 6A, mitochondrial	Ceruloplasmin
Cytochrome c oxidase subunit 7A-related protein, mitochondrial	NADH dehydrogenase [ubiquinone] flavoprotein 2, mitochondrial
Phenylalanine-4-hydroxylase	L-amino-acid oxidase
Ubiquinone biosynthesis monooxygenase COQ6	Extended synaptotagmin-2-A
Prostaglandin G/H synthase 1	Zinc finger protein AEBP2
Phospholipid hydroperoxide glutathione peroxidase, mitochondrial	NADH dehydrogenase flavoprotein 1, mitochondrial
Eosinophil peroxidase	Glutathione reductase, mitochondrial
Eosinophil peroxidase	NADH dehydrogenase iron-sulfur protein 4, mitochondrial
Phosphatidylinositol-4-phosphate 5-kinase type-1 gamma	ERO1-like protein alpha
Glutathione peroxidase 1	ERO1-like protein beta
Glutathione peroxidase 1	Malate dehydrogenase, cytoplasmic
Glutathione peroxidase 7	Malate dehydrogenase, cytoplasmic
Thioredoxin-like protein 1	Malate dehydrogenase, mitochondrial
Thioredoxin	Uricase
Thioredoxin domain-containing protein 5	Ubiquitin-conjugating enzyme E2 variant 3
Thioredoxin, mitochondrial	Glutamate dehydrogenase 1, mitochondrial
Superoxide dismutase [Cu-Zn]	Glutamate dehydrogenase, mitochondrial
NADH dehydrogenase iron-sulfur protein 2, mitochondrial	Hydroxyacyl-coenzyme A dehydrogenase, mitochondrial
Prostamide/prostaglandin F synthase	Lambda-crystallin homolog
Peroxiredoxin-6	Protein disulfide-isomerase
Peroxiredoxin	Protein disulfide-isomerase A3
Peroxiredoxin	Protein disulfide-isomerase
Thioredoxin-dependent peroxide reductase, mitochondrial	Protein disulfide-isomerase A5
Peroxiredoxin-4	Protein disulfide-isomerase A4
L-2-hydroxyglutarate dehydrogenase, mitochondrial	Protein disulfide-isomerase A6
L-lactate dehydrogenase B chain	Protein disulfide-isomerase TMX3
L-lactate dehydrogenase A chain	Mitochondrial sodium/hydrogen exchanger 9B2
Peroxiredoxin-5, mitochondrial	Cytochrome b-c1 complex subunit Rieske, mitochondrial
Apoptosis-inducing factor 1, mitochondrial	NADH dehydrogenase iron-sulfur protein 7, mitochondrial
Extracellular superoxide dismutase [Cu-Zn]	NA
Amine oxidase [flavin-containing] A	Dihydropteridine reductase
NADH-cytochrome b5 reductase 3	Hydroxysteroid dehydrogenase-like protein 2
Alpha-aminoadipicsemialdehyde synthase, mitochondrial	Peroxisomal multifunctional enzyme type 2
Alcohol dehydrogenase [NADP(+)] B	Carbonyl reductase [NADPH] 1
Aflatoxin B1 aldehyde reductase member 2	15-hydroxyprostaglandin dehydrogenase [NAD(+)]
Uncharacterized oxidoreductase MSMEG_2408	3-hydroxyacyl-CoA dehydrogenase type-2
Aldo-keto reductase family 1 member B10	3-oxoacyl-[acyl-carrier-protein] reductase FabG
Aldo-keto reductase family 1 member B10	Dehydrogenase/reductase SDR family member 12
Aldo-keto reductase family 1 member B10	Dehydrogenase/reductase SDR family member 11
Sulfide:quinoneoxidoreductase, mitochondrial	C-factor
UDP-glucose 6-dehydrogenase	2,4-dienoyl-CoA reductase, mitochondrial
Glyceraldehyde 3-phosphate dehydrogenase, testis-specific	Dehydrogenase/reductase SDR family member 11
Glyceraldehyde 3-phosphate dehydrogenase, testis-specific	Estradiol 17-beta-dehydrogenase 12-A
Glyceraldehyde-3-phosphate dehydrogenase	Estradiol 17-beta-dehydrogenase 12-B
Prenylcysteine oxidase	Retinol dehydrogenase 12
Mitochondrial peptide methionine sulfoxide reductase	Peroxisomal 2,4-dienoyl-CoA reductase

Mitochondrial peptide methionine sulfoxide reductase	Peroxisomal trans-2-enoyl-CoA reductase
Isocitrate dehydrogenase [NAD] subunit gamma 1, mitochondrial	Pyruvate dehydrogenase E1 component subunit alpha, somatic form, mitochondrial
Cytosolic 10-formyltetrahydrofolate dehydrogenase	Retinol dehydrogenase 3
Isocitrate dehydrogenase [NADP] cytoplasmic	Dehydrogenase/reductase SDR family member 13
Isocitrate dehydrogenase [NADP], mitochondrial	Malate dehydrogenase
Isocitrate dehydrogenase [NADP], mitochondrial	Alpha-aminoadipicsemialdehyde dehydrogenase
Epidermis-type lipoxygenase 3	Aldehyde dehydrogenase, mitochondrial
Isocitrate dehydrogenase [NAD] subunit alpha, mitochondrial	Aldehyde dehydrogenase family 9 member A1
Isocitrate dehydrogenase [NAD] subunit beta, mitochondrial	Aldehyde dehydrogenase, mitochondrial
Dehydrogenase/reductase SDR family member 4 (Fragment)	Delta-1-pyrroline-5-carboxylate dehydrogenase, mitochondrial
Branched-chain-amino-acid aminotransferase, cytosolic	Aldehyde dehydrogenase family 9 member A1-B
Calcium-transporting ATPase type 2C member 1	Aldehyde dehydrogenase family 16 member A1
Trifunctional enzyme subunit alpha, mitochondrial	Retinal dehydrogenase 2
Alkyldihydroxyacetonephosphate synthase, peroxisomal	C-terminal-binding protein 1
Prolyl 4-hydroxylase subunit alpha-1	Glyoxylate reductase/hydroxypyruvate reductase
Very long-chain specific acyl-CoA dehydrogenase, mitochondrial	C-terminal-binding protein 1
Long-chain specific acyl-CoA dehydrogenase, mitochondrial	Medium-chain specific acyl-CoA dehydrogenase, mitochondrial
Glutaryl-CoA dehydrogenase, mitochondrial	2-oxoisovalerate dehydrogenase subunit alpha, mitochondrial
Isovaleryl-CoA dehydrogenase, mitochondrial	Alcohol dehydrogenase class-3 chain L
Short-chain specific acyl-CoA dehydrogenase, mitochondrial	Quinone oxidoreductase
Short/branched chain specific acyl-CoA dehydrogenase, mitochondrial	Synaptic vesicle membrane protein VAT-1 homolog
Aldehyde dehydrogenase family 3 member B1	Synaptic vesicle membrane protein VAT-1 homolog
Fatty aldehyde dehydrogenase	Sorbitol dehydrogenase
Fatty aldehyde dehydrogenase	Prostaglandin reductase 1
Sarcosine dehydrogenase, mitochondrial	Zinc-binding alcohol dehydrogenase domain-containing protein 2
Dihydrolipoyl dehydrogenase, mitochondrial	Alcohol dehydrogenase 1
NADH-cytochrome b5 reductase 2	Quinone oxidoreductase-like protein 1
Delta-1-pyrroline-5-carboxylate synthase	Alcohol dehydrogenase class-3
Glycerol-3-phosphate dehydrogenase [NAD(+)], cytoplasmic	NAD(P) transhydrogenase, mitochondrial
Glycerol-3-phosphate dehydrogenase [NAD(+)], cytoplasmic	Glutaredoxin-1
Glycerol-3-phosphate dehydrogenase [NAD(+)], cytoplasmic	Glutaredoxin-2, mitochondrial
Glycerol-3-phosphate dehydrogenase, mitochondrial	Prostaglandin E synthase 2
NADP-dependent malic enzyme, mitochondrial	Glutaredoxin 3
NAD-dependent malic enzyme, mitochondrial	Glutaredoxin-related protein 5, mitochondrial
Probable 2-oxoglutarate dehydrogenase E1 component DHKTD1, mitochondrial	NADH-ubiquinone oxidoreductase 75 kDa subunit, mitochondrial
Methylmalonate-semialdehyde dehydrogenase, mitochondrial	NAD(P)H dehydrogenase [quinone] 1
Methylmalonate-semialdehyde dehydrogenase, mitochondrial	Succinate dehydrogenase flavoprotein subunit, mitochondrial
2-oxoglutarate dehydrogenase, mitochondrial	Putative oxidoreductase GLYR1
2-oxoglutarate dehydrogenase-like, mitochondrial	D-3-phosphoglycerate dehydrogenase
NADP-dependent malic enzyme	Prolyl 4-hydroxylase subunit alpha-1
3-hydroxyisobutyrate dehydrogenase, mitochondrial	Prolyl 4-hydroxylase subunit alpha-2
3-hydroxyisobutyrate dehydrogenase, mitochondrial	Glutathione S-transferase kappa 1
Arachidonate 15-lipoxygenase B	Thioredoxin reductase 3 (Fragment)
Epidermis-type lipoxygenase 3	Deleted in malignant brain tumors 1 protein
