# Supplementary material for: Genome Sequencing of the Perciform Fish Larimichthys crocea Provides Insights into Molecular and Genetic Mechanisms of Stress Adaptation
Source: PLoS Genet. 2015 Apr 2;11(4):e1005118. doi: 10.1371/journal.pgen.1005118 (PMC4383535; doi:10.1371/journal.pgen.1005118)
Supplement: S26 Table — (PDF) [file pgen.1005118.s045.pdf]

Table S26: Oxygen binding-related proteins identified in the *L. crocea* mucus proteome

Gene ID	Protein name
Lcro_GLEAN_10000061	Hemoglobin subunit alpha-1
Lcro_GLEAN_10005683	Hemoglobin subunit beta
Lcro_GLEAN_10008557	Hemoglobin subunit beta
Lcro_GLEAN_10008556	Hemoglobin subunit alpha-A
Lcro_GLEAN_10011954	Hemoglobin subunit beta-2
Lcro_GLEAN_10005682	Hemoglobin subunit alpha
Lcro_GLEAN_10011722	Cytoglobin-1
Lcro_GLEAN_10011953	Hemoglobin subunit alpha-D
