# Supplementary material for: Genome Sequencing of the Perciform Fish Larimichthys crocea Provides Insights into Molecular and Genetic Mechanisms of Stress Adaptation
Source: PLoS Genet. 2015 Apr 2;11(4):e1005118. doi: 10.1371/journal.pgen.1005118 (PMC4383535; doi:10.1371/journal.pgen.1005118)
Supplement: S27 Table — (PDF) [file pgen.1005118.s046.pdf]

Table S27: **Immunity-related proteins identified in the *L. crocea* mucus proteome**

Gene ID	Protein name
Lcro_GLEAN_10010544	Cathepsin B
Lcro_GLEAN_10000247	Cathepsin D
Lcro_GLEAN_10019933	Cathepsin D
Lcro_GLEAN_10006830	Cathepsin F
Lcro_GLEAN_10007429	Cathepsin K
Lcro_GLEAN_10010056	Cathepsin L
Lcro_GLEAN_10007394	Cathepsin L1
Lcro_GLEAN_10007428	Cathepsin S
Lcro_GLEAN_10001281	Cathepsin Z
Lcro_GLEAN_10012749	C-C motif chemokine 25
Lcro_GLEAN_10012720	C-C motif chemokine 4
Lcro_GLEAN_10000925	CD166 antigen homolog A
Lcro_GLEAN_10022694	CD2-associated protein
Lcro_GLEAN_10021102	CD59 glycoprotein
Lcro_GLEAN_10020957	CD63 antigen
Lcro_GLEAN_10007636	CD81 antigen
Lcro_GLEAN_10014383	CD9 antigen
Lcro_GLEAN_10022556	Complement C1q and tumor necrosis factor-related protein 9
Lcro_GLEAN_10014898	Complement C1q subcomponent subunit C
Lcro_GLEAN_10003747	Complement C1q tumor necrosis factor-related protein 4
Lcro_GLEAN_10008637	Complement C1q-like protein 4
Lcro_GLEAN_10018325	Complement C1r-A subcomponent
Lcro_GLEAN_10018326	Complement C1s subcomponent
Lcro_GLEAN_10005143	Complement C3 (Fragment)
Lcro_GLEAN_10004550	Complement C3 (Fragment)
Lcro_GLEAN_10004551	Complement C3 (Fragment)
Lcro_GLEAN_10001592	Complement C3 (Fragment)
Lcro_GLEAN_10016221	Complement C3 (Fragment)
Lcro_GLEAN_10013690	Complement C4-B
Lcro_GLEAN_10005696	Complement component 1 Q subcomponent-binding protein, mitochondrial
Lcro_GLEAN_10010837	Complement component C6
Lcro_GLEAN_10010838	Complement component C7
Lcro_GLEAN_10012074	Complement component C7
Lcro_GLEAN_10004265	Complement component C8 alpha chain
Lcro_GLEAN_10004266	Complement component C8 beta chain
Lcro_GLEAN_10024705	Complement component C8 gamma chain
Lcro_GLEAN_10023871	Complement component C9
Lcro_GLEAN_10025891	Complement factor B
Lcro_GLEAN_10012700	Complement factor D
Lcro_GLEAN_10004839	Complement factor H

Lcro_GLEAN_10004869	Complement factor H
Lcro_GLEAN_10002965	Heat shock 70 kDa protein 14
Lcro_GLEAN_10017340	Heat shock 70 kDa protein 4
Lcro_GLEAN_10015146	Heat shock 70 kDa protein 4
Lcro_GLEAN_10008388	Heat shock cognate 70 kDa protein
Lcro_GLEAN_10008390	Heat shock cognate 71 kDa protein
Lcro_GLEAN_10007537	Heat shock protein 75 kDa, mitochondrial
Lcro_GLEAN_10022324	Heat shock protein HSP 90-alpha
Lcro_GLEAN_10015783	Heat shock protein HSP 90-beta
Lcro_GLEAN_10006147	Ig heavy chain V region 5-84
Lcro_GLEAN_10006137	Ig heavy chain V region 5A
Lcro_GLEAN_10006149	Ig heavy chain V-III region CAM
Lcro_GLEAN_10006141	Ig heavy chain V-III region HIL
Lcro_GLEAN_10000739	Ig kappa chain C region
Lcro_GLEAN_10012845	Ig kappa chain V-III region MOPC 63
Lcro_GLEAN_10000738	Ig kappa chain V-IV region JI
Lcro_GLEAN_10025384	Ig lambda chain V-III region LOI
Lcro_GLEAN_10000744	Ig lambda-6 chain C region
Lcro_GLEAN_10025386	Ig lambda-6 chain C region
Lcro_GLEAN_10006127	Ig mu chain C region membrane-bound form
Lcro_GLEAN_10004635	Immunoglobulin lambda-like polypeptide 5
Lcro_GLEAN_10021327	Immunoglobulin superfamily member 3
Lcro_GLEAN_10008198	Lysozyme C
Lcro_GLEAN_10008196	Lysozyme C
Lcro_GLEAN_10016863	Lysozyme g
Lcro_GLEAN_10002658	Mannose-specific lectin
Lcro_GLEAN_10019420	Beta-galactoside-binding lectin
Lcro_GLEAN_10018344	Collectin-12
Lcro_GLEAN_10005274	C-type lectin domain family 4 member E
Lcro_GLEAN_10024969	Epiplakin
Lcro_GLEAN_10020509	Fish-egg lectin
Lcro_GLEAN_10000104	Fish-egg lectin
Lcro_GLEAN_10000262	Fucolectin-1
Lcro_GLEAN_10008595	Galectin-3
Lcro_GLEAN_10025411	Galectin-3-binding protein A
Lcro_GLEAN_10008735	Galectin-8
Lcro_GLEAN_10002304	Galectin-9
Lcro_GLEAN_10015893	L-rhamnose-binding lectin CSL2
Lcro_GLEAN_10018984	Malectin
Lcro_GLEAN_10026183	N-acetylaspartatesynthetase
Lcro_GLEAN_10025269	Plasma kallikrein
Lcro_GLEAN_10023966	Plectin
