# Supplementary material for: Genome Sequencing of the Perciform Fish Larimichthys crocea Provides Insights into Molecular and Genetic Mechanisms of Stress Adaptation
Source: PLoS Genet. 2015 Apr 2;11(4):e1005118. doi: 10.1371/journal.pgen.1005118 (PMC4383535; doi:10.1371/journal.pgen.1005118)
Supplement: S28 Table — (PDF) [file pgen.1005118.s047.pdf]

Table S28: Number of ion binding-related proteins identified in the *L. crocea* mucus proteome

GO ID	GO Term	Number
GO:0000287	magnesium ion binding	24
GO:0005509	calcium ion binding	102
GO:0006826	iron ion transport	4
GO:0005506	iron ion binding	28
GO:0005507	copper ion binding	2
GO:0008270	zinc ion binding	159
GO:0006820	anion transport	8
GO:0006812	cation transport	15
GO:0030001	metal ion transport	3
GO:0046872	metal ion binding	24
GO:0030145	manganese ion binding	7
