# Supplementary material for: Genome Sequencing of the Perciform Fish Larimichthys crocea Provides Insights into Molecular and Genetic Mechanisms of Stress Adaptation
Source: PLoS Genet. 2015 Apr 2;11(4):e1005118. doi: 10.1371/journal.pgen.1005118 (PMC4383535; doi:10.1371/journal.pgen.1005118)
Supplement: S29 Table — (PDF) [file pgen.1005118.s048.pdf]

Table S29: Summary of the data for transcriptomes under hypoxia and read alignment ratios to *L. crocea* genome and its CDS

Sample	Insert size (bp)	Read length (bp)	Number of Clean reads	Clean bases (bp)	Alignment to Genome（%）	Alignment to CDS（%）
Br_0 h	200	90_90	55,205,116	4,968,460,440	74.37%	31.96%
Br_1 h	200	90_90	44,989,164	4,049,024,760	73.27%	36.56%
Br_3 h	200	90_90	48,259,124	4,343,321,160	74.66%	33.10%
Br_6 h	200	90_90	45,383,428	4,084,508,520	74.45%	23.98%
Br_12 h	200	90_90	49,398,276	4,445,844,840	74.43%	34.26%
Br_24 h	200	90_90	54,214,458	4,879,301,220	74.13%	34.57%
Br_48 h	200	90_90	55,469,870	4,992,288,300	74.40%	34.73%

CDS: coding sequences of *L. crocea* genome, which were produced from genome-wide prediction of protein-coding genes.
