# Supplementary material for: Genome Sequencing of the Perciform Fish Larimichthys crocea Provides Insights into Molecular and Genetic Mechanisms of Stress Adaptation
Source: PLoS Genet. 2015 Apr 2;11(4):e1005118. doi: 10.1371/journal.pgen.1005118 (PMC4383535; doi:10.1371/journal.pgen.1005118)
Supplement: S30 Table — (PDF) [file pgen.1005118.s049.pdf]

Table S30: Primer sequences for real-time PCR

Gene	Forward Primer(5’----3’)	Reverse Primer(5’----3’)
β-actin	GACCTGACAGACTACCTCATG	AGTTGAAGGTGGTCTCGTGGA
CRF	CCAAACCCAAAACTCCCA	GCCCTGATGTTCCCAACT
CRFR1	TGCATCGGCTGGTGTATA	GAGGCTCGCAGTTTGGTC
POMC	AAGTCTACACCTCCAACGGC	CTTGTACGTGCCGTCCTTCT
IL-6	GCTGTTCTCAAGTATGTGGC	TGTAAATAGTGGGTGTGTCG
ET-1	CATCATCTGGGTCAACAC	GAGGATCACTAAGTCTGG
ADM	TCCTTCCTTTACTGCTGT	TGGTTGGCTGAGTCTTCA
SOCS-3	CAGACCCCCAAAATACCCAA	GGAATCCTCTCTCCACCGCT
SOCS-1	GGAGGAGGCACACGAAATAC	GCAGAGTGAAGAGTGAAGCA
TRH	ACCACGATGGGACACATTCC	CAACTCAGCGAGGTCTTCGT
TRHR	CACCCTACCTGAATGCCTGG	CATTGCTATTGGCCTGCGAG
TSHβ	GGTTTGGGAGGACCTTCCTG	CATCGGTCGTGAGAGACTCG
TRα	CTCATGAGCTCAGACCGTTCA	GAAGACCTCCAGGAAAAGCGG
IDH	GAGGAAATATGCCACGCAGC	TTGCCAGAGCAGAGTCGATG
SCS	TCCCTCTGGTGGTTTGCATC	TGGTGCACAGCTTCGTATGT
FH	CTTCACCAACAACTGCGTGG	GTCAAACTGCTCCTCGGTCA
ALDOA	GCAAACGTTCTTGCACGCTA	TACTTGGTGGAGCAGCTGTG
