# Supplementary material for: Genome Sequencing of the Perciform Fish Larimichthys crocea Provides Insights into Molecular and Genetic Mechanisms of Stress Adaptation
Source: PLoS Genet. 2015 Apr 2;11(4):e1005118. doi: 10.1371/journal.pgen.1005118 (PMC4383535; doi:10.1371/journal.pgen.1005118)
Supplement: S6 Fig — Crystallin protein sequences of zebrafish were used to predict crystallin genes in seven other fish species. The phylogenetic tree was constructed by the maximum likelihood method in PAML. Crystallin genes crygm2b, cryba1, and crybb3, which encode proteins that maintain the transparency and refractive index of the lens, were significantly expanded in the genome of L. crocea relative to those of other sequenced teleosts. The khaki, orange, gold, grey, plum, wheat, and pink backgrounds represent crystallin genes in the genomes of medaka, Atlantic cod, zebrafish, green spotted pufferfish, three spined stickleback, Japanese pufferfish, and large yellow croaker respectively. (PDF) [file pgen.1005118.s006.pdf]

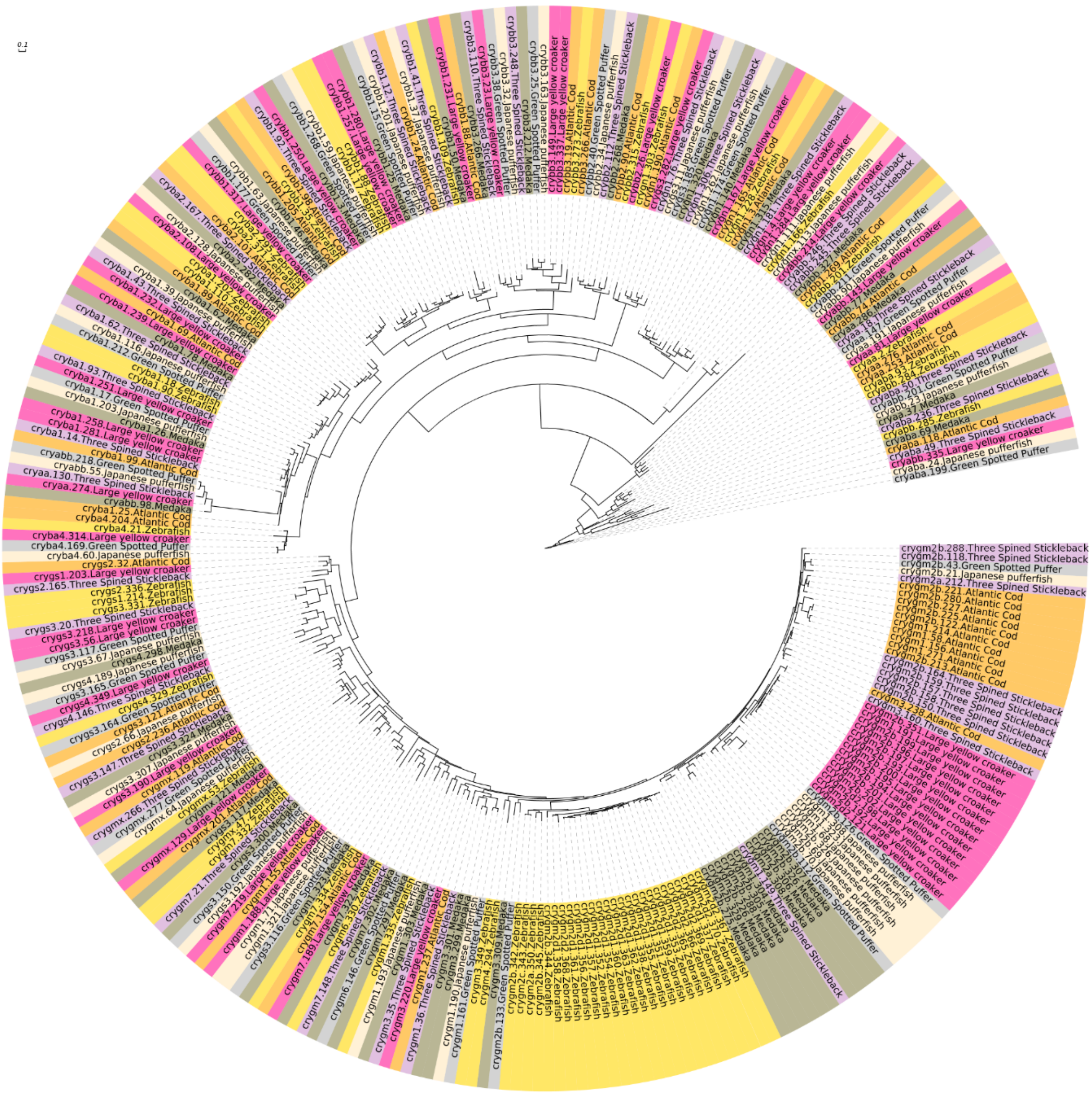
0.1
cryaba.199.Green Spotted Puffer
cryabb.24.Japanese pufferfish
cryabb.335.Large yellow croaker
cryaba.49.Three Spined Stickleback
crygm2b.288.Three Spined Stickleback
crygm2b.118.Three Spined Stickleback
crygm2b.43.Green Spotted Puffer
crygm2b.21.Japanese pufferfish
crygm2a.212.Three Spined Stickleback
crygm2b.280.Atlantic Cod
crygm2b.227.Atlantic Cod
cryba4.314.Large yellow croaker
cryba4.169.Green Spotted Puffer
cryba4.60.Japanese pufferfish
crygs2.32.Atlantic Cod
crygs1.203.Large yellow croaker
crygs2.165.Three Spined Stickleback
