# Supplementary material for: Genome Sequencing of the Perciform Fish Larimichthys crocea Provides Insights into Molecular and Genetic Mechanisms of Stress Adaptation
Source: PLoS Genet. 2015 Apr 2;11(4):e1005118. doi: 10.1371/journal.pgen.1005118 (PMC4383535; doi:10.1371/journal.pgen.1005118)
Supplement: S1 Text — (PDF) [file pgen.1005118.s050.pdf]

Text S1 Background materials

Jingqun Ao[1†], Yinnan Mu[1†], Li-Xin Xiang[2†], DingDing Fan[3†], MingJi Feng[3†], Shicui Zhang[4†], Qiong Shi[3], Lv-Yun Zhu[2], Ting Li[1], Yang Ding[1], Li Nie[2], Qiuhua Li[1], Wei-ren Dong[2], Liang Jiang[5], Bing Sun[4], XinHui Zhang[3], Mingyu Li[1], Hai-Qi Zhang[2], ShangBo Xie[3], YaBing Zhu[3], XuanTing Jiang[3], Xianhui Wang[1], Pengfei Mu[1], Wei Chen[1], Zhen Yue[3], Zhuo Wang[3], Jun Wang[3*], Jian-Zhong Shao[2*] and Xinhua Chen[1*]

[1] Key Laboratory of Marine Biogenetics and Resources, Third Institute of Oceanography, State Oceanic Administration; Fujian Collaborative Innovation Center for Exploitation and Utilization of Marine Biological Resources; Key Laboratory of Marine Genetic Resources of Fujian Province, Xiamen 361005, P.R. China

[2] College of Life Sciences, ZheJiang University; Key Laboratory for Cell and Gene Engineering of Zhejiang Province, Hangzhou 310058, ZheJiang, P.R. China

[3] BGI-Tech, BGI-Shenzhen, Shenzhen 518083, Guangdong, P.R. China

[4] Ocean University of China, Qingdao 266100, Shandong, P.R. China

[5] College of Life Sciences, Shenzhen University, Shenzhen 518060, Guangdong, P.R. China

[†]These authors contributed equally to the work.

[*]To whom correspondence should be addressed: Email: chenxinhua@tio.org.cn (XC); shaojz@zju.edu.cn (JS); wangj@genomics.org.cn (JW).

S1.1 Organism background

The large yellow croaker *Larimichthys crocea* (*L. crocea*) is a temperate-water migratory fish belonging to order Perciformes and family Sciaenidae. Its wild population is mainly distributed in the southern Yellow Sea, East China Sea, and northern South China Sea. *L. crocea* has a flat and long body with yellow or golden-yellow skin. It feeds on various groups of smaller fish and also on marine crustaceans such as shrimp and crab. *L. crocea* is one of the most economically important marine fish in China and East Asian countries due to its rich nutrients and trace elements, such as selenium. In China, the annual yield from *L. crocea* aquaculture exceeds that of any other net-cage-farmed marine fish species [1,2]. *L. crocea* also exhibits peculiar behavioral and physiological characteristics, such as loud sound production, high sensitivity to sound, and well-developed photosensitive and olfactory systems [1,3]. Most importantly, *L. crocea* is especially sensitive to various environmental stresses, such as hypoxia and air exposure. For example, the response of *L. crocea* brain to hypoxia is quick and robust, and a large amount of mucus is secreted from its skin when it is exposed to air [4], although *L. crocea* is not exposed to these stress conditions in nature or with standard aquaculture practices. These traits may render *L. crocea* a good model for investigating the response mechanisms to environmental stress. Several studies have reported transcriptomic and proteomic responses of *L. crocea* to pathogenic infections or immune stimuli [2,5,6]. The effect of hypoxia on the blood physiology of *L. crocea* has been evaluated [4]. However, little is known about the molecular response mechanisms of *L. crocea* against environmental stress. Additionally, *L. crocea* genome consists of 48 telocentromeric chromosomes, and has a high level of heterozygosity as estimated by k-mer

analyses (**Figure S1-S2**).

S1.2 Genome sequence and assembly

S1.2.1 Sample preparation and sequencing

The studies were carried out in strict accordance with the Regulations of the Administration of Affairs Concerning Experimental Animals established by the Fujian Provincial Department of Science and Technology. Animal experiments were approved by the Animal Care and Use Committee of the Third Institute of Oceanography, State Oceanic Administration. All surgery was performed under Tricaine-S anesthesia, and all efforts were made to minimize suffering.

Wild individuals of *L. crocea* were collected from the Sanduao Sea area, Ningde, Fujian, China. Genomic DNA was isolated from the blood of a female fish for BAC library construction using standard molecular biology techniques. BAC-to-BAC strategy and whole-genome shotgun (WGS) methods were combined to obtain a high-quality assembly (**Figure S1**). BAC sequences were merged to build contig sequences, and whole-genome shotgun sequences were used to orient the contigs to scaffold sequences and fill gaps. For each BAC, a library was built with an insert size of 500 bp and sequenced by using the Highseq 2000 system in BGI (Beijing Genomics Institute, Shenzhen, China). For the whole-genome shotgun strategy, a series of libraries with insert sizes from 170 bp to 40 kbp were built. To facilitate the genome analysis, quality control was performed by trimming low-quality reads and bases, and removing contaminated and duplicated reads to obtain the clean data. The following criteria were used to identify the reads that should be removed:

1. Reads with ≥10% unidentified nucleotides.

2. Reads from short-insert-size libraries having more than 65% bases with Q20≤7, and reads from large-insert-size libraries that contained more than 80% bases with Q20≤7.
3. Reads with more than 10 bp aligned to the adapter sequence, allowing ≤2 bp mismatches.
4. Small-insert-size paired-end reads that overlapped ≥10 bp with the corresponding paired end.
5. Read 1 and read 2 of two paired-end reads that were completely identical (and thus considered to be the products of PCR duplication).

We built 42,528 BACs in 443-well plates, producing a total of 475 Gbp. For each BAC, the average coverage was 63.63×, which was sufficient for a high-quality BAC assembly.

S1.2.2 Genome property estimation by k-mer

A preliminary survey was performed to gain insights into the properties of the *L. crocea* genome by k-mer analyses. The 17-mer analysis suggested that the *L. crocea* genome was 691 Mb (**Figure S2**). The k-mer distribution was bimodal, and the k-mer depth of the first peak (22) was half that of the second (44), implying that the genome of *L. crocea* was rich in heterogeneous sites, which is a serious obstacle for short-read assemblies.

S1.2.3 Genome assembly by a BAC-to-BAC strategy

The BAC-to-BAC strategy resolves heterogeneous sites and repetitive elements in a BAC, which will improve the assembly greatly. In the *L. crocea* genome project, each BAC was assembled by SOAPdenovo with different K, and then the longest N50 size was taken as an optimal assembly. For all BACs, the sequences, great than 500 bp, were added to Rabbit (ftp://ftp.genomics.org.cn/pub/Plutellaxylostella/Rabbit_linux-2.6.18-194.blc.tar.gz). Rabbit uses BLAT to find overlaps between different BACs, and then merges and links different

BACs to obtain a consensus assembly. The configuration file for Rabbit was as follows:

```
[sequence]          BAC.fa
[recursive]         1
[genome_size]       340000000
[cpu]               30
[min_len]           2000
[trim_end]          40
[devide_size]       100000000
[find_queue]        bc.q      test
[overlap_queue]     bc.q      test
[ovl]     5001      4000      0.9     0.9     ov_5001
[ovl]     3001      2500      0.9     0.9     ov_3001
[ovl]     100       90        0.9     0.6     ov_301
```

Then, Jellyfish was used to determine the frequency of K-mers for the ~50× WGS reads from single individuals, using the following commands:

```
gunzip -c wgs.fq.gz | jellyfish count -m 17 -o BAC --timing BAC.time -s
1073741824 -t 32 -c 8 -C /dev/fd/0 1>BAC.log 2>BAC.error
jellyfish merge -v -o BAC.jfBAC_* 1>>BAC.log 2>>BAC.error
jellyfish dump -c -t -o BAC.dumpBAC.jf 1>>BAC.log 2>>BAC.error
jellyfish stats -o BAC.statsBAC.jf 2>>BAC.error
jellyfish histo -t 32 BAC.jf | sed 's/ / /g' >BAC.histo
```

After obtained the k-mer occurrence frequency table "BAC. dump", the redundant

sequences were removed by using the following command:

Tool/Duplicate/TrimDupBAC.dumn 17scaf.fa clean 0.3

After that, scaffolds were assigned by SSPACE-V2.1 [7]. The paired-end information from large-insert-size (2 kbp, 5 kbp, 10 kbp, 20 kbp, and 40 kbp) libraries was used to orient the contigs to the scaffold. Finally, gaps in the assembly were filled by Gapcloser [8]. The contig N50 size was 63.11 kbp and the scaffold N50 size was 1.03 Mbp (**Table S5**).

S1.2.4 Genome assembly evaluation

To examine the integrity of the assembly, the clean reads from 170-bp and 500-bp libraries were aligned to the assembly by using BWA [9]. In total, 95.63% of reads were aligned to the *L. crocea* assembly. The single-base depth distribution was calculated (**Figure S3**), and a peak was observed at half of the value of the expected peak of 52×, suggesting the reluctance of the assemblies. Furthermore, the scaffold sequences with a depth of less than 26 × were checked. However, those sequences totaled 3.4 Mb and there were 102 genes (0.04% of total genes) in those scaffolds. The transcript sequences from transcriptomes of each eleven mixed tissues (**Text S5**) were aligned to the *L. crocea* assembly by BLAT [10] with default parameters to examine the completeness of expression region of genome.

S1.3 Evolutionary analysis

S1.3.1 Gene family analysis

To detect variations in the *L. crocea* genome, we chose nine species (*Larimichthys crocea, Gasterosteus aculeatus, Takifugu rubripes, Tetraodon nigroviridis, Oryzias latipes, Gadus morhua, Danio rerio, Gallus gallus,* and *Homo sapiens*) for comparison. Proteins that were greater than 50 amino acids in size were aligned by BLAST [11] (-p blastp-e 1e-7), and

Treefam [12] was used to construct gene families. A total of 19,283 gene families were estimated in *L. crocea* and other eight species. The 25,387 genes observed in *L. crocea* genome belonged to 14,698 gene families, of which 215 gene families (containing 521 genes) were specific to *L. crocea* (**Table S13**).

S1.3.2 Phylogeny and divergence time estimation

To determine the phylogeny of *L. crocea*, 2,257 single-copy genes from the gene family analysis were aligned by using MUSCLE [13] and the alignments were concatenated as a single data set. A total of 5,319,909 nucleic acid sites were obtained from the alignment. To reduce the error topology of phylogeny by alignment inaccuracies, Gblock [14] (codon model) was used to remove unreliably aligned sites and gaps in the alignment. This method produced 3,180,303 reliable coding sites for phylogeny analysis and 87,943 4-fold degenerate sites (neutral substitution rate per year) to estimate divergence time.

To construct a phylogenetic tree of the nine vertebrate species, 3,180,303 nucleic acid sites were added to TreeBeST and PhyML [15]. A total of 87,943 4-fold degenerate sites were used to estimate divergence time by using the mcmctree in the PAML 3.0 package [15].

S1.3.3 Gene family expansion and contraction

Gene family expansion and contraction analyses were performed by CAFE2 [16]. A random birth and death model were used in CAFE2 to study gene gain and loss in gene families across a user-specified phylogenetic tree. A global parameter λ (lambda), which described both gene birth (λ) and death ($\mu=-\lambda$) rate across all branches in the tree for all gene families, was estimated by using the maximum likelihood method. A conditional *P*-value was calculated for each gene family and families with conditional *P*-values less than 0.01 were

considered to have a significantly accelerated rate of expansion and contraction (**Tables S14-S15**).

S1.3.4 Positively selected genes in *L. crocea*

To determine gene orthology, all protein sequences from six species (*Larimichthys crocea*, *Gasterosteus aculeatus*, *Danio rerio*, *Oryzias latipes*, *Takifugu rubripes*, and *Tetraodon nigroviridis*) were aligned by BLAST [17] (-p blastp-e 1e-5-m 8), and the alignments were linked by solar to resolve the separation by local alignment. Alignments with identity >50 and alignment coverage >50% and reciprocal best hits were defined as orthologous between *L. crocea* and other species. Then, the orthology of six species was determined according to the *L. crocea* orthology. A total of 2,346 genes were identified as orthologous genes in six species.

Inference of positive selection generally takes the multiple sequence alignment input for granted, regardless of uncertainties in the alignment. Because alignment error is an important concern in molecular data analyses, alignments were made by using PRANK [18] in the GUIDANCE [19] pipeline. GUIDANCE filters and masks unreliably aligned positions in sequence alignments before subsequent analysis. The codon sequences (nucleotide sequences coding for proteins) were aligned by using PRANK, the columns were removed with low GUIDANCE scores, and the remaining alignment was used to infer positive selection based on the branch-site dN/dS test by codeml in the PAML 3.0 package [15].

S1.4 Genomic basis for physiological characteristics

S1.4.1 Photosensitivity

We used the protein sequences encoded by vision-related genes in zebrafish to compare the

genomes of *L. crocea* and five other teleosts using BLAST, and found that the copy numbers of some visual genes were expanded in the *L. crocea* genome (**Table S17**). Crystallins are the major structural components and necessary for maintaining transparency in the ocular lens. They are important for survival and regeneration of retinal ganglion cells [20]. In our current study, several crystallin genes, such as *crygm2b*, *cryba1*, and *crybb3* were expanded, with more copy numbers in *L. crocea* than in other sequenced teleosts. The major and most abundant protein present in the ocular lens of most teleosts is *gamma-crystallin* [21], among which *crygam2b* in the *L. crocea* genome was remarkably expanded compared with other teleosts (12 vs. 3-8 copies; **Figure S6**). The specific expansion of these crystallin genes may be helpful for improving photosensitivity by increasing lens transparency, thereby enabling the fish to easily find food and avoid predation underwater. Retinoid dehydrogenases/reductases (RDHs) can reduce the reactive aldehyde from photo- activated rhodopsin by converting all-trans-retinal to all-trans-retinol. Rhodopsin is expressed in rod cells that are used for dim light. After light exposure, the RDH12 in inner segments of photoreceptors can reduce the retinal leak and toxic aldehydes [22]. Therefore, the expansion of RDH12 gene (*rdh12*) in *L. crocea* would be useful for protecting rhodopsin and retina.

S1.4.2 Olfaction.

Chemosensation is essential for survival. The chemical senses are responsible for detecting molecules of immense chemical variety, which requires a massive repertoire of receptors to match the diversity of chemical structures [23]. The olfactory/odorant receptors (ORs) are the most important chemosensory receptors in detecting environmental chemicals, and they detect a wide range of compounds [3]. *L. crocea* possesses powerful olfactory abilities, as the

numerous cilia and microvilli are widely distributed on the olfactory epithelia. The odorant receptor repertoire of vertebrate species, including teleost fishes, has been extensively reported [24-26]. Here, we tried to characterize the OR-like genes in the *L. crocea* genome.

We downloaded the OR-like genes from NCBI and used these for homology searches against the genomes of large yellow croaker, atlanticcod, zebrafish (Zv9), medaka, stickleback, Janpanese pufferfish and green spotted pufferfish using TBLASTN with E-value<1E-5. We chose alignments with coverage ≥30% and identity ≥30% and extended 5 kbp on both ends of every alignment. GeneWise2.2.0 was employed to predict gene structures and open-reading frames (ORF). A sequence was discarded if there was at least one premature stop codon or frame shift. The remaining predicted sequences were re-checked by BLAST searches against the Swissprot database [27] (UniProt Consortium, 2014). Only those proteins that gave an ‘Olfactory receptor’ hit and with a length greater than 270 amino acids were retained and defined as functional OR-like genes. **Table S18** shows the number and classification of identified functional OR-like genes of seven teleost genomes. The zebrafish has the largest number of functional OR-like genes (~152), possibly because of one more round of genome duplication in zebrafish, and the green spotted pufferfish has the least (~44), which agrees with previous studies [3,24].

We identified 112 functional OR-like genes in the *L. crocea* genome (**Table S18**), consistent with a previous report in which 111 OR genes were found to be expressed in the olfactory epithelial tissues of *L. crocea* by transcriptome analysis [3]. A homologous assignment method was used to classify the putative OR-like genes by BLASTP with previously predicted vertebrate OR genes belonging to different groups [24]. Based on the

nomenclature of Niimura, the majority of these genes (66 in *L. crocea*) were classified into the ‘delta’ group, which is involved in perception of water-borne odorants. *L. crocea* also possessed the highest number of genes that were classified into the “eta” group (30, $P < 0.001$, **Table S18**), and these genes may contribute to the olfactory detection abilities, which could be useful for feeding and migration [28].

An OR data set was prepared using putative OR proteins from the seven fish genomes above. Six G protein-coupled receptors (GPCRs), alpha-1B-adrenergic receptor (NP_000670.1), cholinergic receptor, muscarinic 1 (NP_000729.2), somatostatin receptor 5 (NP_001044.1), chemokine-binding protein 2 (NP_001287.2), GPCR 35 (NP_005292.2), and GPCR G2A (NP_037477.1) were also included to serve as outgroups. PhyML was used to generate a maximum likelihood (ML) phylogenetic tree. The ML trees were viewed and edited using evolview [29,30]. A tree circular cladogram for the “eta” group is shown in **Figure S7**.

S1.4.3 Sound perception

L. crocea is named for its ability to generate strong repetitive drumming sounds, especially during reproduction. For good communication, fish have developed high sensitivities to environmental sound. Several important auditory genes, such as otoferlin (*OTOF*), *claudinj*, and otolin 1 (*OTOL1*), were signicantly expanded in the *L. crocea* genome (**Table S19**). OTOF is a key calcium ion sensor involved in the Ca^{2+}-triggered synaptic vesicle-plasma membrane fusion. Claudinj and otolin-1 are essential for the formation of the otoliths and the normal ear function, which have been reported in the studies of zebrafish and medaka [31]. These expansions may contribute to the detection of sound signaling during communication,

and thus to reproduction and survival.

S1.4.4 Selenoproteins

L. crocea is rich in selenium (Se; ~42.57 μg/ 100g). Se is an antioxidant and essential microelement in mammals, as it plays an important role in mitigating oxidative damage of membranes [32]. It is mainly present as selenoproteins. Selenoproteins play roles in regulating metabolic activity, antioxidant defence, immune function, intracellular redox modulation [33,34]. The active site of each selenoprotein is selenocysteine (Sec), which is encoded by UGA, a stop signal in the canonical genetic code. It can be translated into a Sec residue when a stem-loop structure, the Sec insertion sequence (SECIS) element, is located in the 3'-untranslated region (UTR) of a selenoprotein gene in eukaryotes and archaea or located the downstream of the Sec-decoding TGA (designated as Sec-TGA) in bacteria[35-38]. Here, the algorithm SelGenAmic[39] was used to predict selenoprotein genes in the *L. crocea* genome.

Forty selenoprotein genes were identified in the *L. crocea* genome (**Table S20**), which is the highest number in all sequenced vertebrates by far [40]. The variety of the selenoproteome in *L. crocea* was similar to those of other fish species, and only a few selenoprotein families were not shared in common. Interestingly, five copies of *MsrB1*, which encodes methionine sulfoxide reductase, were found in *L. crocea* (*MsrB1a, MsrB1b, MsrB1c, MsrB1d,* and *MsrB1e*), whereas only two copies (*MsrB1a* and *MsrB1b*) were found in other fish, thus suggesting its broader specificity to reduce all possible substrates. *Fep15,* encoding a new selenocysteine-containing member of Sep15 protein family, has been identified only in fish [41], but seemed to be absent in the *L. crocea* genome. Fish have more selenoprotein

genes than other vertebrates [40]. Several selenoprotein gene families were duplicated in teleost fish but not in other investigated vertebrates, most likely owing to the whole-genome duplication in the early evolution of ray-finned fish [40,42].

S1.5 Transcriptome sequencing and analysis

S1.5.1 Transcriptome of the wild male and female *L. crocea*

For accurate gene annotation of the reference assembly, the RNAseq data were generated from eleven tissues (ovary [or testis from male], stomach, kidney, heart, gill, skin, brain, eyes, spleen, intestines, and liver) of a female or a male wild *L. crocea* (120-130 g) obtained from the Sanduao Sea area, Ningde, China. Total RNA was extracted by using Trizol Reagent (Invitrogen, USA) and digested by RNase-free DNase I (TaKaRa, China) to remove genomic DNA. The total RNA from different tissues was mixed up in equal proportions.

Primary sequencing data produced by the Illumina HiSeq 2000, called raw reads, were subjected to quality control (QC) that determined if an RNA resequencing step was needed. After QC, the clean reads were screened from the raw reads and aligned to the *L. crocea* genome and its coding region sequences (CDS) by using SOAPaligner/SOAP2 [43]. The alignment to the CDS of *L. crocea* genome was utilized to calculate the distribution of reads on reference genes and to perform coverage analysis. If an alignment result passed QC (alignment ratio >70%), we proceeded with subsequent analysis, including gene expression calculation and differential expression comparison.

S1.5.2 Detection of differentially expressed genes

The gene expression levels were calculated by using the RPKM method (reads per kilobase transcriptome per million mapped reads). According to the methodology of Audic and Claverie [44], a strict algorithm was developed to identify differentially expressed genes

between two samples, as follows.

The number of unambiguous clean reads (which means the reads in RNAseq) from gene A was denoted as x. Given that every gene's expression occupies only a small part of the library, x has a poisson distribution:

$$p(x) = \frac{e^{-\lambda}\lambda^{x}}{x!} \quad (\lambda \text{ is the real transcripts of the gene})$$

The total clean read number of sample 1 is N_1, and the total clean read number of sample 2 is N_2; gene A holds x reads in sample 1 and y reads in sample 2. The probability that gene A was expressed equally between two samples can be calculated as:

$$2\sum_{i=0}^{i=y} p(i|x)$$

$$\text{or } 2 \times \left(1 - \sum_{i=0}^{i=y} p(i|x)\right) \left(if \sum_{i=0}^{i=y} p(i|x) > 0.5\right)$$

$$p(y|x) = \left(\frac{N_2}{N_1}\right)^{y} \frac{(x+y)!}{x!y!\left(1+\frac{N_2}{N_1}\right)^{(x+y+1)}}$$

The *P*-value corresponds to the differential gene expression test. Because differentially expressed gene (DEG) analysis generates the problem that thousands of hypotheses (gene x is differentially expressed between the two groups) are tested simultaneously, correction for false positives (type I errors) and false negatives (type II errors) was performed using the false discovery rate (FDR) method [45]. FDR≤0.001 and the absolute value of $Log_2Ratio \geq 1$ were used as the threshold to judge the significance of gene expression differences. Expression patterns of key DEGs were analyzed by the open source clustering software Cluster 3.0 and presented using the GraphPad Prism 5 software or Java TreeView.

S1.6 Mucus proteome analysis

S1.6.1 Preparation of mucus proteins

Skin mucus was collected from six healthy *L. crocea* individuals under air exposure as previously described [46]. Briefly, the fish were anesthetised with a sub-lethal dose of Tricaine-S (100 mg/L), and transferred gently to a sterile plastic bag for 3 min to slough off the mucus under air exposure. Proteins were extracted from a pool of skin mucus of six fish by the trichloroacetic acid-acetone precipitation method and then digested by the trypsin gold (Promega, USA) with a ratio of protein: trypsin = 20:1.

Peptides were separated by SCX chromatography using the Shimadzu LC-20AB HPLC Pump system. The peptides from digestion was reconstituted with 4 mL buffer A (25 mM NaH_2PO_4 in 25% ACN, pH 2.7) and loaded onto a 4.6 × 250 mm Ultremex SCX column containing 5-μm particles (Phenomenex). The peptides was eluted at a flow rate of 1 mL/min with a gradient of buffer A for 10 min, 5-35% buffer B (25 mM NaH_2PO_4, 1 M KCl in 25% ACN, pH 2.7) for 11 min, 35-80% buffer B for 1 min. The system was maintained in 80% buffer B for 3 min before equilibrating with buffer A for 10 min. Elution was monitored by measuring absorbance at 214 nm, and fractions are collected every 1 min. The eluted peptides were pooled as 20 fractions, desalted by Strata X C18 column (Phenomenex) and vacuum-dried.

S1.6.2 LC-MS/MS analysis and Protein Identification

Separation and purification was performed based on Triple TOF 5600. Each fraction was resuspended in certain volume of buffer A (2% ACN, 0.1% FA) and centrifuged at 20,000 g for 10 min. In each fraction, the final concentration was about 0.5 μg/μL on average. Ten

microliters of supernatant were loaded on an Shimadzu LC-20AD nanoHPLC by the autosampler onto a 2 cm C18 trap column (inner diameter 200 μm) and the peptides were eluted onto a resolving 10 cm analytical C18 column (inner diameter 75 μm) made in-house. The samples were loaded at 15 μL/min for 4 min, then the 44 min gradient is run at 400 nL/min starting from 2 to 35% B (98% ACN and 0.1% FA), followed by 2 min linear gradient to 80%, and maintenance at 80% B for 4 min, and finally return to 2% in 1 min. Data acquisition was performed with a TripleTOF 5600 System (AB SCIEX, Concord, ON) fitted with a Nanospray III source (AB SCIEX, Concord, ON). Data was acquired using an ion spray voltage of 2.5 kV, curtain gas of 30 PSI, nebulizer gas of 15 PSI, and an interface heater temperature of 150 ℃. The MS was operated with a RP of greater than or equal to 30, 000 FWHM for TOF MS scans. For IDA, survey scans were acquired in 250 ms and as many as 30 product ion scans were collected if exceeding a threshold of 120 counts per second (counts/s) and with a 2^+ to 5^+ charge-state. Total cycle time was fixed to 3.3 s. Q2 transmission window was 100 Da for 100%. Four time bins were summed for each scan at a pulser frequency value of 11 kHz through monitoring of the 40 GHz multichannel TDC detector with four-anode channel detection. A sweeping collision energy setting of 35±5 eV adjust rolling collision energy was applied to all precursor ions for collision-induced dissociation. Dynamic exclusion was set for 1/2 of peak width (18 s), and then the precursor was refreshed off the exclusion list.

Peptide and Protein Identification---All spectra were mapped by MASCOT sever version 2.3.02 against the database of *L. crocea* genome with the parameters as follows: peptide mass tolerance 0.05 Da; fragment mass tolerance 0.1 Da; fixed modification “Carbamidomethyl

(C)”; variable modifications “Gln->pyro-Glu (N-term Q), Oxidation (M), and Deamidation (N, Q)”. The ion score of a peptide matched to a protein must be greater than or equal to the MASCOT identity score, and the peptides must have a length of at least 6 amino acids. For further analysis of the function of the mucus proteome, only the proteins with at least two unique peptides were selected. In total, 25,026 peptides were identified, which belong to 3,209 proteins encoded by *L. crocea* genome. (**Table S24**).

References

1. Su Y (2004) Breeding and Farming of *Pseudosciaena Crocea*. Beijing: China Ocean Press. 329 p.
2. Mu Y, Ding F, Cui P, Ao J, Hu S, et al. (2010) Transcriptome and expression profiling analysis revealed changes of multiple signaling pathways involved in immunity in the large yellow croaker during *Aeromonas hydrophila* infection. BMC Genomics 11: 506.
3. Zhou Y, Yan X, Xu S, Zhu P, He X, et al. (2011) Family structure and phylogenetic analysis of odorant receptor genes in the large yellow croaker (*Larimichthys crocea*). BMC Evol Biol 11: 237.
4. Gu X, Xu Z (2011) Effect of hypoxia on the blood of large yellow croaker (*Pseudosciaena crocea*). Chinese Journal of Oceanology and Limnology 29: 524.
5. Mu Y, Li M, Ding F, Ding Y, Ao J, et al. (2014) De Novo Characterization of the Spleen Transcriptome of the Large Yellow Croaker (*Pseudosciaena crocea*) and Analysis of the Immune Relevant Genes and Pathways Involved in the Antiviral Response. PLoS One 9: e97471.
6. Yu S, Mu Y, Ao J, Chen X (2010) Peroxiredoxin IV regulates pro-inflammatory responses in large yellow croaker (*Pseudosciaena crocea*) and protects against bacterial challenge. J Proteome Res 9: 1424-1436.
7. Boetzer M, Henkel CV, Jansen HJ, Butler D, Pirovano W (2011) Scaffolding pre-assembled contigs using SSPACE. Bioinformatics 27: 578-579.
8. Luo R, Liu B, Xie Y, Li Z, Huang W, et al. (2012) SOAPdenovo2: an empirically improved memory-efficient short-read de novo assembler. Gigascience 1: 18.
9. Li H, Durbin R (2009) Fast and accurate short read alignment with Burrows-Wheeler transform. Bioinformatics 25: 1754-1760.
10. Kent WJ (2002) BLAT--the BLAST-like alignment tool. Genome Res 12: 656-664.
11. Altschul SF, Gish W, Miller W, Myers EW, Lipman DJ (1990) Basic local alignment search tool. J Mol Biol 215: 403-410.
12. Ruan J, Li H, Chen Z, Coghlan A, Coin LJ, et al. (2008) TreeFam: 2008 Update. Nucleic Acids Res 36: D735-740.
13. Edgar RC (2004) MUSCLE: multiple sequence alignment with high accuracy and high

throughput. Nucleic Acids Res 32: 1792-1797.
14. Castresana J (2000) Selection of conserved blocks from multiple alignments for their use in phylogenetic analysis. Mol Biol Evol 17: 540-552.
15. Yang Z (1997) PAML: a program package for phylogenetic analysis by maximum likelihood. Comput Appl Biosci 13: 555-556.
16. De Bie T, Cristianini N, Demuth JP, Hahn MW (2006) CAFE: a computational tool for the study of gene family evolution. Bioinformatics 22: 1269-1271.
17. Mount DW (2007) Using the Basic Local Alignment Search Tool (BLAST). CSH Protoc 2007: pdb top17.
18. Loytynoja A, Goldman N (2010) webPRANK: a phylogeny-aware multiple sequence aligner with interactive alignment browser. BMC Bioinformatics 11: 579.
19. Penn O, Privman E, Ashkenazy H, Landan G, Graur D, et al. (2010) GUIDANCE: a web server for assessing alignment confidence scores. Nucleic Acids Res 38: W23-28.
20. Piri N, Kwong JM, Caprioli J (2013) Crystallins in retinal ganglion cell survival and regeneration. Mol Neurobiol 48: 819-828.
21. Pan FM, Chang WC, Lin CH, Hsu AL, Chiou SH (1995) Characterization of gamma-crystallin from a catfish: structural characterization of one major isoform with high methionine by cDNA sequencing. Biochem Mol Biol Int 35: 725-732.
22. Chen C, Thompson DA, Koutalos Y (2012) Reduction of all-trans-retinal in vertebrate rod photoreceptors requires the combined action of RDH8 and RDH12. J Biol Chem 287: 24662-24670.
23. Mombaerts P (2004) Genes and ligands for odorant, vomeronasal and taste receptors. Nat Rev Neurosci 5: 263-278.
24. Niimura Y (2009) On the origin and evolution of vertebrate olfactory receptor genes: comparative genome analysis among 23 chordate species. Genome Biol Evol 1: 34-44.
25. Niimura Y (2009) Evolutionary dynamics of olfactory receptor genes in chordates: interaction between environments and genomic contents. Hum Genomics 4: 107-118.
26. Alioto TS, Ngai J (2005) The odorant receptor repertoire of teleost fish. BMC Genomics 6: 173.
27. UniProt Consortium (2014) Activities at the Universal Protein Resource (UniProt). Nucleic Acids Res 42: D191-198.
28. Li W, Sorensen PW, Gallaher DD (1995) The olfactory system of migratory adult sea lamprey (*Petromyzon marinus*) is specifically and acutely sensitive to unique bile acids released by conspecific larvae. J Gen Physiol 105: 569-587.
29. Guindon S, Dufayard JF, Lefort V, Anisimova M, Hordijk W, et al. (2010) New algorithms and methods to estimate maximum-likelihood phylogenies: assessing the performance of PhyML 3.0. Syst Biol 59: 307-321.
30. Zhang H, Gao S, Lercher MJ, Hu S, Chen WH (2012) EvolView, an online tool for visualizing, annotating and managing phylogenetic trees. Nucleic Acids Res 40: W569-572.
31. Eisen MD, Ryugo DK (2007) Hearing molecules: contributions from genetic deafness. Cell Mol Life Sci 64: 566-580.
32. Huang Z, Rose AH, Hoffmann PR (2012) The role of selenium in inflammation and

immunity: from molecular mechanisms to therapeutic opportunities. Antioxid Redox Signal 16: 705-743.
33. Papp LV, Holmgren A, Khanna KK (2010) Selenium and selenoproteins in health and disease. Antioxid Redox Signal 12: 793-795.
34. Tinggi U (2008) Selenium: its role as antioxidant in human health. Environ Health Prev Med 13: 102-108.
35. Atkins JF, Gesteland RF (2000) The twenty-first amino acid. Nature 407: 463, 465.
36. Kryukov GV, Kryukov VM, Gladyshev VN (1999) New mammalian selenocysteine-containing proteins identified with an algorithm that searches for selenocysteine insertion sequence elements. J Biol Chem 274: 33888-33897.
37. Bock A (2000) Biosynthesis of selenoproteins--an overview. Biofactors 11: 77-78.
38. Hatfield DL, Gladyshev VN (2002) How selenium has altered our understanding of the genetic code. Mol Cell Biol 22: 3565-3576.
39. Jiang L, Liu Q, Ni J (2010) In silico identification of the sea squirt selenoproteome. BMC Genomics 11: 289.
40. Mariotti M, Ridge PG, Zhang Y, Lobanov AV, Pringle TH, et al. (2012) Composition and evolution of the vertebrate and mammalian selenoproteomes. PLoS One 7: e33066.
41. Novoselov SV, Hua D, Lobanov AV, Gladyshev VN (2006) Identification and characterization of Fep15, a new selenocysteine-containing member of the Sep15 protein family. Biochem J 394: 575-579.
42. Taylor JS, Braasch I, Frickey T, Meyer A, Van de Peer Y (2003) Genome duplication, a trait shared by 22000 species of ray-finned fish. Genome Res 13: 382-390.
43. Li R, Yu C, Li Y, Lam TW, Yiu SM, et al. (2009) SOAP2: an improved ultrafast tool for short read alignment. Bioinformatics 25: 1966-1967.
44. Audic S, Claverie JM (1997) The significance of digital gene expression profiles. Genome Res 7: 986-995.
45. Benjamini Y, Yekutieli D (2001) The control of the false discovery rate in multiple testing under dependency. Annals of statistics: 1165-1188.
46. Subramanian S, Ross NW, MacKinnon SL (2008) Comparison of antimicrobial activity in the epidermal mucus extracts of fish. Comp Biochem Physiol B Biochem Mol Biol 150: 85-92.
